# 3D‐Printed Optical Volatile Organic Compound Sensors Based on Donor‐Substituted Coumarin Thermally Activated Delayed Fluorescence Emitters

**DOI:** 10.1002/smsc.70325

**Published:** 2026-06-21

**Authors:** Sara Paniziutti, Maria Vittoria Piras, Annalisa Chiappone, Enrico Podda, Pier Carlo Ricci, Stefania Porcu, Tomas Matulaitis, Eli Zysman‐Colman, Francesco Secci

**Affiliations:** ^1^ Dipartimento di Scienze Chimiche e Geologiche Università degli Studi di Cagliari, Complesso Universitario di Monserrato Monserrato Cagliari Italy; ^2^ Organic Semiconductor Centre EaStCHEM School of Chemistry University of Street Andrews Fife UK; ^3^ Centro Servizi di Ateneo per la Ricerca – CeSAR Complesso Universitario di Monserrato Monserrato Cagliari Italy; ^4^ Dipartimento di Fisica Università degli Studi di Cagliari Complesso Universitario di Monserrato Monserrato Cagliari Italy

**Keywords:** 3D printing, coumarins, thermally activated delayed fluorescence, volatile organic compounds, volatile organic compound sensors

## Abstract

Volatile organic compounds (VOCs) pose a major environmental and health concern, motivating the development of solid‐state optical sensing materials that are both responsive and easily manufacturable. Here we introduce a new family of donor‐substituted coumarin thermally activated delayed fluorescent emitters and embed them within photocurable acrylate resins. These materials retain strong solid‐state photoluminescence and can be 3D‐printed into functional sensing architectures that exhibit reproducible and analyte‐dependent, intensity‐based optical responses to chemically diverse VOC vapors. This work demonstrates how tailored emitter design and 3D printing can be combined to realize versatile VOC‐responsive optical materials.

## Graphical abstract

Donor–acceptor coumarin emitters displaying thermally activated delayed fluorescence (TADF) are formulated into photocurable resins and processed by digital light processing (DLP) 3D printing. The resulting printed materials show a sensitive and repeatable photoluminescence response when exposed to volatile organic compounds (VOCs).

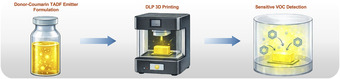

## 1 Introduction

Volatile Organic Compounds (VOCs), such as aliphatic hydrocarbons, aromatic benzene, toluene, ethylbenzene, and xylenes (BTEX) species, chlorinated solvents and oxygenated species (e.g., alcohols, ketones, and esters) originate from chemical manufacturing, fuel combustion, coatings and building materials. VOCs can be harmful even at trace levels, prompting strict WHO, EPA, and EU regulations. VOCs are relevant across a wide range of environments, from indoor air quality to industrial settings, where acceptable concentration thresholds can vary from sub‐ppm to ppm levels depending on the specific compound and application. This highlights the need for sensing technologies that are not only sensitive, but also adaptable to different operating conditions and deployment scenarios [1, 5]. Although analytical techniques such as gas chromatography‐mass spectrometry (GC‐MS), inductively coupled plasma (ICP) spectrometry, and atomic absorption spectroscopy (AAS) provide excellent sensitivity, they are not suitable for rapid and distributed monitoring, as they require bulky instrumentation, trained personnel, and off‐line analysis. This has stimulated interest in the identification and development of optical materials capable of signaling VOC uptake through modification in their photophysical behavior. Optical VOC sensing often exploits VOC‐induced changes in the photophysical properties of the sensor, mediated by analyte uptake into the surrounding matrix and by explicit VOC‐emitter intermolecular interactions.

Photoluminescence (PL)‐based approaches typically exploit the sensitivity of charge‐transfer (CT) excited states to changes in medium polarity, and/or viscosity and/or explicit host–guest interactions. For instance, Jarangdet et al. showed that salicylidene derivatives adsorbed onto cellulose paper can discriminate among 15 solvent vapors through polarity‐dependent emission [6]. Borelli et al. demonstrated that the PL of a julolidine‐based twisted intramolecular CT (TICT) motif covalently incorporated into styrene copolymer films undergoes a vapochromic shift as VOC uptake softens the polymer matrix [7]. Likewise, VOC sorption in luminescent porous hosts can modulate PL through explicit host–guest interactions [8, 10]. These representative studies highlight the effectiveness of using microenvironment‐sensitive emitters for VOC sensing, although in these cases, the use of paper substrates, planar films, or rigid porous networks limits control over emitter dispersion, mechanical robustness and device geometry.

**FIGURE 1:**
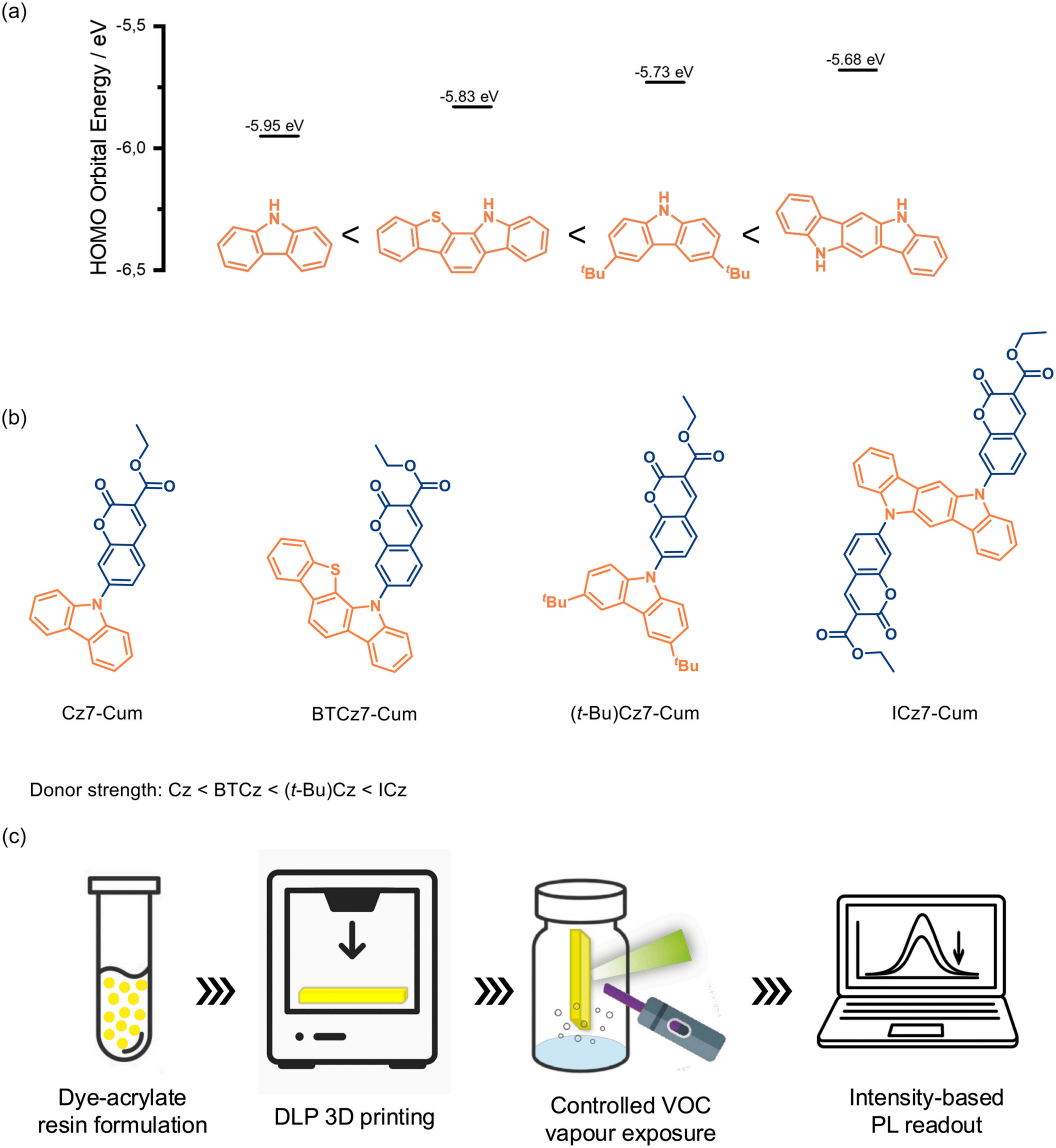
(a) DFT‐calculated HOMO energies of Cz**,** (*t*‐Bu)Cz**,** BTCz, and ICz at the PBE0/6‐31G(d, p) level of theory in the gas phase at the optimized S_0_ geometry (isovalue = 0.02). (b) Chemical structures of the four emitters **Cz7‐Cum**, **BTCz7‐Cum**, **(*t*‐Bu)Cz7‐Cum**, and **ICz7‐Cum** (orange denotes donor unit and blue denotes the coumarin acceptor unit). (c) Schematic illustration of the sensing platform workflow: (1) incorporation of donor‐coumarin emitters into a photocurable acrylate resin; (2) DLP 3D printing; (3) controlled exposure of the printed objects to VOC vapors; and (4) intensity‐based PL readout used to generate VOC response fingerprints.

Beyond matrix‐centered approaches, molecular design offers a complementary strategy to control VOC sensitivity at the level of the emissive unit itself. With donor–acceptor (D–A) emitters, the choice of donor and acceptor units provides a direct design handle to modulate the strength of and electronic coupling between the highest occupied molecular orbital (HOMO) and lowest unoccupied molecular orbital (LUMO) and, as a consequence, the excited‐state energy of the molecule. Tuning this HOMO–LUMO spatial separation affects the singlet–triplet excited‐state energy gap (Δ*E*_ST_) and, when Δ*E*_ST_ is sufficiently small, can enable thermally activated delayed fluorescence (TADF). TADF‐active compounds possess emissive singlet (S_1_) CT states that are intrinsically sensitive to local polarity, and to how strongly the surrounding matrix constrains conformational and environmental relaxation in the excited state, so that even small variations can produce distinct emissive responses [11, 16]. Despite this potential, only a few TADF‐based vapor sensors have been reported [17]. The benzene‐sensing supramolecular polymer reported by Han et al. operates through a selective host–guest interaction that requires the use of a specific macrocyclic supramolecular TADF system, and thus, this design cannot be easily generalized. Coumarins provide an attractive platform for the development of organic VOC sensors. They are synthetically accessible, inexpensive, and their properties are widely studied. When decorated with donor groups, these compounds possess emissive CT states typically with high PL quantum yields (Φ_PL_) [18, 22]. Our group has contributed to this field by introducing donor‐decorated coumarin derivatives and demonstrating how small structural modifications can modulate their photophysical behavior [14, 18]. Building on this foundation, we designed four new D–A emitters in which an ester‐functionalized coumarin acts as the acceptor unit and nitrogen‐heterocyclic donors of increasing strength are introduced at the 7‐position, enabling systematic modulation of the CT character, the PL spectrum, and the magnitude of the Δ*E*_ST_ of these emitters (Figure 1a,b). Embedding these emitters in photocurable acrylate resins and fabricating the resulting materials by digital light processing (DLP) by 3D printing facilitates their homogeneous dispersion within robust, spatially defined polymer networks that can be exploited for VOC sensing. The use of these resin substrates provides robust sensing performance that is not accessible using conventional thin films or emitter deposition on paper substrates where the diffusion of VOC vapors is constrained by the planar geometry and limited structural control of the sensing layer [23, 24]. Unlike thin films, 3D‐printed objects allow precise control over geometry, thickness, and mechanical properties, offering tunable VOC permeability and the possibility of integrating multiple emitters within a single device. However, the use of emissive molecules within photocurable matrices specifically for VOC sensing remains limited [16, 17, 25]. Herein we introduce four TADF donor‐coumarin emitters that display reproducible, analyte‐dependent optical responses across a broad panel of industrially relevant VOCs when embedded in 3D‐printed materials (Figure 1c), making them a versatile platform for processable solid‐state VOC detection.

## 2 Results and Discussion

### 2.1 Computational Study

**FIGURE 2:**
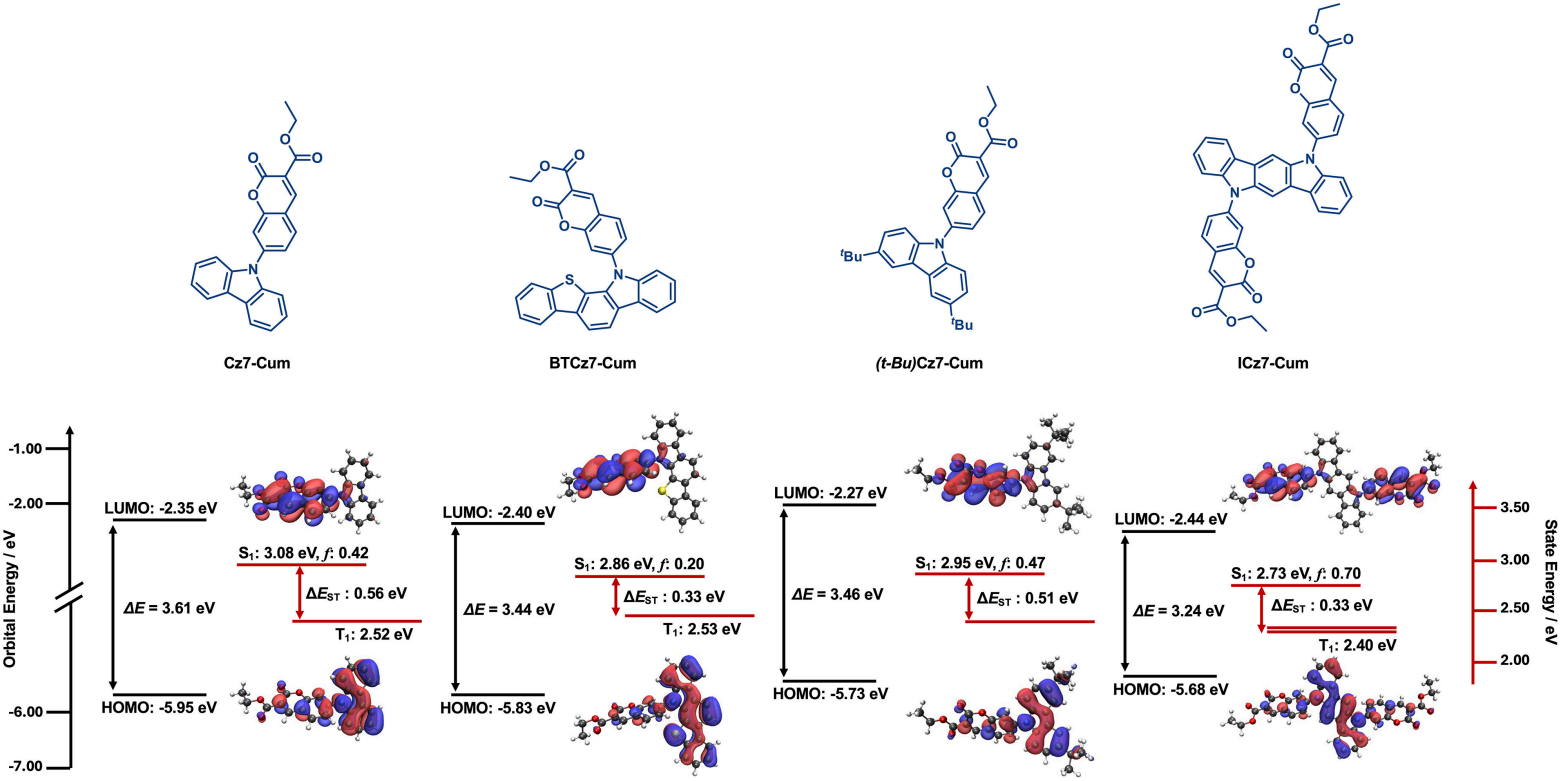
DFT‐calculated energies of the HOMO/LUMO and the S_1_ and T_1_ states of **Cz7‐Cum**, **BTCz7‐Cum**, **(*t*‐Bu)Cz7‐Cum**, and **ICz7‐Cum** and frontier molecular orbital electron density distributions at the PBE0/6‐31G(d, p) level of theory in the gas phase at the optimized S_0_ geometry (isovalue = 0.02).

Recently, we reported a series of carbazole‐coumarin derivatives exhibiting TADF, which were investigated for their potential as oxygen sensors by exploiting the emission quenching arising from the transient population of accessible triplet excited states [14]. Here, we demonstrate how structural analogs bearing donors of differing strength modulate the photophysical properties of these compounds (see the Supporting Information, Synthesis section for their synthesis. Single‐crystal X‐ray diffraction data for BTCz7‐Cum and (t‐Bu)Cz7‐Cum are provided in Figures S17–S21 and Tables S2–S7). Their molecular design was supported by density functional theory (DFT) calculations performed using the Digichem platform [26, 27]. The frontier molecular orbitals (FMOs) were modeled at the PBE0/6‐31G(d, p) level of theory in the gas phase. The electron density plots of the HOMO and LUMO are shown in Figure 2. DFT calculations predict the D‐A electronic structure of this series, with the HOMO is primarily localized on the heterocyclic donor in which the HOMO level is progressively destabilized with increasing donor strength (**Cz7‐Cum** < **BTCz7‐Cum** < **(*t*‐Bu)Cz7‐Cum** < **ICz7‐Cum**), while the LUMO level is much less affected as it is mainly localized on the ester‐functionalized coumarin. The strongly destabilized HOMO of **ICz7‐Cum**, in particular, is driven by the extended π‐conjugation of the indolocarbazole donor rather than donor strength alone, giving this derivative a distinct electronic profile. As a consequence, the S_1_ state is progressively stabilized across the series accompanied by a concomitant decrease in Δ*E*_ST_ (calculated at the TDA‐DFT‐PBE0/6‐31G(d, p) level [28]). The calculated oscillator strengths (*f*) for the S_0_–S_1_ transition lie in the 0.20–0.70 range, suggesting fast radiative decay rate constants, *k*_r_, and, by extension, high Φ_PL_. Natural transition orbital (NTO) analysis confirms CT‐dominated S_1_ states and mixed CT‐ locally‐excited (LE) character for the T_1_ states (Figures S3–S6). Moderate Δ*E*_ST_ values combined with finite spin–orbit coupling (SOC) between T_1_ and S_1_ (Table 1) are consistent with the experimentally observed variation in TADF efficiency. For **ICz7‐Cum**, a higher‐lying T_2_ state lies close in energy to S_1_ (Δ*E*_ST2_ = 0.29 eV); however, the S_1_→T_2_ SOC is extremely small (0.0023 cm^−1^), suggesting that T_2_ does not contribute significantly to reverse intersystem crossing (RISC), which is dominated by the T_1_→S_1_ channel in all four compounds. The computational data are summarized in Table 1.

**TABLE 1:** Summary of calculated data for Cz7‐Cum, BTCz7‐Cum, (*t*‐Bu)Cz7‐Cum, and ICz7‐Cum.

	**HOMO, eV** ^a^	**LUMO, eV** ^a^	**Δ*E***_**H‐L**_, **eV** ^a^ ^,^ ^b^	**S**_**1**_, **eV** ^**a**^	**T**_**1**_, **eV** ^**a**^	**Δ*E***_**ST**_, **eV** ^a,^, ^c^	***f*** ^a^ ^,^ ^d^	**SOC, cm** ^**−1**^ ^a^ ^,^ ^e^
**Cz7‐Cum**	−5.95	−2.35	3.61	3.08	2.52	0.56	0.42	0.47
**BTCz7‐Cum**	−5.83	−2.40	3.44	2.86	2.53	0.33	0.20	1.59
**(*t*‐Bu)Cz7‐Cum**	−5.73	−2.27	3.46	2.95	2.44	0.51	0.47	0.38
**ICz7‐Cum**	−5.68	−2.44	3.24	2.73	2.40	0.33	0.70	0.24

a Calculated at the PBE0/6‐31G(d, p) level of theory in the gas phase.

b Defined as the energy difference between the HOMO and LUMO levels.

c Defined as the energy difference between the S_1_ and T_1_.

d Refers to the S_0_–S_1_ electronic transition.

e Calculated at the optimized S_1_ geometry.

### 2.2 Optoelectronic Properties

**FIGURE 3:**
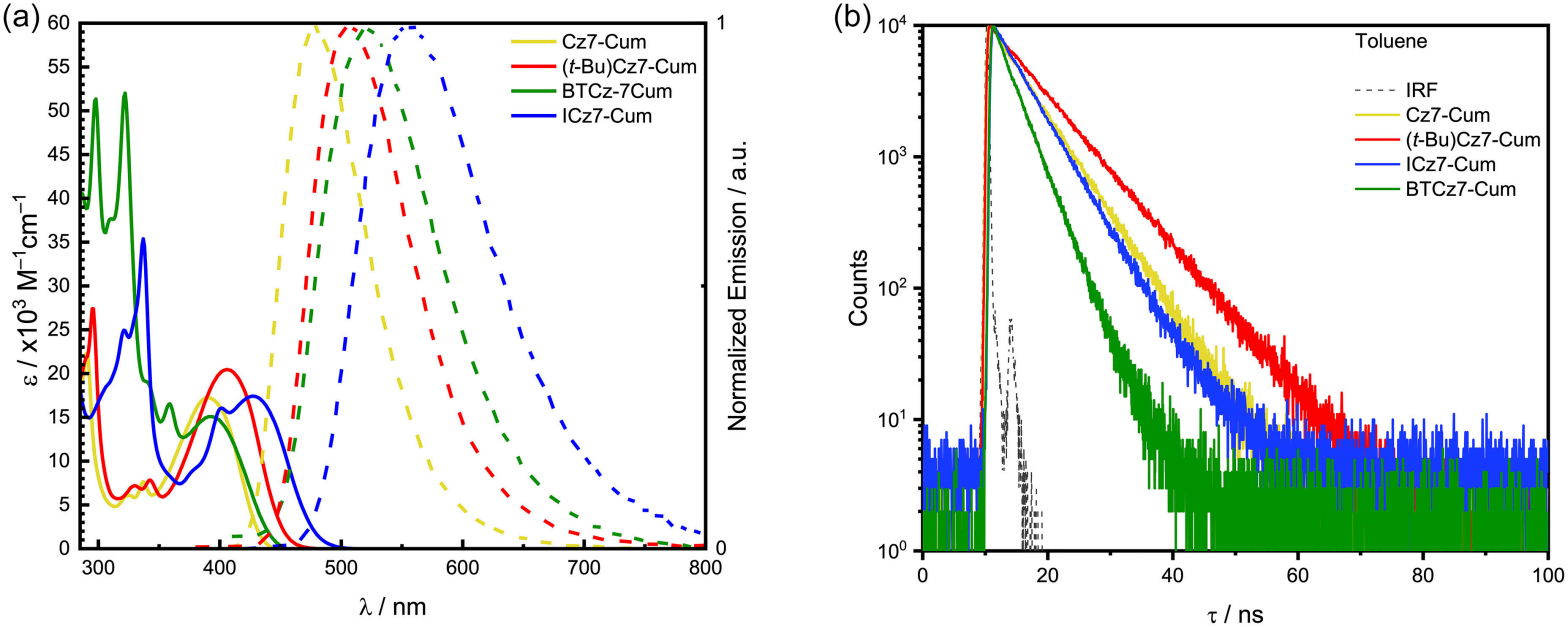
(a) Absorption (solid) and PL (dashed) spectra of the emitters in optically dilute toluene solutions (≈10^−5^ M). *λ*_exc_ = 360 nm (**Cz7‐Cum**, **(*t*‐Bu)Cz**)**7‐Cum)**, 320 nm (**BTCz7‐Cum**), and 400 nm (**7ICz7‐Cum**). (b) Time‐resolved PL decays of the emitters (**Cz7‐Cum** *λ*_PL_ = 477 nm, **(*t*‐Bu)Cz7‐Cum** *λ*_PL_ = 500 nm, **BTCz7‐Cum** *λ*_PL_ = 522 nm, and **ICz7‐Cum** *λ*_PL_ = 577 nm) in optically dilute solutions (≈10^−5^ M) recorded by time‐correlated single‐photon counting (TCSPC), *λ*_exc_ = 375 nm.

The trend in the oxidation and reduction potentials measured by electrochemistry in DCM qualitatively aligns with the DFT results, implying accurate assignment of the nature of the donor and acceptor moieties and the relative strength of the former in this series of compounds (Figure S22 and Table S8), with a gradual destabilization of the HOMO with the increasing donor strength. We next evaluated the photophysical properties of these four compounds in both the solution and the solid state. The UV–Vis absorption spectra of **Cz7‐Cum, (*t*‐Bu)Cz7‐Cum, BTCz7‐Cum,** and **ICz7‐Cum** (Figure 3a and Figure S23) all exhibit a broad, low‐energy band with λ_abs_ between 390 and 430 nm, consistent with a CT band. The corresponding molar absorptivity (ε) of this band is 1.7 × 10^4^ M^−1^ cm^−1^ (391 nm) for **Cz7‐Cum**, 2.0 × 10^4^ M^−1^ cm^−1^ (406 nm) for **(*t*‐Bu)Cz7‐Cum**, 1.5 × 10^4^ M^−1^ cm^−1^ (395 nm) for **BTCz7‐Cum**, and 1.7 × 10^4^ M^−1^ cm^−1^ (427 nm) for **ICz7‐Cum**. The onset of this CT band progressively redshifts across the series, in good qualitative agreement with the DFT‐calculated decrease in the HOMO–LUMO energy gap, which arises primarily from the gradual destabilization of the donor‐centered HOMO while the coumarin‐centered LUMO remains largely unchanged. A weaker, vibronically‐resolved absorption band at around 340 nm is also present across the series. While its energetic position remains largely unchanged, this band becomes significantly more intense for emitters bearing more π‐extended donors, being most pronounced in the spectrum of **ICz7‐Cum**. This behavior is consistent with this band being locally excited on the donor moiety, as supported by TDA‐DFT calculations of the singlet excited state (Table S1 and Figures S1,S2). The intense high‐energy π–π* transition of the coumarin core between 300–322 nm remains essentially unchanged.

The PL spectra in dilute toluene solution (Figure 3a, dashed lines, and Figure S24) are consistent with the expected CT emission, with peak maxima, *λ*_PL_, generally shifting bathochromically across the series; however, deviations from the expected donor‐strength trend are observed. In particular, **BTCz7‐Cum** shows a more redshifted emission than **(*t*‐Bu)Cz7‐Cum**, which is attributed to the more extended π‐conjugation and electronic delocalization of the BTCz donor relative to (*t*‐Bu)Cz. Likewise, the PL spectra of all four emitters exhibit clear positive solvatochromism, indicating that the emission is predominantly of CT character (Figure S25). The observed structured emission for **ICz7‐Cum** in polar solvents reflects a mixed LE/CT emissive excited state, rather than a purely CT emission. A summary of *λ*_abs_ and *λ*_PL_ values in solution is provided in Table S9. Steady‐state (SS) PL and phosphorescence measurements in frozen 2‐methyltetrahydrofuran (2‐MeTHF) glass at 77 K (Figure S26) show CT‐like S_1_ emission and vibronically structured LE T_1_ phosphorescence. Together with the T_1_ NTOs (Figures S3–S6), these data indicate that the LE component of the triplet is mainly coumarin‐centered. Experimental S_1_/T_1_ energies, obtained from the onsets of these spectra in 2‐MeTHF glass at 77 K, result in Δ*E*_ST_ values (see Table S9) whose trend correlates strongly with the TDA‐DFT predictions (Table 1). While the Δ*E*_*ST*_ value for **BTCz7‐Cum** closely matches that predicted computationally, the measured Δ*E*_*ST*_ values for **Cz7‐Cum**, **(*t*‐Bu)Cz7‐Cum**, and **ICz7‐Cum** (0.38, 0.35, and 0.13 eV, respectively) are much smaller than those predicted in the gas phase (0.56, 0.51 and 0.33 eV, respectively), due to the greater stabilization of the S_1_ state under experimental conditions. Time‐resolved PL (TRPL) measurements in ≈10^−5^ M degassed toluene solution (Figure 3b) show fast decay kinetics, with prompt PL lifetimes, *τ*_p_, of 5.8, 7.5, 3.3, and 5.2 ns for **Cz7‐Cum**, **(*t*‐Bu)Cz7‐Cum**, **BTCz7‐Cum**, and **ICz7‐Cum**, respectively. The Φ_PL_ values in aerated toluene are 52% for **Cz7‐Cum**, 55% for **(*t*‐Bu)Cz7‐Cum**, 31% for **BTCz7‐Cum**and 25% for **ICz7‐Cum**. No delayed emission was observed in toluene.

**FIGURE 4:**
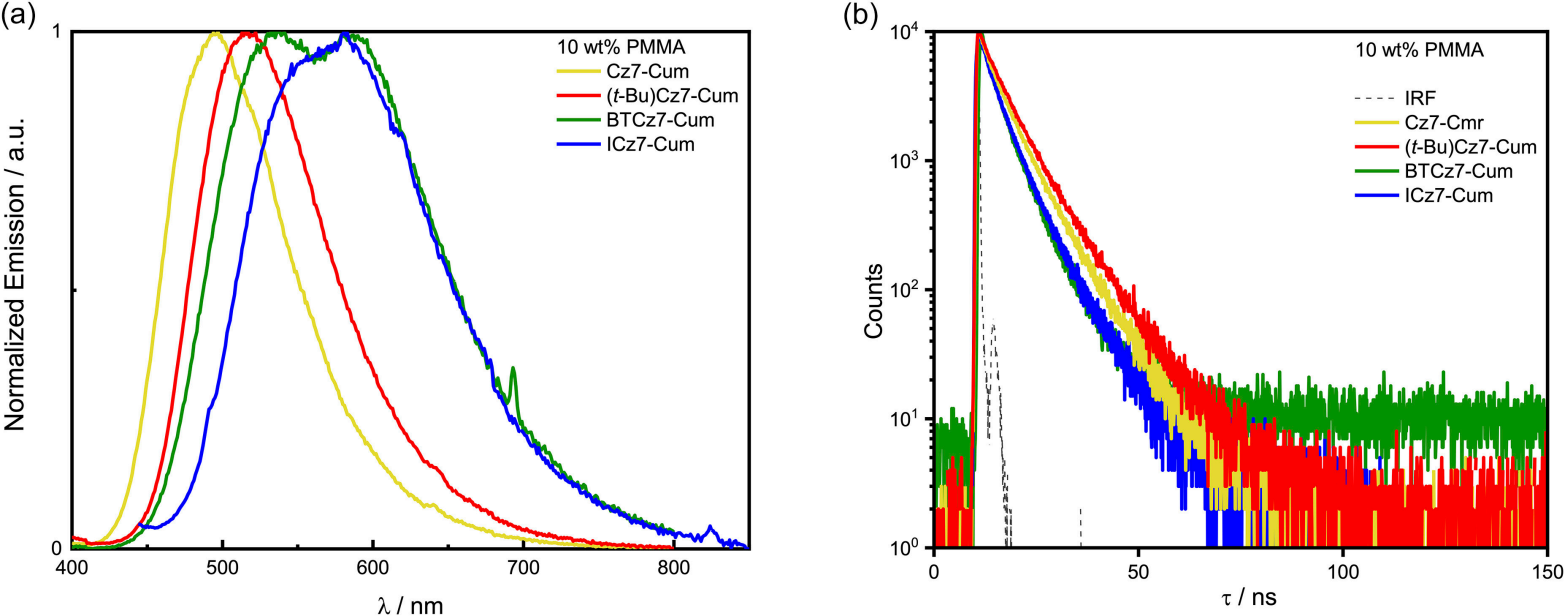
(a) Normalized PL spectra of the 10 wt% doped films of emitters in PMMA. *λ*_exc_ = 360 nm (**Cz7‐Cum, (*t*‐Bu)Cz)7‐Cum**), 320 nm (**BTCz7‐Cum**), 400 nm (**ICz7‐Cum**). (b) Time‐resolved PL decays of the prompt emission of the 10 wt% doped films of the emitters in PMMA measured by TCSPC, *λ*_exc_ = 375 nm.

We next examined the photophysical properties of the emitters doped in poly(methyl methacrylate) (PMMA) films. PMMA was chosen as an inert host model to evaluate the intrinsic photophysical properties of the emitters in the solid state prior to their introduction within the photopolymerizable matrix. A 10 wt% loading was selected to ensure sufficient emitter density to observe aggregation and delayed fluorescence effects in a rigid polymeric environment, while maintaining homogeneous films suitable for optical characterization. All derivatives exhibit broad and structureless emission as 10 wt% doped into PMMA films which is redshifted compared to that in toluene (Figure 4a and Figure S27). This behavior reflects the tendency of the emitters to aggregate at this concentration in the polymer matrix. Under N_2_, **Cz7‐Cum** and **(*t*‐Bu)Cz7‐Cum** retain high Φ_PL_, whereas **BTCz7‐Cum** and **ICz7‐Cum** emit less efficiently (Table 2), consistent with the relative LE and CT character to the emissive excited state of these compounds. At 77 K, all four films show structureless S_1_ fluorescence and vibronically‐structured T_1_ phosphorescence (Figure S28), confirming a CT‐dominated singlet state and more LE‐like triplet state at this temperature. Compared to measurements in 2‐MeTHF glass at 77 K, the PMMA films show systematically smaller Δ*E*_ST_ values across the series. This decrease arises from host‐dependent shifts of the experimentally estimated S_1_ and T_1_ onset energies. In particular, the S_1_ energy is generally lower in the PMMA films compared to measurements in 2‐MeTHF glass, while the T_1_ energy is less affected, except for **ICz7‐Cum** for which a slight increase in T_1_ is observed. Importantly, the relative trend in Δ*E*_ST_ across the series is preserved. The Δ*E*_ST_ values obtained in 2‐MeTHF glass (Table S9) and in PMMA films (Table 2) were determined under different experimental conditions and serve distinct purposes. The 2‐MeTHF measurements were used to assess the intrinsic molecular excited‐state energetics of the emitters in a rigid dilute medium, whereas the PMMA film measurements provide complementary information on their behavior in a solid‐state polymeric host. Therefore, the two datasets are discussed in terms of qualitative trends rather than by direct quantitative comparison.

**TABLE 2:** Photophysical properties of coumarin derivatives Cz7‐Cum, BTCz7‐Cum, (*t*‐Bu)Cz7‐Cum, and ICz7‐Cum as 10 wt% doped film in PMMA.

	***λ***_**PL**_, **nm** ^**a**^	***τ***_**p, avg**_, **ns** ^**b**^	***τ***_**d, avg**_, **μs** ^**c**^	**Φ**_**PL**_, **% N**_**2**_ a ^**,**^ ^d^	**Φ**_**PL**_, **% air** ^**a**^ ^**,**^ ^**d**^	**S**_**1**_ **/T**_**1**_, **eV** ^**e**^	**Δ*E***_**ST**_ **,** **eV** ^**f**^
**Cz7‐Cum**	495	6.2	1990.1	62	60	2.82/2.48	0.34
**(*t*‐Bu)Cz7‐Cum**	518	7.0	1517.0	69	60	2.74/2.47	0.27
**BTCz7‐Cum**	563	4.9	185.7	22	18	2.72/2.47	0.25
**ICz7‐Cum**	575	5.3	61.0	22	19	2.55/2.51	0.04

a Spin‐coated 10 wt% thin films in PMMA. *λ*_exc_ = 360 nm (**Cz7‐Cum** and **(*t*‐Bu)Cz)7‐Cum**, 320 nm (**BTCz7‐Cum**) and 400 nm (**ICz7‐Cum**).

b Prompt lifetime was measured by TCSPC. *λ*_exc_ = 375 nm.

c Delayed lifetime was measured by MCS. *λ*_exc_ = 360 nm (**Cz7‐Cum** and (**(*t*‐Bu)Cz)7‐Cum**), 320 nm (**BTCz7‐Cum**) and 400 nm (**ICz7‐Cum**).

d Determined using an integrating sphere.

e At 77 K. *λ*_exc_ = 360 nm (**Cz7‐Cum** and **(*t*‐Bu)Cz7‐Cum**), 320 nm (**BTCz7‐Cum**) and 400 nm (**ICz7‐Cum**).

f Determined from the onsets of the SSPL and delayed emission spectra at 77 K.

TRPL measurements of the films (Figure 4b) show multiexponential decay kinetics with *τ*_p,avg_ in the 4–7 ns range and delayed fluorescence on the tens‐to‐thousands of microseconds timescale, whose lifetime, *τ*_d, avg_, is proportional to the magnitude of the Δ*E*_ST_.

**FIGURE 5:**
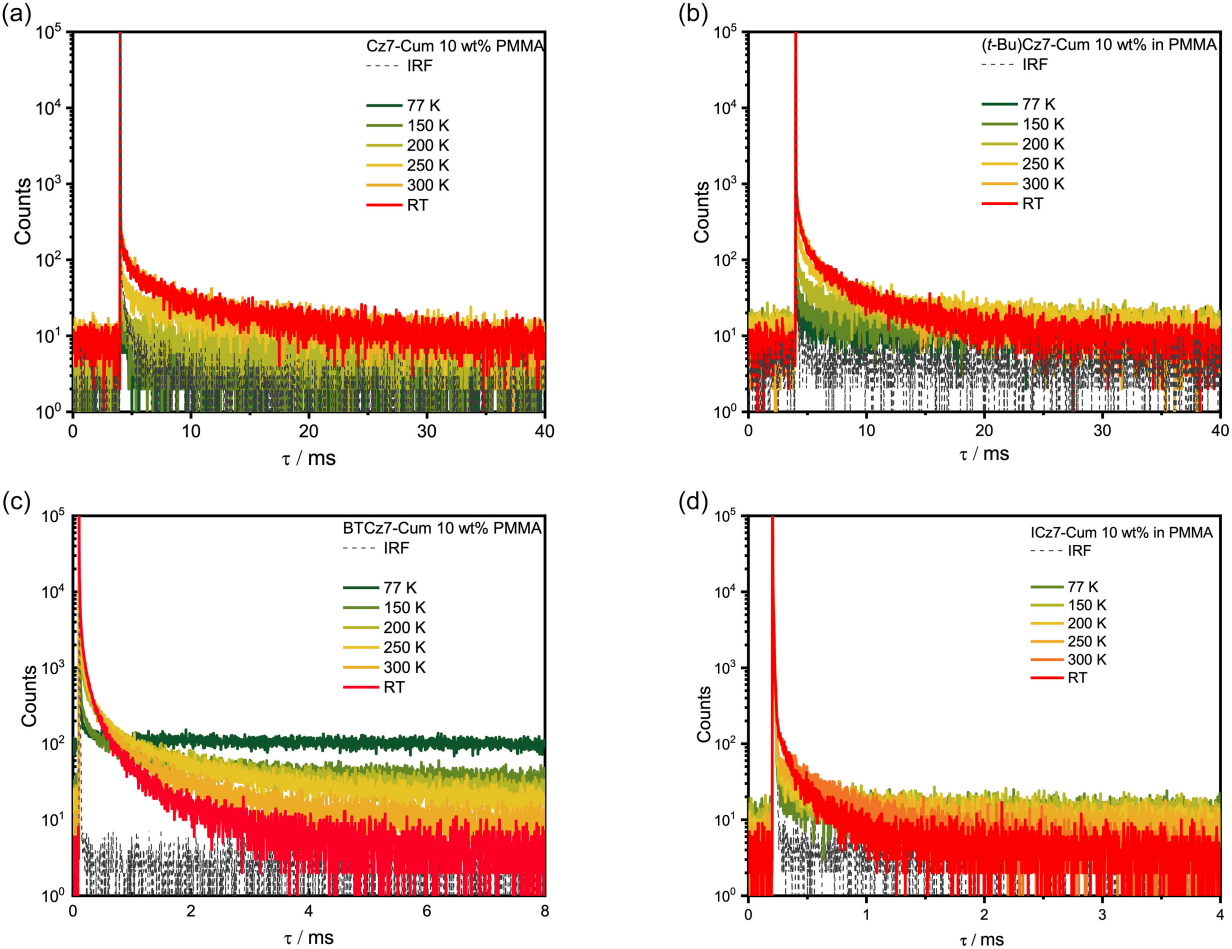
Time‐resolved PL decays of the delayed emission of (a) **Cz7‐Cum** (top left, *λ*_exc_ = 360 nm, *λ*_PL_ = 495 nm), (b) **(*t*‐Bu)Cz7‐Cum** (top right, *λ*_exc_ = 360 nm, *λ*_PL_ = 518 nm), (c) **BTCz7‐Cum** (bottom left, *λ*_exc_ = 320 nm, *λ*_PL_ = 563 nm), and (d) **ICz7‐Cum** (bottom right, *λ*_exc_ = 400 nm, *λ*_PL_ = 575 nm) in 10 wt% doped films in PMMA, measured by MCS.

The temperature dependence of the intensity of the delayed emission confirms that these compounds are TADF (Figure 5). **BTCz7‐Cum** shows a pronounced delayed emission component, consistent with its relatively small Δ*E*_*ST*_ and large calculated SOC, while **ICz7‐Cum** exhibits the shortest *τ*_d_, in line with its very small Δ*E*_*ST*_. Overall, the solid‐state photophysics in PMMA establish that all four compounds emit from CT‐like states showing TADF in this matrix, a prerequisite for their use as VOC sensors in photocurable matrices, as access to low‐lying triplet states enhances the sensitivity of the emission intensity to microenvironmental changes within the polymer host.

### 2.3 Fabrication of 3D‐Printed VOC‐Responsive Polymer Materials

**FIGURE 6:**
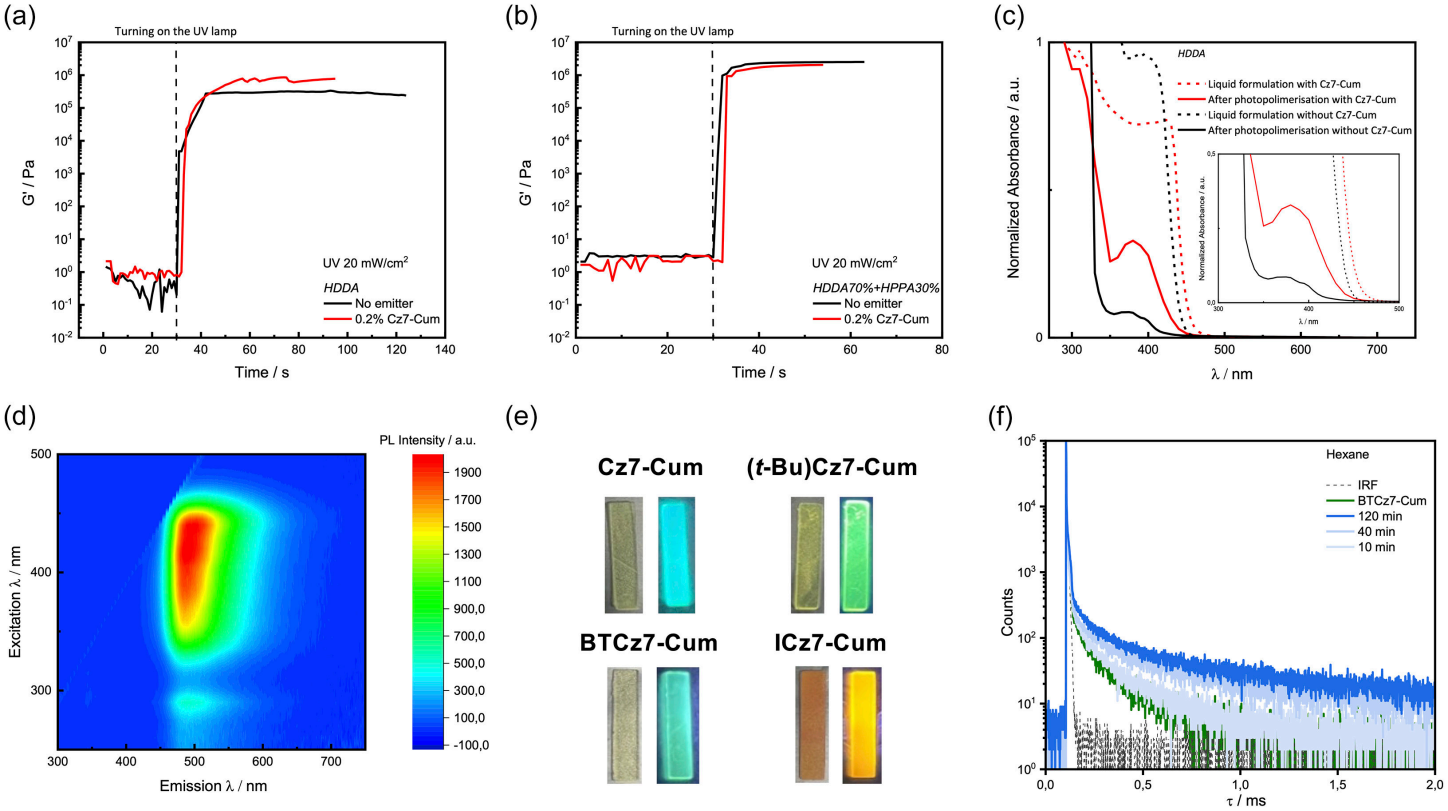
(a) Storage modulus (G′) as a function of irradiation time for HDDA formulations containing 0.2 wt% **Cz7‐Cum**. Film thickness 100 μm, irradiation power 20 mW. (b) Storage modulus, Gʹ as a function of irradiation time for HDDA/HPPA (70/30 wt%) formulations containing 0.2 wt% **Cz7‐Cum**. Film thickness 300 μm, irradiation power 20 mW. (c) UV–Vis absorption before (dashed line) and after (solid line) photopolymerization, without (black) and with (red) 0.2 wt% **Cz7‐Cum** in HDDA. (d) Excitation‐emission map of 3D‐printed 0.2 wt% **Cz7‐Cum** in HDDA. (e) Digital photographs of 3D‐printed HDDA70%‐HPPA30% samples containing **Cz7‐Cum, BTCz7‐Cum, (*t*‐Bu)Cz7‐Cum**, and **ICz7‐Cum** under natural daylight (left) and 365 nm UV excitation (on the right). (f) Time‐resolved decays of the delayed emission of **BTCz7‐Cum** (*λ*_exc_ = 390 nm, *λ*_em_ = 510 nm) in 2 wt% 3D‐printed material in HDDA/HPPA (70/30 wt%) upon exposure to hexane, collected by multichannel scaling (MCS).

To translate the photophysical behavior observed in PMMA, used here as a reference host rather than a sensing platform, into a functional material for vapor sensing, the emitters must be embedded in a photocurable matrix compatible with 3D fabrication and able to support efficient emission together with adequate VOC uptake. The Δ*E*_ST_ values were therefore determined in PMMA as a controlled solid‐state reference host, whereas a selected photocurable resin was used to evaluate the intensity‐based sensing response in a printable matrix. The dye loading used in PMMA (10 wt%) cannot be applied to photocurable acrylate resins because the emitters absorb light in the 390–430 nm region in solution (Figure 3a), partially overlapping with absorption by the photoinitiator phenylbis(2,4,6‐trimethylbenzoyl)phosphine oxide (BAPO) (Figure S30). At high loading, the emitters competitively absorb the light from the excitation beam to the point that photopolymerization slows dramatically, as confirmed by photorheology experiments (Figures S31–S34). A lower concentration of 0.2 wt% provides the best compromise between limiting competitive absorption of the 405 nm curing light by the dye (preserving efficient photopolymerization in the presence of BAPO and improving printing resolution) and maintaining sufficient emission intensity for reliable photophysical characterization of the embedded emitters. For the screening of commercial acrylate resins, **Cz7‐Cum** was chosen as a representative emitter since all four compounds emit from comparable CT‐type states and show TADF in PMMA, suggesting similar compatibility with acrylate matrices at low concentration. At the lower dye loading used for printing (0.2 wt%), the emitter retains the broad CT‐type emission observed in 10 wt% PMMA film and in solution, with only minor variations in *λ*_PL_ depending on the host matrix (Table 3). The screening focused on identifying matrices that produce a material in which the emitter is homogeneously dispersed, maintains a sufficiently high Φ_PL_ under air to be used for sensing and can undergo efficient photocuring under 405 nm irradiation. Despite the different doping concentrations between PMMA and the commercial acrylates, the CT emission features and the relatively high Φ_PL_ trends measured for the PMMA films were conserved across the acrylate matrices (further formulation details are reported in the Supporting Information, Figures S35–S38). Five commercially available acrylate oligomers were selected to obtain polymer materials covering a range of rigidity and polarity characteristics (Figure S29): a silicon‐acrylate (Tegorad), an acrylic resin containing bisphenol A (BEDA), polyethylene glycol diacrylate monomers of two different molecular weights (PEGDA 250 and PEGDA 575), and 1,6‐hexanediol diacrylate (HDDA). Photocurable resins were prepared by dispersing **Cz7‐Cum** at low loading in acrylate‐based monomer mixtures and homogenizing the mixtures by sonication, followed by addition of BAPO. Details on resin preparation, dye dispersion, and curing conditions are provided in the Supporting Information (Tables S11,S12). **Cz7‐Cum** was physically dispersed into the resins and showed good miscibility with all matrices, except for Tegorad, which required acetone as a cosolvent to achieve complete dissolution of the BAPO photoinitiator (Table S11). Acetone was added in the minimum amount required to obtain a clear solution; the solvent acts only as a transient processing aid and does not affect the cured material. In the liquid formulation, the strong absorption (ca. *λ*_abs_ of 450 nm) from the BAPO photoinitiator disappeared upon photopolymerization, confirming its full consumption. The CT absorption band of **Cz7‐Cum**, centered at around 400 nm, remained post‐polymerization, indicating that the emitter did not photochemically degrade during the photocuring. There were small differences in photophysical behavior between the five emitter‐doped resins, reflecting differing microenvironments about the emitter. The excitation wavelength was adjusted to match the absorption profile of each formulation. The *λ*_PL_ modestly changed across the series of five resins (Table 3), where the most redshifted emission in PEGDA 575 was attributed to its higher polarity, whereas the blueshift in Tegorad arises from the lower polarity of the silicon‐acrylate matrix. The Φ_PL_ values ranged from 33% to 63%, mirroring the network rigidity of the host resin (Tegorad < PEGDA 575 < BEDA < PEGDA 250 < HDDA). HDDA provides the highest Φ_PL_ and preserves the PL profile most closely to that observed in the PMMA reference films, while still maintaining curing efficiency under 405 nm irradiation. BEDA and PEGDA 250 showed acceptable performance, whereas the PEGDA 575 and Tegorad exhibited significantly lower Φ_PL_. Considering the preservation of the CT‐type emissive character, the formation of homogeneous formulations and photocuring performance, HDDA was identified as the most suitable matrix among the five assessed for further optimization (Figure 6a–d). Subsequent adjustments to the matrix composition, described below, were introduced to balance mechanical robustness with VOC permeability.

**TABLE 3:** Photophysical properties of Cz7‐Cum in the polymeric resins investigated.

**Resin type** ^**a**^	**Absorbance, %** ^**b**^	**Φ**_**PL**_, **%**	***λ***_**PL**_ **,** **nm**
Pegda 250	63.1	54	510
Pegda 575	58.3	40	525
Beda	60.8	50	500
Tegorad	67.6	33	485
HDDA	53.2	63	500

a All formulations included 1 wt% BAPO as the photoinitiator and 0.2 wt% of **Cz7‐Cum**, selected to ensure sufficient photophysical performance while maintaining 3D printability.

b % Absorbance refers to the internal absorbance factor calculated using an integrating sphere during Φ_PL_ measurements and does not represent a separately measured absorbance value. It is reported only as an instrument‐derived parameter.

Photorheological measurements were then employed to evaluate the processability of the dye‐loaded formulations and to identify dye concentrations compatible with DLP 3D printing. After establishing suitable curing conditions using **Cz7‐Cum** as a representative emitter, the analysis was extended to the other donor‐coumarin derivatives in this study to assess how dye dispersion and molecular structure influence the maximum printable concentration. All measurements were performed under UV irradiation. Increasing dye loading led to a progressive delay in photopolymerization due to competitive absorption of the curing light, whereas at low loading (0.2 wt% or 0.1 wt% for **ICz7‐Cum**), curing proceeded rapidly and reproducibly. Detailed photorheological data are provided in the Supporting Information (Figures S31–S33). As highly crosslinked acrylates such as HDDA can hinder VOC diffusion [29, 30], HPPA was introduced as a monoacrylated monomer to moderate the crosslink density and slightly increase matrix polarity without altering the photophysical behavior of the embedded emitters. Photorheological studies of the HDDA/HPPA (70/30 wt%) formulation showed curing profiles comparable to neat HDDA (Figure S34), indicating that mechanical integrity was preserved while analyte diffusion was enhanced. Based on these results, the HDDA/HPPA blend was selected as the printable matrix for subsequent studies, and all 3D‐printed sensing materials were fabricated at the optimized dye loading of 0.2 wt% (0.1 wt% for **ICz7‐Cum**) (Figure 6e).

### 2.4 3D‐Printed Coumarin‐Based Polymeric Materials for VOC Detection

**FIGURE 7:**
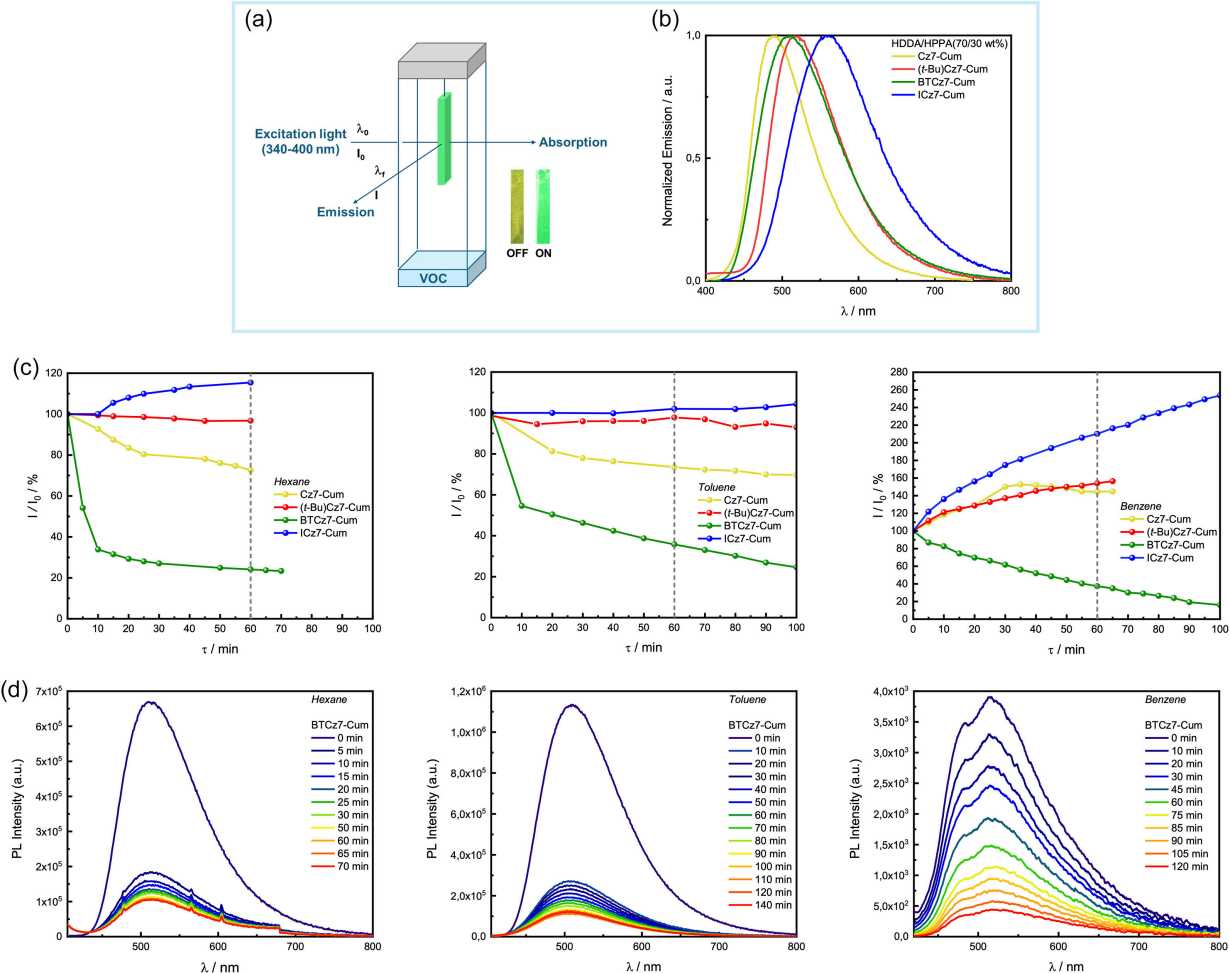
(a) Representation of the set‐up used for the sensing analysis. (b) The normalized emission spectra of the emitters in HDDA/HPPA (70/30 wt%) 3D‐printed materials. *λ*_exc_ = 360 nm (**Cz7‐Cum**), 340 (***t*‐Bu)Cz7‐Cum**), 390 nm (**BTCz7‐Cum**), and 400 nm (**ICz7‐Cum**). (c) Time‐dependent PL intensity of the sensor material composed of 70% HDDA and 30% HPPA containing 0.2 wt% **BTCz7‐Cum** (*λ*_exc_ = 390 nm), expressed as a percentage relative to its initial value (100%), upon exposure to hexane, toluene, and benzene vapor, respectively (see Figure S46 for the sensing experiments with complete set of VOCs) . (d) PL spectra of a film composed of 70% HDDA and 30% HPPA containing 0.2 wt% **BTCz7‐Cum** (*λ*_exc_ = 390 nm), recorded over time during exposure to hexane, toluene and benzene vapor, respectively. Measurements were carried out under identical conditions (see Figures S41–S44 for the complete fluorometric analysis using each of **Cz7‐Cum, BTCz7‐Cum, (*t*‐Bu)Cz7‐Cum**, and **ICz7‐Cum** with the full panel of VOCs).

After 3D printing the HDDA/HPPA (70/30 wt%) composites (see Table S12), all four coumarin‐based sensor materials were evaluated for their response to a broad panel of industrially relevant VOCs including aliphatic hydrocarbons (hexane), aromatic hydrocarbons (toluene), chlorinated solvents (dichloromethane), and oxygenated VOCs, such as aprotic (THF) and protic (methanol) solvents. These analytes cover a broad range of polarities and vapor pressures (Table S13), enabling an initial assessment of sensing performance across chemically diverse vapors. Each emitter was therefore evaluated individually across the same VOC panel to assess how molecular structure influences the sensing response. Sensing analyses were performed using a custom‐built setup (Figure 7a) at 25 °C (See Figures S41–S63 and Tables S14–S26 in the Supporting Information for a complete account of the sensing experiments). The PL spectra of **Cz7‐Cum, (*t*‐Bu)Cz7‐Cum, BTCz7‐Cum**, and **ICz7‐Cum** embedded in HDDA/HPPA (70/30 wt%) are shown in Figure 7b. The emission remains broad and structureless, with *λ*_PL_ of 490, 518, 510, and 562 nm, respectively. At the lower dye loading used in HDDA/HPPA (70/30 wt%), the emission maxima observed in the acrylate matrices remain within the range observed for the corresponding 10 wt% doped films in PMMA and in toluene solution. The modest shifts observed across these environments reflect differences in polarity and matrix rigidity rather than changes in the nature of the emissive state. This confirms that the CT‐type emission character of all four emitters is preserved in the printable formulations. In this context, TADF is not used as the sensing readout, but coincides with the CT nature of the excited state, which governs the observed VOC response.

The sensing behavior of the 3D‐printed materials was investigated by combining SS PL measurements with time‐dependent monitoring of the emission intensity under continuous VOC exposure, as illustrated in Figure 7c,d. The sensing response was monitored over time, with PL intensities expressed as I/*I*_0_ (%) and evaluated at selected exposure times ranging from 1 to 2 h depending on the nature of the VOC, where *I*_0_ corresponds to the initial emission intensity prior to vapor exposure. Upon exposure to VOCs, no change in spectral profile was observed, indicating that the response is intensity‐based across the full VOC panel and that no new emissive species are formed. When exposed to nonpolar vapors (hexane and toluene), **Cz7‐Cum** showed moderate PL quenching, retaining approximately 70% of its initial intensity. In contrast, **(*t*‐Bu)Cz7‐Cum**, despite its structural similarity, remained essentially unchanged (>90% retention), possibly because the *tert*‐butyl substituents sterically limit close VOC‐emitter interactions implying lower sensitivity of this material to nonpolar vapors. The PL intensity of **ICz7‐Cum** increases slightly (≥10%), while **BTCz7‐Cum** displays the most pronounced quenching response, retaining 24% (hexane) and 34% (toluene) of its initial emission. In the presence of THF, the PL intensity of **ICz7‐Cum** and **Cz7‐Cum** were minimally affected (ca. 90%). **(*t*‐Bu)Cz7‐Cum** showed a moderate response, retaining about 75% of its emission. **BTCz7‐Cum** again exhibited a stronger emission quenching, retaining roughly 60% of its emission after 1 h and 40% after 2 h of exposure. Exposure to MeOH vapors led to significant PL quenching for all four materials. **Cum‐7ICz** showed the largest decrease (retaining ca. 40%), followed by **BTCz7‐Cum** (ca. 50%), **(*t*‐Bu)Cz7‐Cum** (ca. 60%), and **Cz7‐Cum** (ca. 65%). These trends illustrate that each emitter exhibits a distinct response profile across the same VOC panel. DCM was excluded from detailed analysis because exposure caused softening and partial degradation of the printed composites (Figure S45), preventing reliable measurements. The absence of spectral shifts indicates that VOC exposure does not significantly perturb the energy of the emissive CT state, but instead influences the competition between radiative and nonradiative decay channels within the crosslinked matrix in a manner that is difficult to correlate directly with emitter structure. Intensity changes therefore arise from VOC‐induced perturbations of the local polymer microenvironment surrounding the embedded emitters. The sensing response reflects a combination of VOC uptake, matrix softening, and local polarity changes, which modulate the balance between radiative and nonradiative decay pathways of the CT excited state. The quenching trend observed with methanol (**Cz7‐Cum** < **BTCz7‐Cum** < **(*t*‐Bu)Cz7‐Cum** < **ICz7‐Cum**) follows the same order as the increasing CT character across the series. In particular, emitters bearing more π‐extended donors have higher‐lying HOMO levels and more delocalized CT excited states, which are intrinsically more sensitive to polarity‐induced perturbations and therefore exhibit a stronger PL response to polar protic vapors. This behavior is consistent with an enhanced sensitivity of the excited‐state deactivation pathways to changes in the local environment within the polymer matrix.

No delayed emission was observed from the **Cz7‐Cum**, **(*t*‐Bu)Cz7‐Cum,** or **ICz7‐Cum** containing materials in air. The material with **BTCz7‐Cum** was the only one that retained a measurable long‐lived PL component (*τ*_d_ in the μs range), which is consistent with a combination of its highest SOC and small Δ*E*_*ST*_ compared to the other derivatives (as measured in PMMA). Upon VOC exposure, **BTCz7‐Cum** showed an overall fluorescence quenching; however, there was an increase in the delayed fluorescence lifetime in the presence of hexane (Figure 6f). This observation highlights that VOC vapors can affect prompt and long‐lived emission differently, as we and others have recently shown with other analytes [31, 32]. Among the analytes tested, hexane produced the largest effect in terms of both the magnitude of the PL quenching and the change in *τ*_d_. Taken together, these preliminary results reflect a PL intensity sensitivity that is influenced by the combination of the nature of the VOC, the choice of acrylate matrix and the intrinsic photophysics of the coumarin‐based emitters. The observed VOC‐dependent responses highlight that each analyte induces a distinct optical signature, which can be exploited to discriminate among different VOC vapors using the same coumarin‐based sensing platform.

**FIGURE 8:**
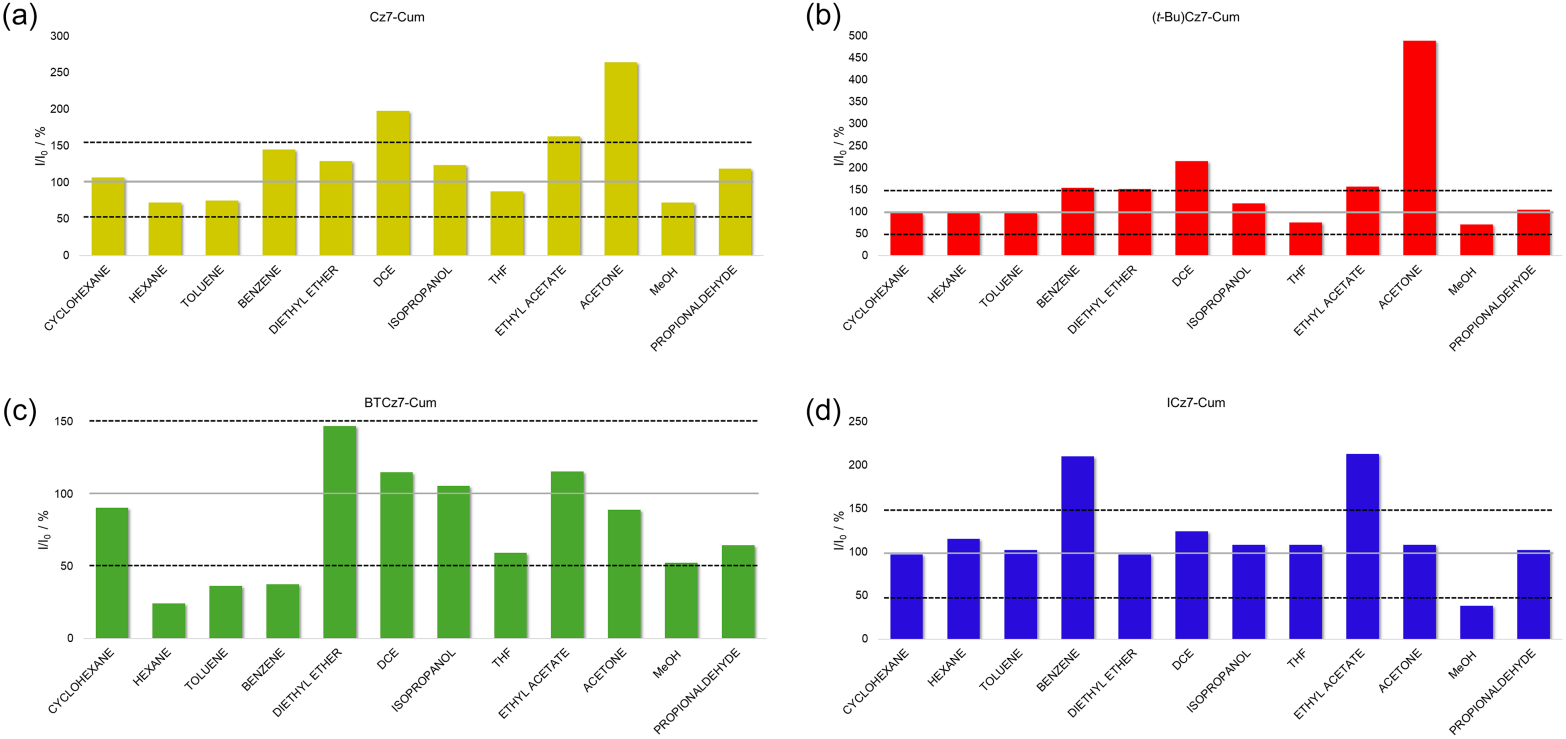
Bar charts representing the relative fluorescence intensity (I/I_0_, %) (a) of **Cz7‐Cum** (*λ*_exc_ = 360 nm), (b) **(t‐Bu)Cz7‐Cum** (*λ*_exc_ = 340 nm), (c) **BTCz7‐Cum** (*λ*_exc_ = 390 nm), (d) **ICz7‐Cum** (*λ*_exc_ = 400 nm), measured in the presence of different vapor solvents. I_0_ corresponds to the fluorescence intensity of the sensor material composed of 70% HDDA and 30% HPPA, containing the different emitters, used as the reference sensor material (100%). Reported values correspond to the emission intensity recorded 1 h after exposure. Values above or below 100% (solid line) indicate fluorescence enhancement or quenching, respectively. The dashed line at 50% indicates samples exhibiting fluorescence quenching greater than 50%, while the dashed line at 150% indicates samples exhibiting fluorescence enhancement greater than 150%.

The PL measurements performed with the initial set of VOCs show only modest variations, indicating that vapor exposure modulates the balance between radiative and nonradiative decay rates without altering the nature of the ICT emissive state. This interpretation is consistent with the absence of any spectral shifts throughout the full series of measurements. To extend the analysis to a broader class of VOCs, sensing experiments were performed following the same protocol (vide supra). The PL of **Cz7‐Cz** and **(*t*‐Bu)Cz7‐Cum** show marked intensity enhancement when exposed to 1,2‐dichloroethane (DCE) and acetone, exceeding 200% of their initial intensities (Figures S41f,l and S42f,l). Their similarity in response profiles is consistent with their closely related electronic structures and CT character. **BTCz7‐Cum**, by contrast, showed significant PL quenching in the presence of the nonpolar aromatic and aliphatic vapors of benzene, toluene, and hexane (Figure 7c,d). Interestingly, cyclohexane produced almost no change in the PL for any of the emitters. This comparison indicates that the sensing response reflects the combined influence of vapor pressure, molecular structure, and analyte‐matrix interactions. Within this multifactorial framework, the reduced response to cyclohexane may partly reflect its lower vapor pressure and limited uptake into the printed matrix. In this context, differences in molecular shape and flexibility can be relevant, as the linear hexane chain may more easily penetrate and interact with the embedded emitter than cyclic hydrocarbons [33]. Among VOCs containing oxygen atoms, exposure of **BTCz7‐Cum** to diethyl ether produced a PL enhancement of around 150%. A different behavior was observed for **ICz7‐Cum**, which showed strong PL enhancement in the presence of benzene and ethyl acetate vapors (ca. 100% increase in PL intensity) (Figure S44d,i), while MeOH was the only solvent that induced intense PL quenching in this material (Figure S44m). Interestingly, isopropanol, although also a polar protic VOC, produced no detectable change in PL intensity, consistent with its substantially lower vapor pressure (44 hPa) and thus poorer diffusion efficiency into the resin network. The opposite PL responses from **BTCz7‐Cum** (quenching) and **ICz7‐Cum** (enhancement) upon exposure to benzene highlight that sensing is not governed by a single physical parameter such as VOC polarity or vapor pressure. Instead, the observed behavior arises from the combined influence of VOC uptake efficiency, matrix‐VOC interactions (possibly π–π interactions between benzene and the phenyl group of HPPA) and the intrinsic photophysical properties of the emitters, each contributing to a distinct microenvironmental perturbation within the rigid HDDA/HPPA matrix that differently affects *k*_r_ and *k*_nr_ of the materials while remarkably not impacting the energy of the emissive excited state. A summary of these results is provided in Figure 8, with measurements for the most responsive VOCs repeated three times to verify outcome reproducibility (see Supporting Information, Repeatability section).

UV–Vis absorption and Φ_PL_ measurements were also conducted before and after vapor exposure to gain deeper insight into the photophysical processes underlying the observed changes in PL intensity (Figures S53–S63 and Tables S16–S26). UV–Vis absorption spectra recorded before and after VOC exposure were used to assess the stability of the emitters within the printed matrix. In most cases, vapor exposure caused only changes in the intensity of the absorption bands without altering their overall shape, and the solid‐state spectra remained closely comparable to those in solution, indicating that the VOCs do not induce chemical transformations of either the emitters or the matrix. Examples include **Cz7‐Cum** and **(*t*‐Bu)Cz7‐Cum** in DCE, **(*t*‐Bu)Cz7‐Cum** in acetone, **BTCz7‐Cum** in toluene and diethyl ether, and **ICz7‐Cum** in benzene and ethyl acetate (Figures S54–S56, S58, S60–S62). In the other cases, small variations in the fine structure of the high‐energy band (305–360 nm) were observed. These changes in the absorption spectra are VOC‐ and matrix‐dependent and likely do not indicate the formation of new absorbing species. A weak additional feature appeared only in the absorption spectrum of **ICz7‐Cum** upon exposure to methanol. However, this did not correspond to a new emissive species and the CT‐type PL remained unchanged. TD‐DFT calculations assign the absorptions in the 300–330 nm region to a LE π–π* transition of the coumarin acceptor, supporting the hypothesis that these small variations arise from microenvironmental effects rather than chromophore modification.

**FIGURE 9:**
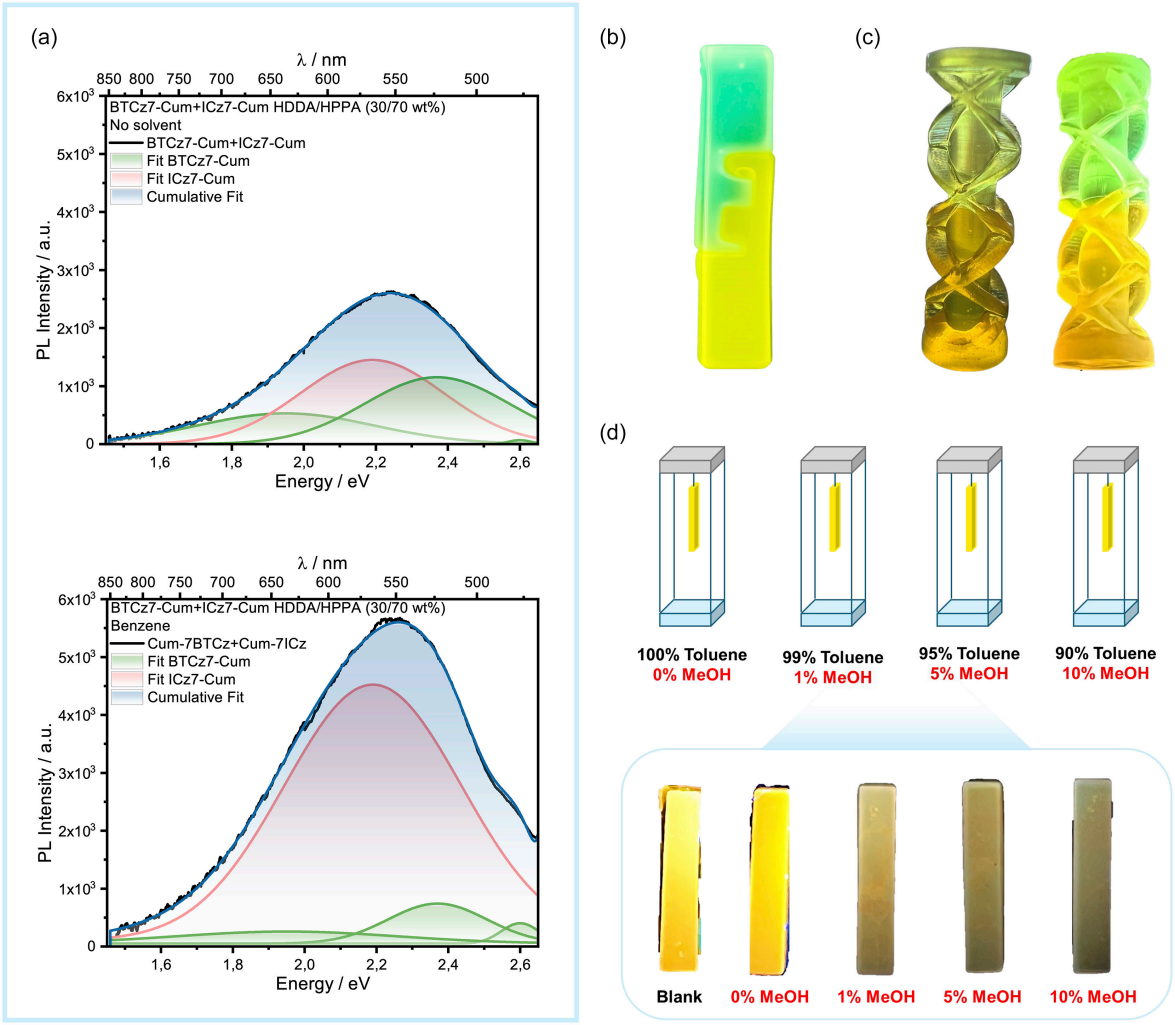
(a) SS PL spectra (black solid line) of the dual‐emitter 3D‐printed sensor containing **BTCz7‐Cum** and **ICz7‐Cum** before (top) and after (bottom) exposure to benzene vapors. The green and red curves correspond to the deconvoluted contributions of **BTCz7‐Cum** and **ICz7‐Cum**, respectively, while the blue shaded area represents the cumulative fit. *λ*_exc._ = 420 nm. (b) Photograph of the 3D‐printed dual‐emitter sensor under 365 nm UV excitation, showing the two distinct emissive regions embedded within the HDDA/HPPA polymeric matrix (70/30 wt%). (c) Photographs of a complex 3D‐printed object under natural daylight (left) and 365 nm UV excitation (right) containing **ICz7‐Cum** and **BTCz7‐Cum**. Both emitters preserve their solid‐state emission within a single object, indicating excellent printability and optical stability. (d) Specificity test of **ICz7‐Cum** under mixed toluene/MeOH vapors. **ICz7‐Cum** remains unresponsive to toluene alone, while 1%–10% v/v MeOH in toluene induces a progressive fluorescence quenching visible to the naked eye.

From these analyses, changes in PL intensity upon VOC exposure are accompanied by variations in the absorption spectra, reflecting modifications of the excited‐state deactivation pathways within the polymer matrix rather than a direct correlation with the Φ_PL_. To evaluate the response of the sensor in mixed‐vapor environments, **ICz7‐Cum** was exposed to vapors of a mixture of toluene and methanol. While toluene produced no PL response, the addition of 1%–10% v/v methanol in toluene resulted in clear and progressive PL quenching, visible to the naked eye (Figure 9d). These results show that an **ICz7‐Cum**‐based sensor can discriminate VOCs in mixtures and detect low concentrations of MeOH even in a strongly nonpolar background. Discrimination between VOCs in complex or mixed vapor environments has previously been explored using cross‐reactive optical sensor arrays and luminescent polymer‐based platforms, typically coupled with pattern‐recognition or multivariate analysis strategies [34, 37]. In contrast to these approaches, the behavior observed here arises from a direct optical response of a single molecular emitter embedded within a printable polymer matrix, without relying on sensor arrays or signal deconvolution. This highlights a simplified sensing paradigm in which microenvironmentally driven modulation of excited‐state deactivation pathways enables VOC discrimination.

We next provide a proof‐of‐concept sensor device that exploits the processability of these materials. We fabricated a dual‐emitter 3D‐printed sensor by combining two spatially separated regions doped with **BTCz7‐Cum** and **ICz7‐Cum** in an HDDA/HPPA (70/30 wt%) matrix (Figure 9b,c). Upon exposure to benzene vapor, the **BTCz7‐Cum** region exhibited PL quenching while the PL of the **ICz7‐Cum** region was simultaneously enhanced. These antagonistic PL responses were monitored during exposure to benzene and the resulting PL spectra were deconvoluted to follow the individual contributions of each emitter (Figure 9a), demonstrating that distinct optical signatures can be preserved and independently detected within a single printed object. To the best of our knowledge, examples in which two different molecular emitters embedded within the same printable polymer matrix exhibit opposite PL responses to the same VOC have not typically been reported in a single printed object using a direct optical readout. In this respect, the dual‐emitter response to benzene demonstrated here represents a distinct approach to VOC discrimination, arising from intrinsic differences in emitter photophysics rather than from sensor arrays or external pattern‐recognition schemes.

## 3 Conclusions

In this work, we designed and characterized a new family of donor‐substituted coumarins that exhibit TADF and whose PL is strongly sensitive to the environment. Embedded within photocurable matrices such as HDDA/HPPA, these molecules retain their ICT emission, and the resulting 3D‐printed materials displays reproducible, intensity‐based responses to industrially relevant VOCs. Each emitter shows a distinct sensing fingerprint across a broad VOC panel, enabling discrimination of chemically diverse vapors based solely on intensity response; notably, no spectral changes are observed as a result of interactions with the VOC vapors. Selectivity in mixed‐vapor environments and the successful fabrication of a dual‐emitter printed device further demonstrate the potential of this sensing platform.

## Author Contributions

**Sara Paniziutti:** investigation, software, validation, visualization, writing – original draft, writing – review and editing. **Maria Vittoria Piras:** investigation, validation. **Annalisa Chiappone:** investigation, resources, writing – original draft, writing – review and editing. **Enrico Podda:** investigation, writing – original draft. **Pier Carlo Ricci:** resources. **Stefania Porcu:** investigation. **Tomas Matulaitis:** investigation, methodology. **Eli Zysman‐Colman:** methodology, resources, software, supervision, visualization, writing – original draft, writing – review and editing. **Francesco Secci:** methodology, resources, supervision, visualization, writing – original draft, writing – review and editing.

## Funding

This work was supported by Engineering and Physical Sciences Research Council (Grants EP/R035164/1, EP/W007517/1, EP/Z535291/1), Royal Society of Chemistry (Grant C23‐0895140532), and European Commission (Grant P2022YM7F2).

## Conflicts of Interest

The authors declare no conflicts of interest.

## Supporting information

The authors have cited additional references within the Supporting Information [30, 31].

## Data Availability

The data that support the findings of this study are openly available in Pure at https://doi.org/10.17630/f37d9b12‐602c‐4fb8‐b137‐186035181f0f, reference number 331783246.
