# Supplementary material for: 3D‐Printed Optical Volatile Organic Compound Sensors Based on Donor‐Substituted Coumarin Thermally Activated Delayed Fluorescence Emitters
Source: Small Sci. 2026 Jun 21;6(6):e70325. doi: 10.1002/smsc.70325 (PMC13285140; doi:10.1002/smsc.70325)
Supplement: Supplementary file 1 — The authors have cited additional references within the Supporting Information [30, 31]. [file SMSC-6-e70325-s001.pdf]

3D-printed optical VOC sensors based on donor-substituted coumarin TADF emitters

*Sara Paniziutti,[a] [d], Maria Vittoria Piras,[a] Annalisa Chiappone,[a] Enrico Podda,[b] Pier Carlo Ricci,[c] Stefania Porcu,[c] Tomas Matulaitis,[d] Eli Zysman Colman, *[d] and Francesco Secci*[a]*

[a] Dipartimento di Scienze Chimiche e Geologiche, Università degli Studi di Cagliari, Complesso Universitario di Monserrato, blocco D. 09042, Monserrato (Ca), Italy. E-mail: fsecci@unica.it

[b] Centro Servizi di Ateneo per la Ricerca − CeSAR, Università degli Studi di Cagliari, Cittadella Universitaria, Monserrato − Cagliari 09042, Italy

[c]Dipartimento di Fisica, Università degli Studi di Cagliari, Complesso Universitario di Monserrato, 09042, Monserrato (Ca), Italy.

[d]Organic Semiconductor Centre, EaStCHEM School of Chemistry, University of St Andrews, St Andrews, UK, KY16 9ST. E-mail: eli.zysman-colman@st-andrews.ac.uk

SUPPORTING INFORMATION

Table of Contents

General Methods S2
Computational methodologies S7
Synthesis S10
^{1}H and ^{13}C NMR spectral characterization S15
X-Ray crystallography S20
Photophysical characterization S36
Polymers investigation S43
DLP 3D printing S52
Sensing analysis S55
References S96

General Methods

General Information on Analysis Methods and Synthesis. Commercially available reagents were purchased from Merck (Sigma-Aldrich), Alfa-Aesar, TCI Europe, and used as received. All reactions were monitored by thin-layer chromatography (TLC) performed on glass-backed silica gel 60 F254, 0.2 mm plates (Merck), and compounds were visualized under UV light (254 nm). ^{1}H and ^{13}C NMR spectra were recorded on a Bruker Avance III HD spectrometer (600 MHz for ^{1}H and 150 MHz for ^{13}C) at 298 K . Proton chemical shifts are expressed in parts per million (ppm, δ scale) and are referred to as the residual hydrogen in the solvent ($CDCl_3$, 7.27 ppm or DMSO-d^6, 2.54 ppm). Data are represented as follows: chemical shift, multiplicity (s = singlet, d = doublet, t = triplet, q = quartet, m = multiplet and/or multiple resonances, coupling constant (J) in Hertz (Hz). Carbon chemical shifts are expressed in parts per million (ppm, δ scale) and are referenced to the carbon resonances of the NMR solvent ($CDCl_3$, δ 77.0 ppm or δ DMSO-d^6, δ 39.5 ppm). Deuterated NMR solvents were purchased from Aldrich. High-resolution mass spectra were recorded on a Thermo-Fisher ESI-MS/MSORBITRAP-ELITE and Velos PRO. The sample solutions were infused directly into the ESI source using a programmable syringe pump at a flow rate of 5 μL/min. Melting points were determined in an open capillary using a Büchi melting point apparatus and were uncorrected. All the experiments were carried out in duplicate to ensure reproducibility of the experimental data. Yields refer to pure isolated materials.

X-Ray crystallography

Single crystal X-ray diffraction data for **BTCz7-Cum** and **(*t*-Bu)Cz7-Cum** were collected at 298 and 100 K, respectively, on a Bruker D8 Venture diffractometer equipped with a PHOTON II detector. The structures were solved with the ShelX[1] solution program using dual methods, and the models were refined with ShelXL 2019/1[2] using full-matrix least-squares minimization on F2. Olex2 1.5[3] was used as the graphical interface. All hydrogen atoms were added in calculated positions and refined in riding positions relative to those of the parent atom.

Quantum chemical calculations. Density functional theory (DFT) and TD(A)-DFT calculations were performed using the Gaussian 16 revision A03 package.[4] The ground-state optimization was carried out using DFT using the PBE0 functional[5], the 6-31G(d,p) basis set[6], and in conjunction with the D3(BJ)[7] dispersion correction scheme in the gas phase, starting from a structure drawn and optimized using Chem3D. The vertical excited-state calculations were performed using Time-Dependent DFT within the Tamm-Dancoff approximation (TDA-DFT),[8] with the same functional and basis set as for the ground-state geometry optimization in the gas phase. Spin-orbit coupling matrix elements SOCME were calculated based on the optimized ground state geometry using PySOC.[9] The molecular orbital distributions were visualized with GaussView 6.0.[10] The RMSD of the S0 and S1 states were visualized using the VMD program.[11] Wavefunction calculations were performed using the Turbomole 7.5[12-13] package using the resolution of the identity approximation.[14] The spin-component scaling (SCS) modification was applied to all calculations.[15] Calculations were submitted and processed using the Digichem software package,[16-17] which incorporates open-source libraries such as cclib[18] for parsing of result files, VMD[11]/Tachyon[19] for 3D rendering, Matplotlib for drawing of graphs,[20] Open Babel[21]/Pybe[22] file interconversion.

Electrochemical Measurements. Cyclic Voltammetry (CV) and Differential Pulse Voltammetry (DPV) measurements were performed on an Electrochemical Analyzer potentiostat model 620E from CH Instruments. Samples were prepared as dichloromethane (DCM) solution and degassed by flushing with DCM-saturated argon gas for 5 minutes before measurements. All measurements were carried out in 0.1 M DCM solutions of tetra-*n*-butylammonium hexafluorophosphate, $[^{n}Bu_4N]PF_6$. Ag/Ag^+ electrode was used as a reference electrode, while a glassy carbon and platinum wire were used as the working and auxiliary electrode, respectively. The redox potentials are reported relative to a saturated calomel electrode (SCE) with a ferrocenium/ferrocene (Fc/Fc^+) redox couple as the internal standard (0.46 V vs SCE for DCM).[23]

Photophysical characterization of ***Cz7-Cum, (t-Bu)Cz7-Cum, BTCz7-Cum*** *and* ***ICz7-Cum***. Optically dilute solutions of concentrations on the order of 10^{-5} or 10^{-6} M were prepared in HPLC-grade solvents for absorption and photoluminescence (PL) spectral analysis. Absorption spectra were recorded at room temperature on a

Shimadzu UV-2600 double beam spectrophotometer with a 1 cm quartz cuvette. Molar absorptivity determination was verified by linear regression analysis of values obtained from at least five independent solutions at varying concentrations ranging from 3.0×10^{-6} to 1.0×10^{-5} M. For emission studies, aerated solutions, steady-state PL and excitation spectra and time-resolved PL measurements were recorded at room temperature using an Edinburgh Instruments FS5 fluorimeter. Samples were excited at 320, 360 and 400 nm for steady-state PL measurements and 375 nm for time-resolved PL decays. Excitation spectra were recorded at emission wavelengths (λ_{PL}) of 477, 507, 522 and 557 nm. Aerated solution-state samples were prepared by bubbling solutions with compressed air for 5 minutes and spectra were measured using the cuvette for absorption analysis. Degassed solutions were prepared via three freeze-pump-thaw cycles and spectra were measured using a home-made Schlenk quartz cuvette. For solid-state film measurements, the 10 wt% doped films of emitters in a host matrix, 90% w/w of host was dissolved in 1 mL of chlorobenzene and to this, 10% w/w of emitter was added. Thin films were then spin-coated onto a sapphire substrate using a spin speed of 2000 rpm for 60 s. An integrating sphere (Edinburgh Instruments FS5, SC30 module) was employed for the photoluminescence quantum yield measurements of thin film samples. The Φ_{PL} of the films were then measured in air and in N_2 by purging the integrating sphere with N_2 gas flow for 2 min. The photophysical properties of the film samples were measured using an Edinburgh Instruments FS5 fluorimeter. Time-resolved PL measurements of the thin films were carried out using the multi-channel scaling (MCS) and time-correlated single-photon counting (TCSPC) technique. The samples were excited at 375 nm by a pulsed laser and were kept in a vacuum of $< 8 \times 10^{-4}$ mbar. The singlet and triplet state energies in 2-MeTHF glass and in doped film were determined from the onset values of the steady-state PL (SS PL) and delayed emission spectra at 77 K. The singlet-triplet energy gap (ΔE_{ST}) was estimated from the difference in energy of the onsets of these spectra. For SS PL, samples were excited by a xenon lamp emitting at 320, 360 and 400 nm. Excitation spectra in solid state were recorded at λ_{PL} of 495, 518, 536 and 575 nm. For phosphorescence spectra, samples were excited by a xenon flashlamp emitting at 320, 360 and 400 nm (EI FS5, SC-70). Phosphorescence spectra were measured with a time-gated window of 1-10 ms with xenon flashlamp operating at 100 Hz.

Fitting of time-resolved photoluminescence measurements: Time-resolved PL measurements were fitted to a sum of exponentials decay model, with chi-squared (χ^2) values between 1 and 2, using the Fluoracle software. Each component of the decay is assigned a weight, (w_i), which is the contribution of the emission from each component to the total emission.

The average lifetime was then calculated using the following:

- Two exponential decay model:

$$\tau_{AVG} = \tau_1 w_1 + \tau_2 w_2$$

with weights defined as $w_1 = \frac{A1\tau_1}{A1\tau_1 + A2\tau_2}$ and $w_2 = \frac{A2\tau_2}{A1\tau_1 + A2\tau_2}$ where A1 and A2 are the preexponential-factors of each component.

- Three exponential decay model:

$$\tau_{AVG} = \tau_1 w_1 + \tau_2 w_2 + \tau_3 w_3$$

with weights defined as $w_1 = \frac{A1\tau_1}{A1\tau_1 + A2\tau_2 + A3\tau_3}$, $w_2 = \frac{A2\tau_2}{A1\tau_1 + A2\tau_2 + A3\tau_3}$ and $w_3 = \frac{A3\tau_3}{A1\tau_1 + A2\tau_2 + A3\tau_3}$ where A1, A2 and A3 are the preexponential-factors of each component

Sensing Analysis. Absorption spectra were recorded at room temperature on a Jasco V-750 UV-Vis absorption spectrophotometer. Steady-state emission spectra were recorded at room temperature using a Jasco FP-8550 spectrofluorometer. Samples were excited at 340, 360, 390, and 400 nm for steady-state PL measurements. PL Quantum yield (PLQY) measurements in the solid state were performed using an integrating sphere paired with the Jasco FP-8550 spectrofluorometer at 340, 360, 390, and 400 nm excitation wavelength (JASCO Corporation, Ishikawamachi, Hachioji, Tokyo, Japan).

Rheological measurements. Photorheological tests were performed using a Physica MCR 302, Anton Paar rheometer in the parallel plate mode. The instrument was equipped with a quartz bottom plate and a UV lamp (Hamamatsu LC8 lamp, Hamamatsu Photonics). A light intensity of 15mW/cm^2 was set and light was switched on after 30 s, to allow the system to stabilize before the onset of polymerization. The gap between the plates was fixed at 0.2 mm; time sweep measurements were performed during irradiation at a constant frequency of 1 Hz. According to preliminary amplitude sweep tests, the experiments were performed within the linear viscoelastic region (strain amplitude γ of 1%).

IR measurements. FTIR-ATR: Infrared spectra were collected using a Jasco FTIR-4XLE Spectrometer equipped with ATR tool, 64 scans were collected with a resolution of 4 cm^{-1} from 4000 to 400 cm^{-1}.

3D printing. Formulation were 3D printed using an Anycubic Mono SE 3D printer (LCD light source @405nm, light intensity 3mW/cm^2). The CAD model was converted into an STL file format and 3D printed; the layer thickness was fixed to 30 μm while the exposure time was varied according to the monomer and dye used.

Computational methodologies

Table S1. Calculated singlet excited states associated with the absorption band at ≈ 340 nm.

Compound	State (a)	E / eV (a)	λ / nm (a)	*f* (a)	Transition (Probability) (a)
**BTCz7-Cum**	S_3	3.58	347	0.11	HOMO-2 → LUMO (0.96)
**ICz7-Cum**	S_5	3.57	347	0.11	HOMO → LUMO+2 (0.91)

(a) Calculated at the PBE0/6-31G(d,p) level of theory in the gas phase.

S3 NTO

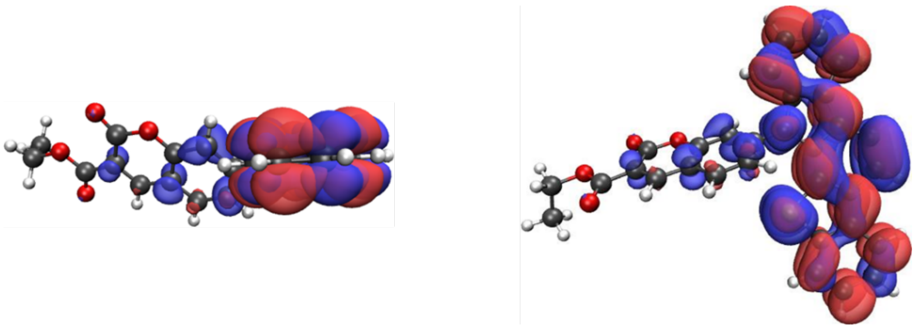

Figure S1. Natural transition orbitals [unoccupied (hole, blue) & occupied (electron, red)], of S_3 of **BTCz7-Cum** calculated at the optimized S_0 geometry at the PBE0/6-31G(d,p) level of theory in the gas phase (isovalue = 0.02).

S5 NTO

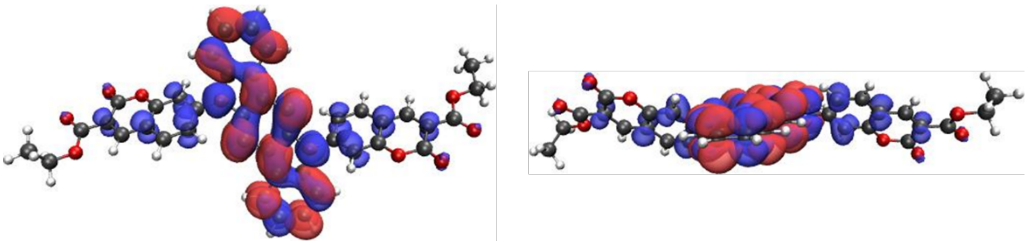

Figure S2. Natural transition orbitals [unoccupied (hole, blue) & occupied (electron, red)], of S_3 of **ICz7-Cum** calculated at the optimized S_0 geometry at the PBE0/6-31G(d,p) level of theory in the gas phase (isovalue = 0.02).

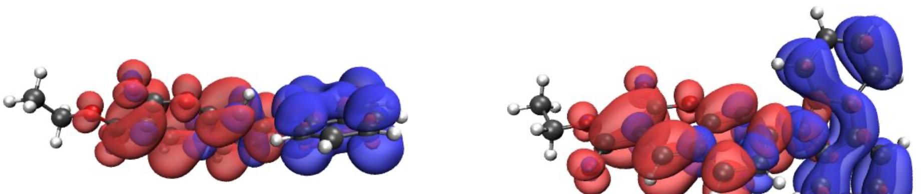

S1 NTO

T1 NTO

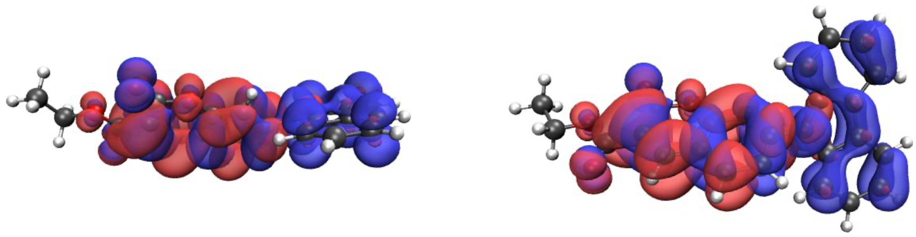

Figure S3. Natural transition orbitals [unoccupied (hole, blue) & occupied (electron, red)], of S_1 and T_1 of **Cz7-Cum** calculated at the optimized S_0 geometry at the PBE0/6-31G(d,p) level of theory in the gas phase (isovalue = 0.02).

S1 NTO

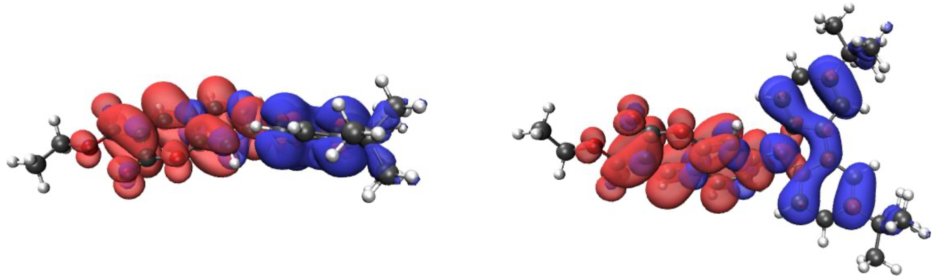

T1 NTO

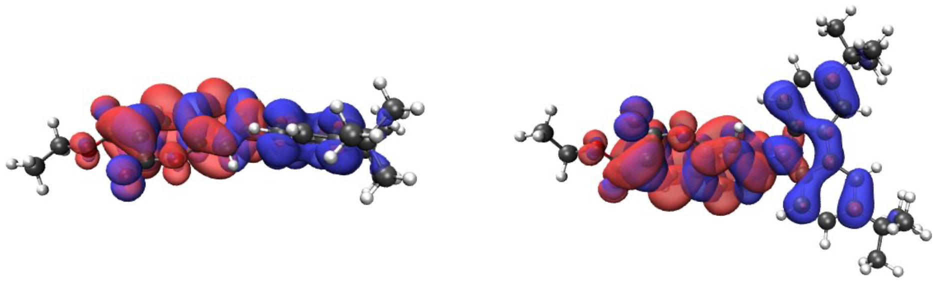

Figure S4. Natural transition orbitals [unoccupied (hole, blue) & occupied (electron, red)], of S_1 and T_1 of ***(t*-Bu)Cz7-Cum** calculated at the optimized S_0 geometry at the PBE0/6-31G(d,p) level of theory in the gas phase (isovalue = 0.02).

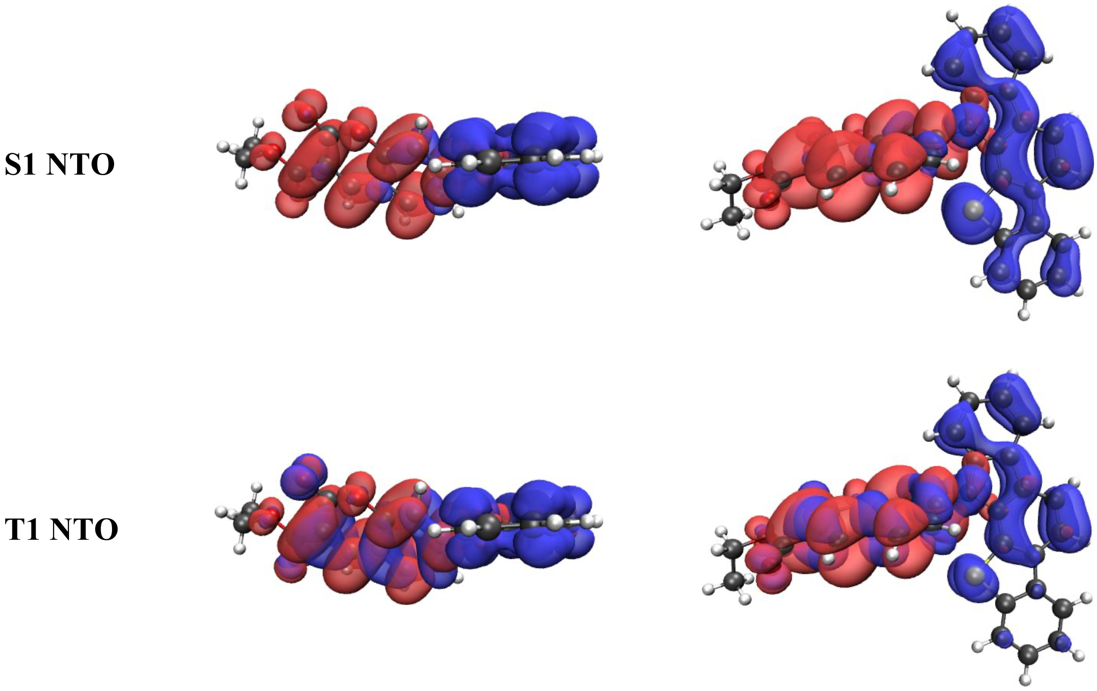

Figure S5. Natural transition orbitals [unoccupied (hole, blue) & occupied (electron, red)], of S_1 and T_1 of **BTCz7-Cum** calculated at the optimized S_0 geometry at the PBE0/6-31G(d,p) level of theory in the gas phase (isovalue = 0.02).

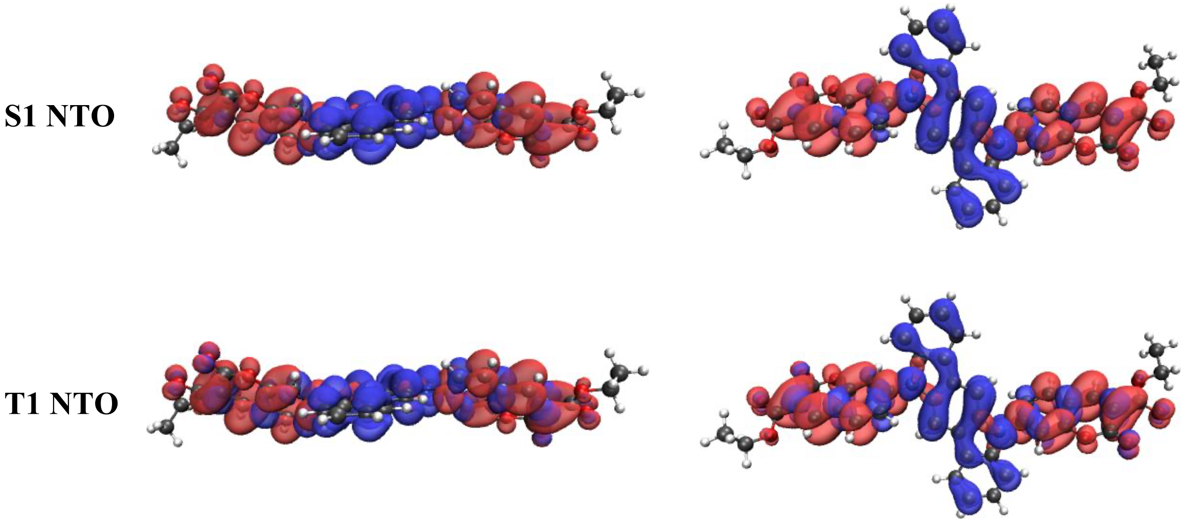

Figure S6. Natural transition orbitals [unoccupied (hole, blue) & occupied (electron, red)], of S_1 and T_1 of **ICz7-Cum** calculated at the optimized S_0 geometry at the PBE0/6-31G(d,p) level of theory in the gas phase (isovalue = 0.02).

Synthesis

Ethyl 7-bromo-2-oxo-2H-chromene-3-carboxylate (Br7-Cum)

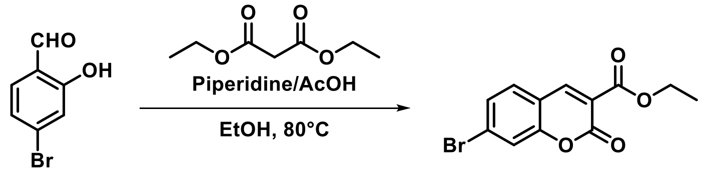

4-bromo-2-hydroxybenzaldehyde (1.00 g, 4.98 mmol, 1.00 equiv.), diethyl malonate (0.76 mL, 4.98 mmol, 1.00 equiv.), piperidine (0.05 mL, 0.50 mmol, 10 mol%), and acetic acid (5 drops) were dissolved in ethanol

(15 mL) and stirred at 80 °C for 16 hours. The reaction mixture was concentrated under vacuum and the obtained crude solid was crystallized from methanol, yielding **Br7-Cum** in 82% (1.20 g) yield as pale-yellow crystals. Spectroscopic data are in agreement with the literature.[24]

General procedure for synthesis of Cz7-Cum, (*t*-Bu)Cz7-Cum, BTCz7-Cum

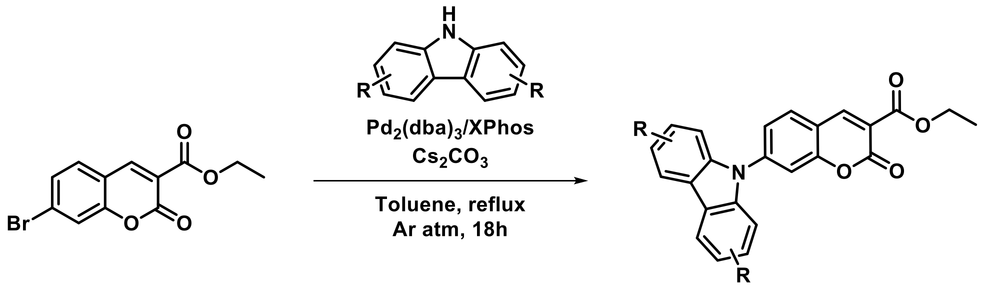

To a round-bottom flask were added **Br7-Cum** (1.00 equiv.), the corresponding carbazole derivative (1.00 equiv.), Cs_2CO_3 (3.00 equiv.), and XPhos (0.10 equiv.) under an inert atmosphere. The flask was subjected to three vacuum–argon purging cycles. $Pd_2(dba)_3$ (0.05 equiv.) was then added under a constant flow of argon. Dry toluene (10 mL) was added, and the reaction mixture was heated to reflux and stirred for 18 h. The reaction mixture was cooled to room temperature, diluted with 15 mL dichloromethane (DCM), and filtered through a short plug of celite. The filtrate was washed multiple times with DCM. The combined organic layers were washed with water (3 × 10 mL). The organic phase was separated, dried over anhydrous Na_2SO_4, filtered, and concentrated under reduced pressure. The residue was purified by flash column chromatography for **BTCz7-Cum** or recrystallisation for **Cz7-Cum** and **(*t*-Bu)Cz7-Cum** to afford the corresponding products. Specific purification details for each compound are provided in the corresponding experimental descriptions.

Ethyl 7-(9H-carbazol-9-yl)-2-oxo-2H-chromene-3-carboxylate (Cz7-Cum)

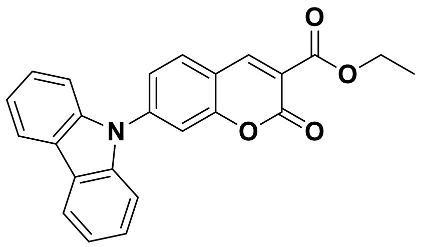

Purification was carried out by hot recrystallisation from EtOH. Pure product was obtained as yellow crystals. **Yield:** 64% (83 mg). **Mp:** 163-165 °C. **^{1}H NMR (600 MHz, CDCl$_3$) δ (ppm):** 8.62 (s, 1H), 8.14 (d, *J* = 7.3 Hz, 2H), 7.82 (d, *J* = 8.1 Hz, 1H), 7.66 – 7.61 (m, 2H), 7.56 (d, *J* = 8.3 Hz, 2H), 7.45 (ddd, *J* = 8.3, 7.1, 1.3 Hz, 2H), 7.35 (ddd, *J* = 7.9, 7.1, 0.9 Hz, 2H), 4.46 (q, *J* = 7.2 Hz, 2H), 1.45 (t, *J* = 7.2 Hz, 3H). **^{13}C NMR (151 MHz, CDCl$_3$) δ (ppm):** 163.4, 156.7, 156.7, 148.2, 143.9, 140.0, 131.2, 126.8, 124.6, 122.98, 121.7, 121.0, 118.1, 116.6, 114.2, 110.1, 62.5, 14.6. **HRMS[M+H]$^+$** Calculated: 383.1157 ($C_{24}H_{17}NO_4$); Found: 384.1219.

Ethyl 7-(3,6-di-tert-butyl-9H-carbazol-9-yl)-2-oxo-2H-chromene-3-carboxylate ((*t*-Bu)Cz7-Cum)

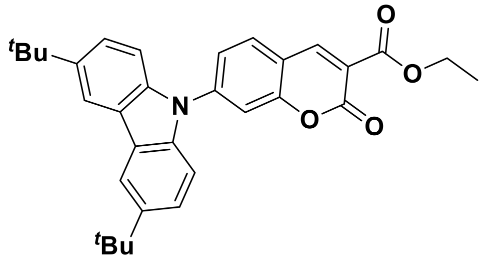

Purification was carried out by hot recrystallisation from EtOH. Pure product was obtained as yellow crystals. **Yield:** 57% (96 mg). **Mp:** 212-215 °C. **^{1}H NMR (600 MHz, CDCl$_3$) δ (ppm):** 8.61 (s, 1H), 8.15 – 8.11 (m, 2H), 7.81 – 7.77 (m, 1H), 7.65 – 7.60 (m, 2H), 7.53 – 7.47 (m, 4H), 4.46 (q, *J* = 7.1 Hz, 2H), 1.47 (s, 18H), 1.44 (d, *J* = 7.1 Hz, 3H). **^{13}C NMR (151 MHz, CDCl$_3$) δ (ppm):** 163.1, 156.4, 156.4, 148.0, 144.4, 144.1, 138.0, 130.7, 124.3, 124.1, 122.0, 117.2, 116.5, 115.7, 113.1, 109.3, 62.0, 34.7, 31.8, 14.2. **HRMS[M+H]$^+$** Calculated: 495.2410 ($C_{32}H_{33}NO_4$); Found: 496.2468.

Ethyl 7-(12H-benzo[4,5]thieno[2,3-a]carbazol-12-yl)-2-oxo-2H-chromene-3-carboxylate (BTCz7-Cum)

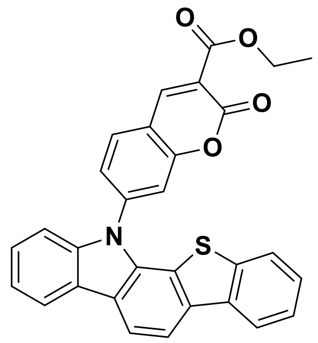

Purification was carried out by flash column chromatography PE/EtOAc 6:1→2:1, which allowed for the elution of the desired compound. Pure product was obtained as yellow solid. **Yield:** 52% (93 mg). **Mp:** 274-275 °C. **^{1}H NMR (600 MHz, DMSO) δ (ppm):** 8.99 (s, 1H), 8.48 – 8.41 (m, 2H), 8.41 – 8.34 (m, 2H), 8.28 (d, J = 8.2 Hz, 1H), 7.96 (d, J = 8.0 Hz, 1H), 7.91 (d, J = 2.0 Hz, 1H), 7.76 (dd, J = 8.2, 2.1 Hz, 1H), 7.56 – 7.51 (m, 1H), 7.53 – 7.45 (m, 2H), 7.43 – 7.36 (m, 2H), 4.37 (q, J = 7.1 Hz, 2H), 1.37 (t, J = 7.1 Hz, 3H). **^{13}C NMR (151 MHz, DMSO) δ (ppm):** 162.3, 155.5, 154.7, 147.8, 140.5, 140.5, 138.0, 134.8, 134.7, 134.2, 131.2, 126.4, 126.2, 125.1, 124.7, 123.1, 122.6, 121.6, 121.5, 120.9, 120.4, 120.0, 118.0, 117.9, 117.6, 116.4, 114.6, 109.8, 61.1, 13.8. **HRMS[M+H]$^+$** Calculated: 489.1035 ($C_{30}H_{19}NO_4S$); Found: 490.1097.

Diethyl 7,7'-(indolo[3,2-b]carbazole-5,11-diyl)bis(2-oxo-2H-chromene-3-carboxylate) (ICz7-Cum)

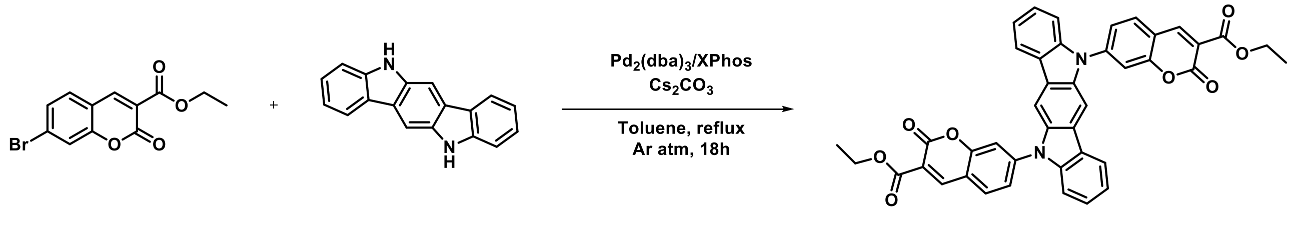

To a round-bottom flask were added **Br7-Cum** (383 mg, 1.30 mmol, 2.20 equiv.), 5,11-dihydroindolo[3,2-b]carbazole (150 mg, 0.58 mmol, 1.00 equiv.), Cs_2CO_3 (572 mg, 1.76 mmol, 3.00 equiv.), and XPhos (28 mg, 0.06 mmol, 0.10 equiv.) under an inert atmosphere. The flask was subjected to three vacuum–argon purging cycles. $Pd_2(dba)_3$ (43 mg, 0.05 mmol, 0.08 equiv.) was then added under a constant flow of argon. Dry toluene (20 mL) was added, and the reaction mixture was heated to reflux and stirred for 18 h. The reaction mixture was cooled to room temperature, diluted with 25 mL DCM, and filtered through a short plug of celite. The filtrate was washed multiple times with DCM. The combined organic layers were subjected to extraction and washed with water (3 × 20 mL). The organic phase was separated, dried over anhydrous Na_2SO_4, filtered, and

concentrated under reduced pressure. After vacuum concentration, the residue was purified by hot-trituration with MeOH to afford the corresponding products as orange powder. **Yield:** 26% (105 mg). **Mp**: 318-320 °C. **^{1}H NMR (600 MHz, DMSO) δ (ppm):** 8.96 (s, 2H), 8.46 (s, 2H), 8.38 (d, *J* = 7.8 Hz, 2H), 8.27 (d, *J* = 8.3 Hz, 2H), 7.91 (d, *J* = 2.0 Hz, 2H), 7.86 (dd, *J* = 8.2, 2.1 Hz, 2H), 7.61 (d, *J* = 8.2 Hz, 2H), 7.49 (ddd, *J* = 8.3, 7.0, 1.2 Hz, 2H), 7.32 (t, *J* = 7.6 Hz, 2H), 4.36 (q, *J* = 7.1 Hz, 4H), 1.37 (t, *J* = 7.1 Hz, 6H). **^{13}C NMR (151 MHz, DMSO) δ (ppm):**163.0, 156.2, 156.2, 148.6, 142.9, 140.8, 136.1, 132.4, 127.1, 123.9, 123.0, 121.4, 121.0, 117.3, 116.8, 113.8, 110.1, 101.2, 61.6, 14.4. **HRMS[M+H]$^+$** Calculated: 688.1846 ($C_{42}H_{28}N_2O_8$); Found: 689.1570.

^{1}H and ^{13}C NMR spectral characterization

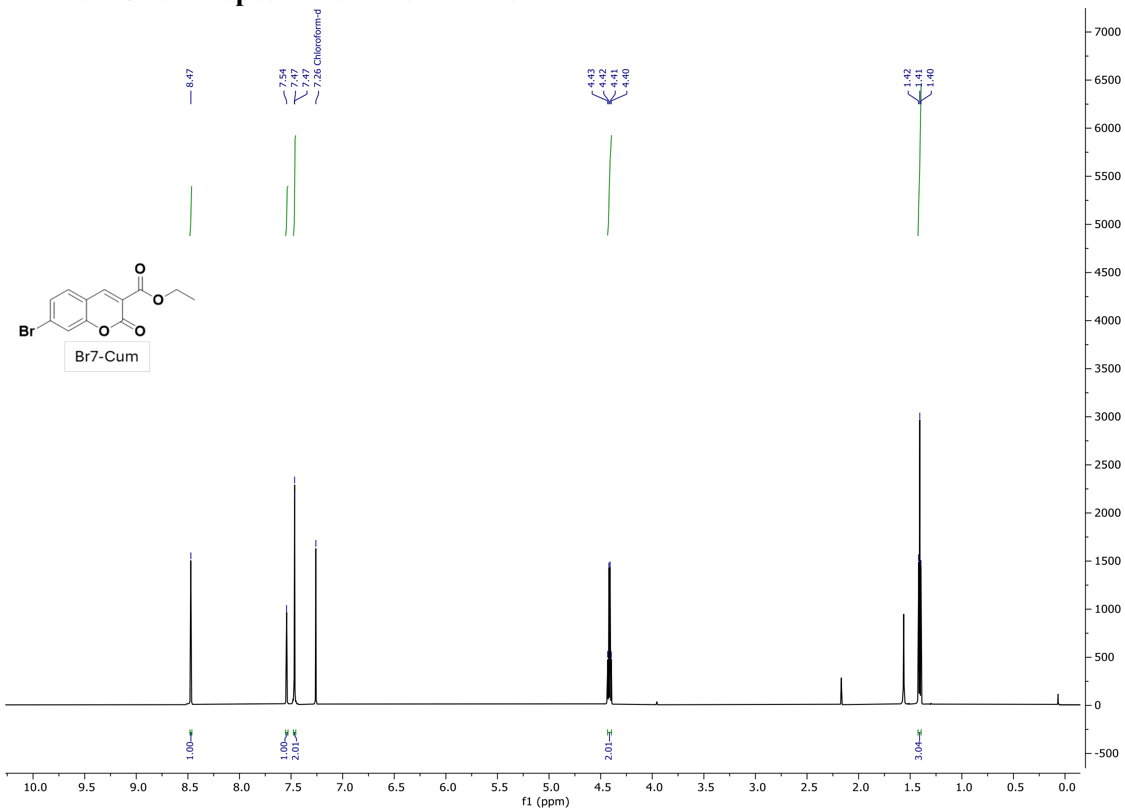

Figure S7. ^{1}H NMR spectrum of **Br7-Cum** in $CDCl_3$.

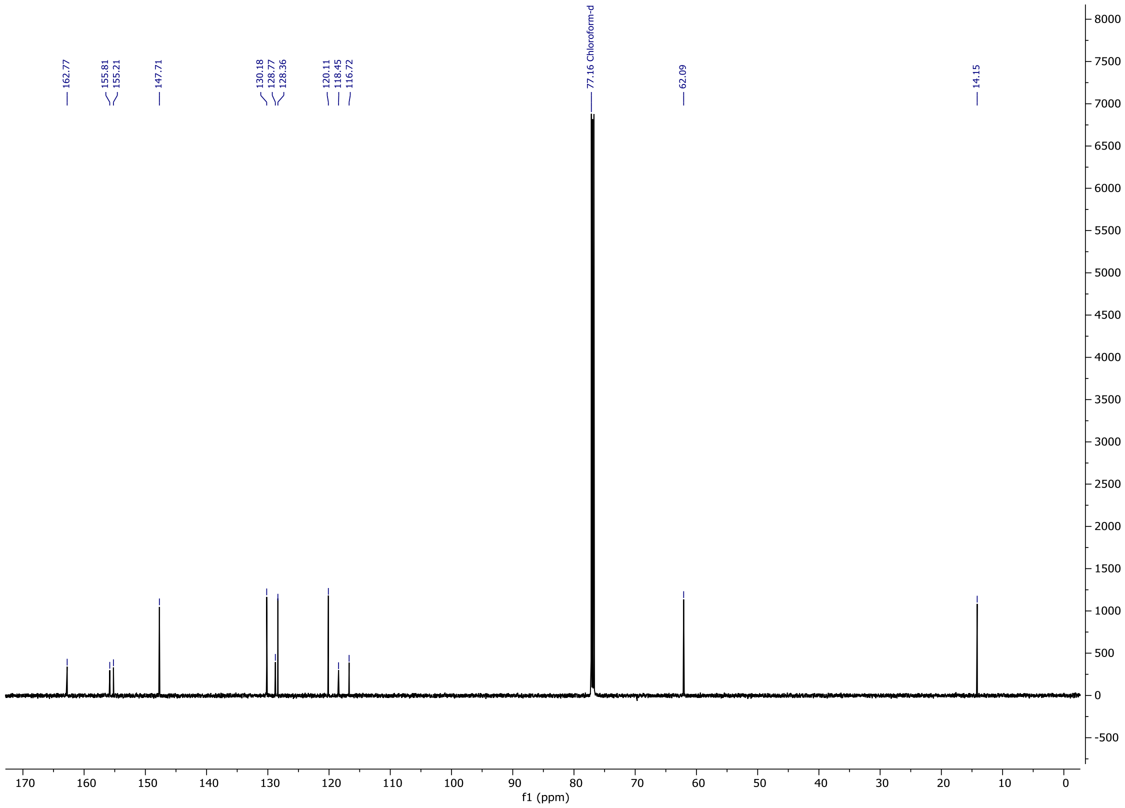

Figure S8. ^{13}C NMR spectrum of **Br7-Cum** in $CDCl_3$.

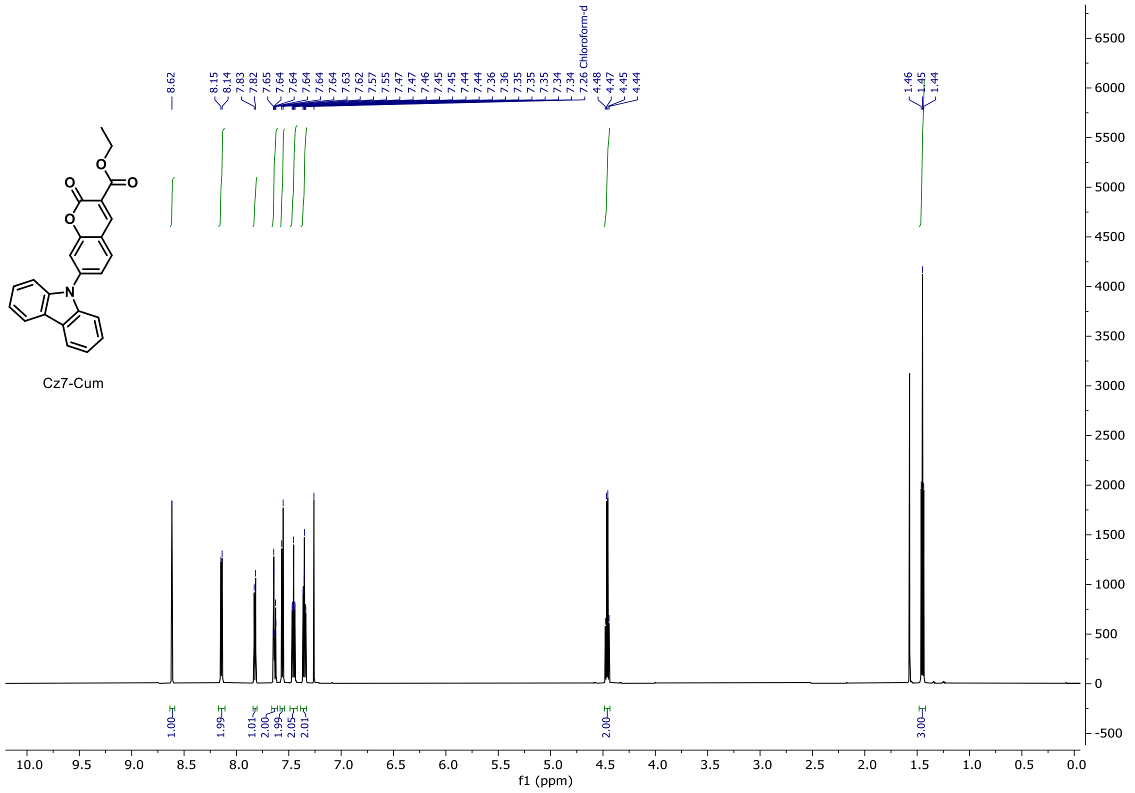

Figure S9. ^{1}H NMR spectrum of **Cz7-Cum** in $CDCl_3$.

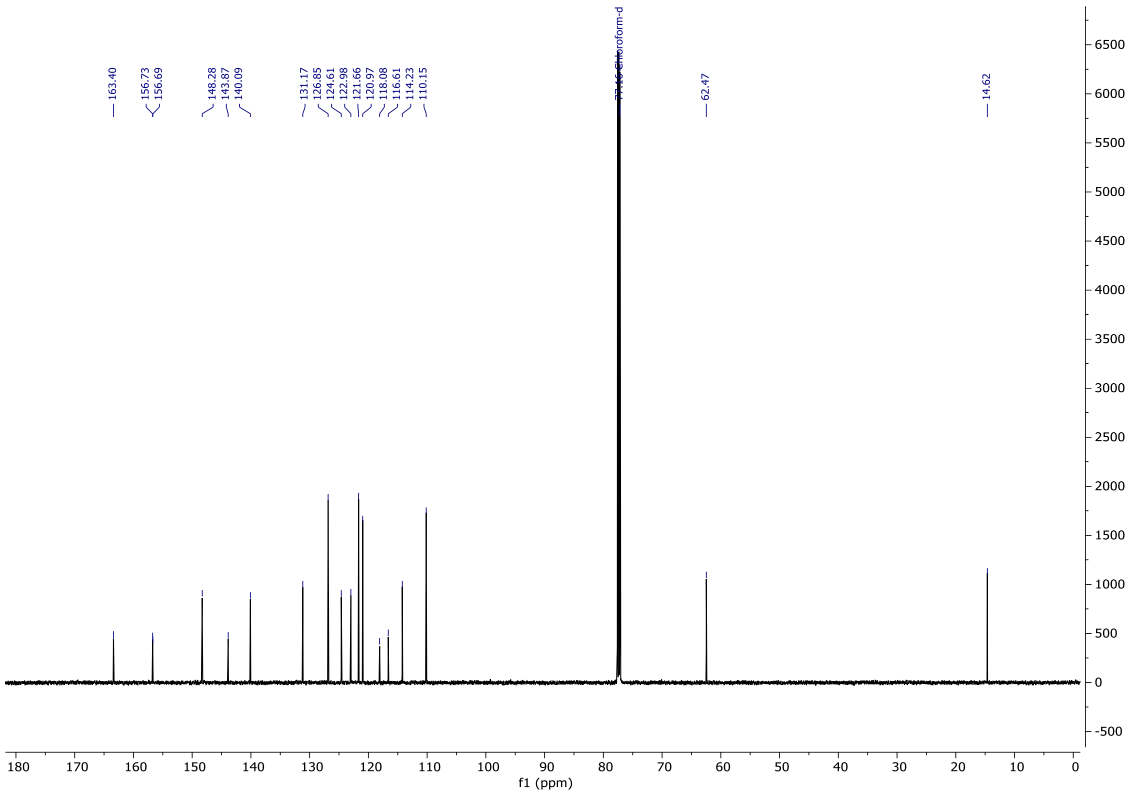

Figure S10. ^{13}C NMR spectrum of **Cz7-Cum** in $CDCl_3$.

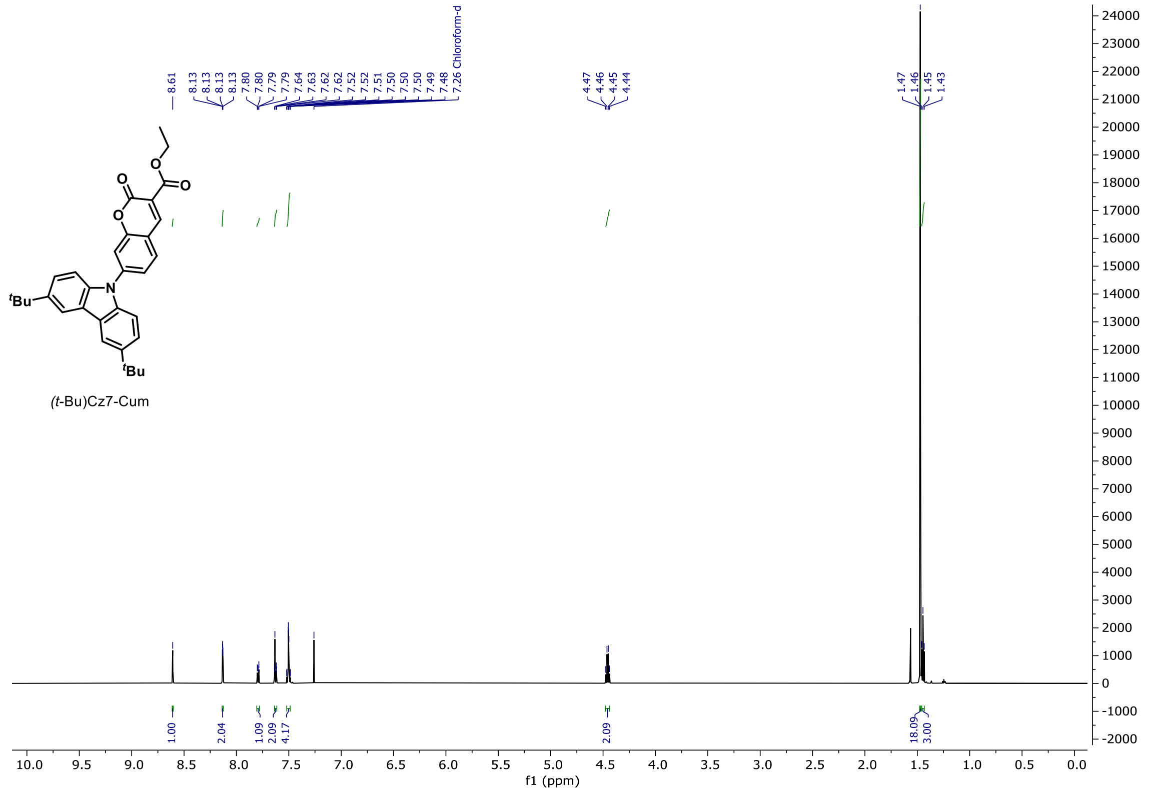

Figure S11. ^{1}H NMR spectrum of ***(t*-Bu)Cz7-Cum** in $CDCl_3$.

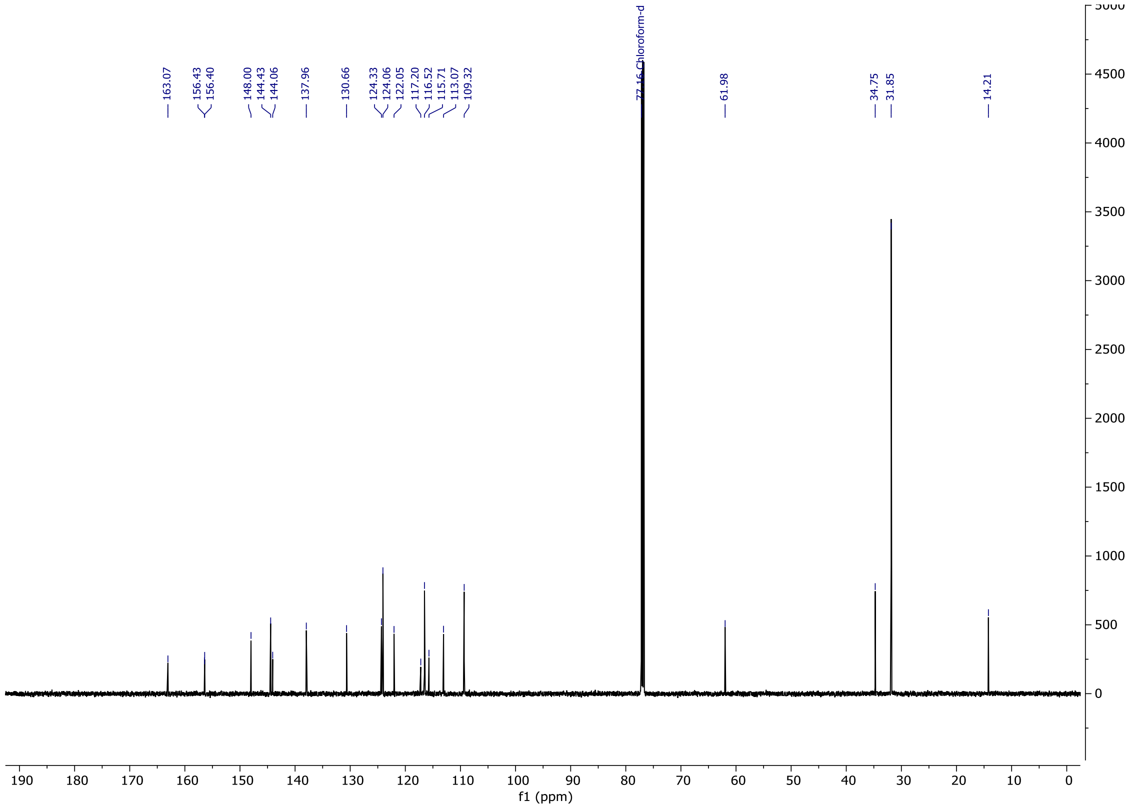

Figure S12. ^{13}C NMR spectrum of ***(t*-Bu)Cz7-Cum** in $CDCl_3$.

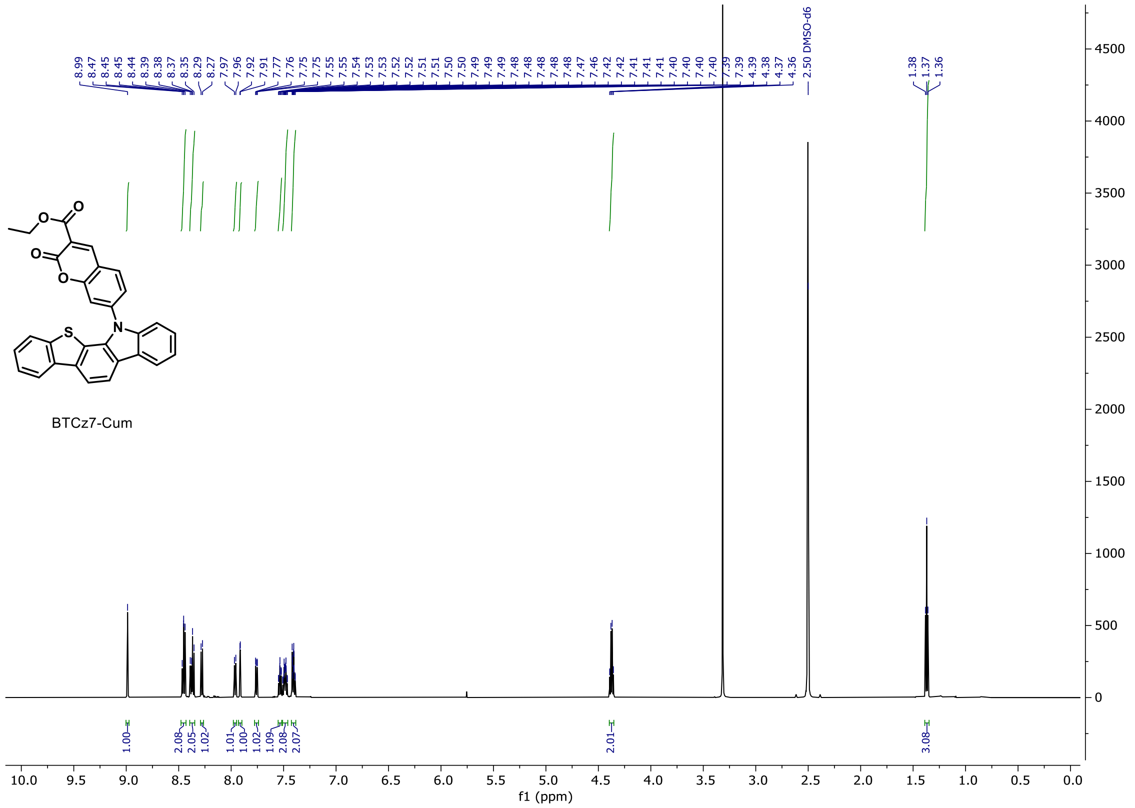

Figure S13. ^{1}H NMR spectrum of **BTCz7-Cum** in DMSO.

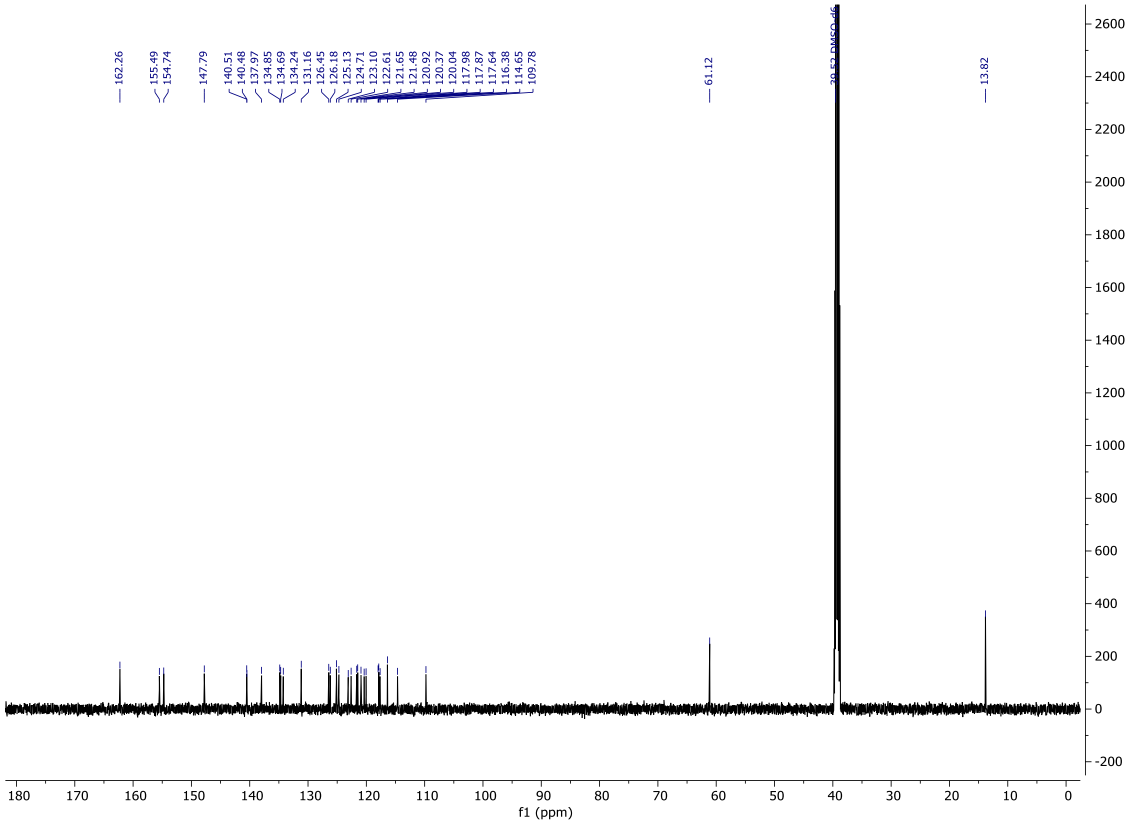

Figure S14. ^{13}C NMR spectrum of **BTCz7-Cum** in DMSO.

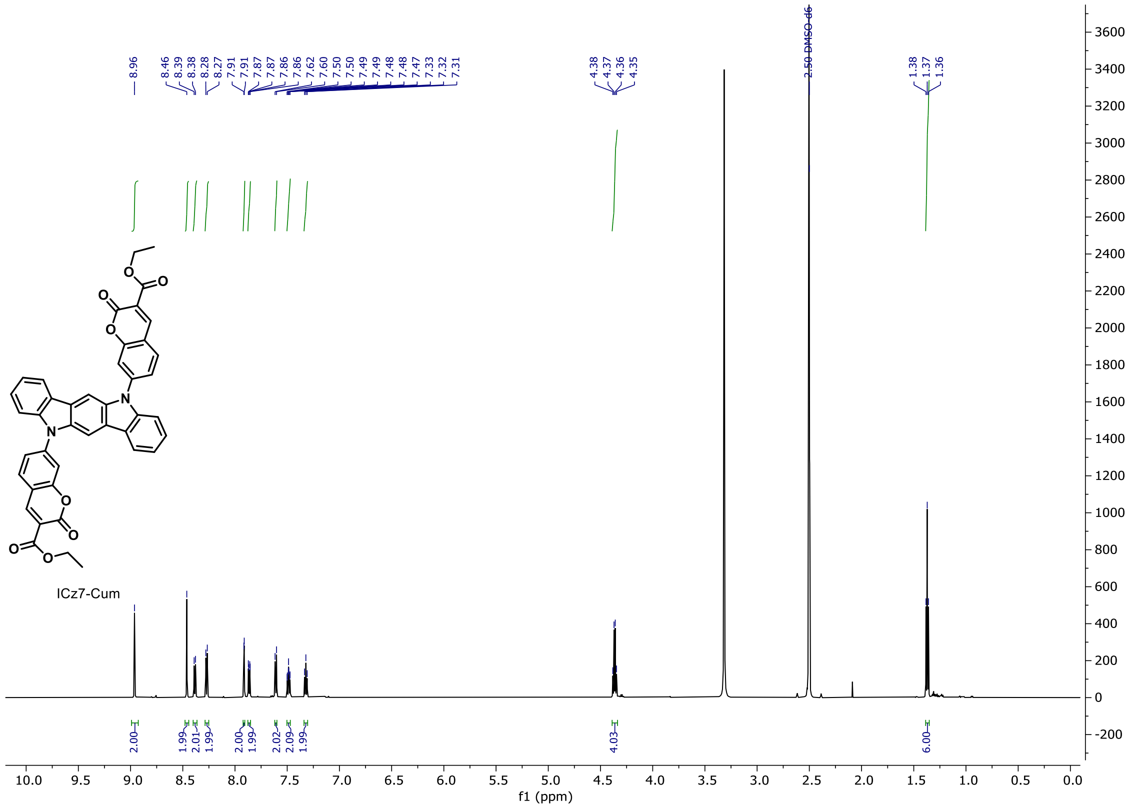

Figure S15. ^{1}H NMR spectrum of **ICz7-Cum** in DMSO.

.

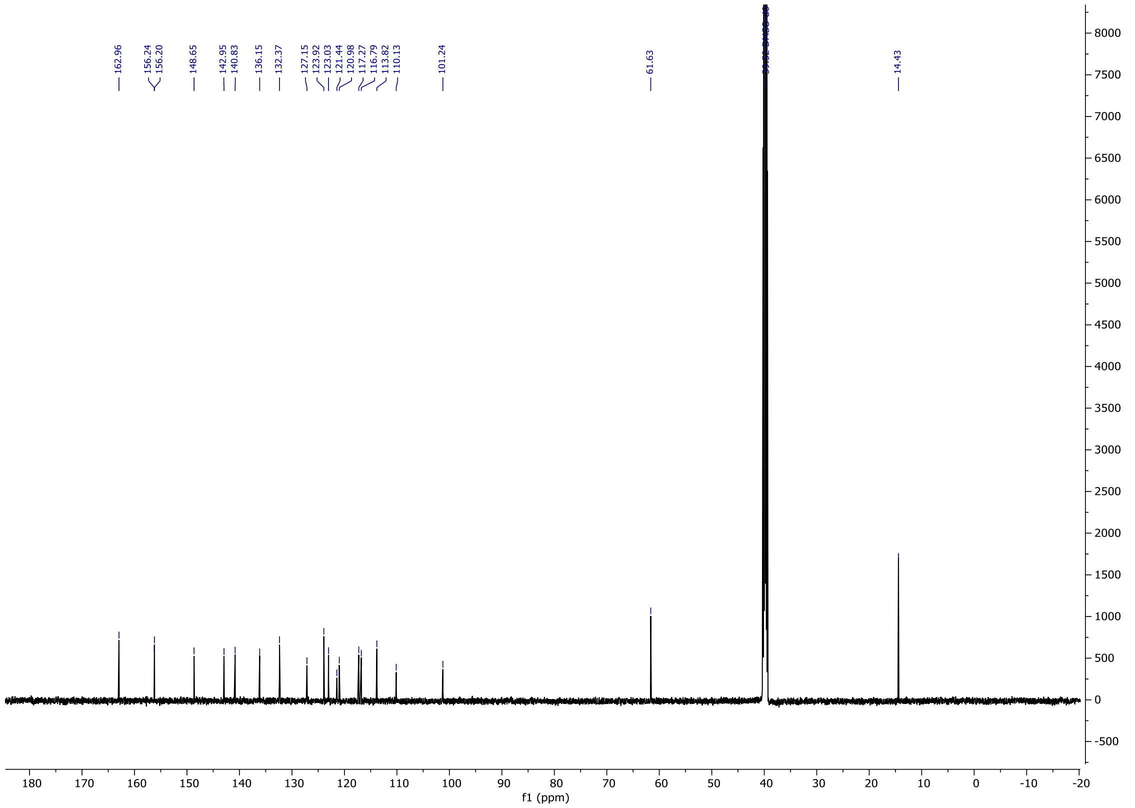

Figure S16. ^{13}C NMR spectrum of **ICz7-Cum** in DMSO.

X-Ray crystallography

Structural characterization of ***BTCz7-Cum***

Compound **BTCz7-Cum** (CCDC deposition number 2501822) was obtained as yellow prismatic crystals (chloroform/hexane). Single-crystal X-ray diffraction analysis revealed that it crystallizes in the trigonal space group $P3_121$ (**Figure S18**, **Tables S2–S4**).

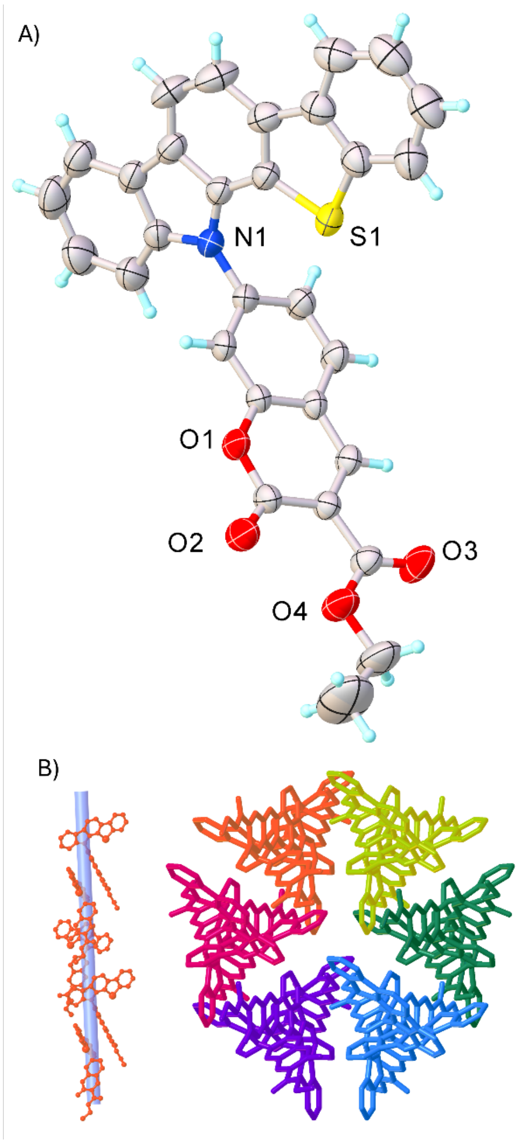

Figure S17. X-ray crystal structure of **BTCz7-Cum**: a) asymmetric unit viewed along [110] with displacement ellipsoids at 50% probability; b) partial crystal packing showing a single helix along [010] (left) and multiple helices along [001] (right).

The asymmetric unit features one independent molecule of **BTCz7-Cum**, in which the coumarin π-bridge is nearly perpendicular, twisted by about 80° with respect to the plane of the carbazole donor (Figure 1a). The crystal packing is stabilized by the cooperative interplay of noncovalent interactions (NCIs), including π–π stacking, chalcogen bonds (ChBs), and hydrogen bonds (HBs). Adjacent molecules interact through π–π stacking and weak C–S···S chalcogen bonds, generating supramolecular helices propagating along the [001] direction (**Figures S18b** and **S19**).

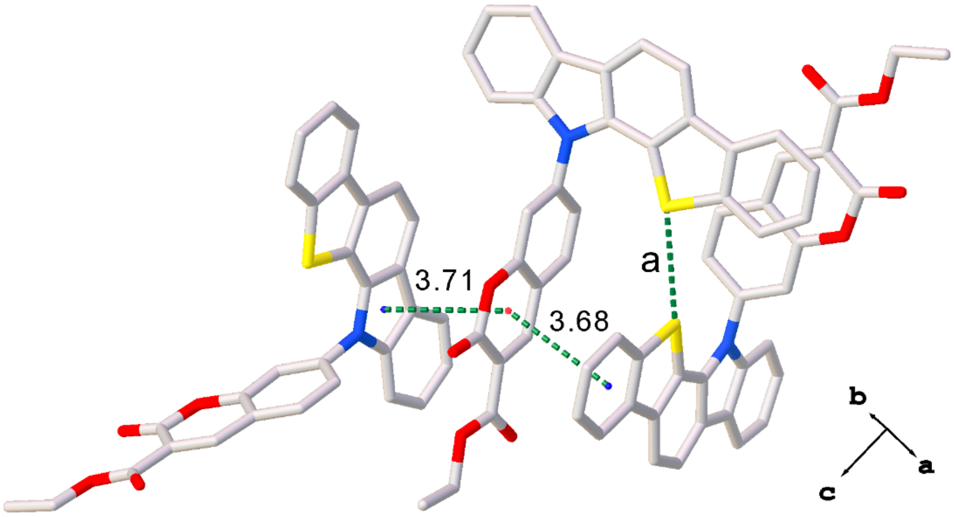

Figure S18. Intermolecular π-π stacking and chalcogen bonding interactions in **BTCz7-Cum**. Centroid-centroid distances are given in Å. a) S1···S1^i = 3.417(2) Å; ^{i} = +*y*, +*x*, 1−*z*.

Interhelical C–H···O hydrogen bonds involving aromatic C–H moieties of the coumarin and the carbonyl oxygen atoms of both the coumarin and ester groups connect neighbouring helices into a cross-linked supramolecular network (**Figure S20**).

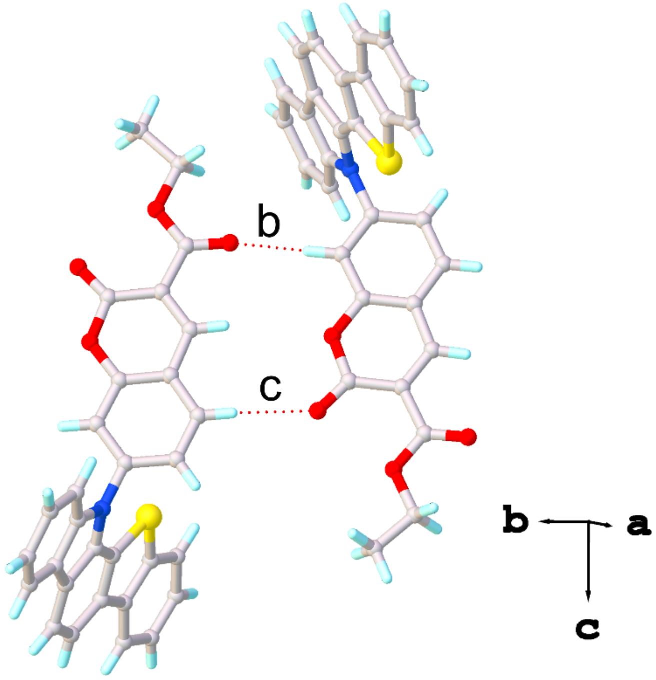

Figure S19. Intermolecular hydrogen bonding interactions found in **BTCz7-Cum**: b) C24–H24···O3^{ii} (C24···O3^{ii} 3.192(4) Å); c) C21^{ii}–H21^{ii}···O2 (C21^{ii}···O2 3.294(5) Å). ^{ii} = 1−*x*, 1−*x*+*y*, 4/3−*z*.

Table S2. Crystal data and structure refinement parameters for **BTCz7-Cum**.

Empirical formula	$C_{30}H_{19}NO_4S$
Formula weight	489.52
Temperature/K	293(2)
Crystal system	trigonal
Space group	$P3_121$
a/Å	13.2364(3)
b/Å	13.2364(3)
c/Å	23.6139(10)
α/°	90
β/°	90
γ/°	120
Volume/Å^3	3582.9(2)
Z	6
ρ_{calc}g/cm^3	1.361
μ/mm^{-1}	0.174
F(000)	1524.0
Crystal size/mm^3	0.25 × 0.18 × 0.12
Radiation	MoKα (λ = 0.71073)
2θ range for data collection/°	3.95 to 56.55
Index ranges	$-17 \leq h \leq 17, -17 \leq k \leq 17, -31 \leq l \leq 31$
Reflections collected	70964
Independent reflections	5931 [R_{int} = 0.0497, R_{sigma} = 0.0207]
Data/restraints/parameters	5931/0/326
Goodness-of-fit on F^2	1.054
Final R indexes [I ≥ 2σ (I)]	R_1 = 0.0414, wR_2 = 0.1035
Final R indexes [all data]	R_1 = 0.0505, wR_2 = 0.1091
Largest diff. peak/hole / e Å^{-3}	0.17/−0.18
Flack parameter	0.02(2)

Table S3. Bond lengths (Å) for **BTCz7-Cum**.

Atom	Atom	Length/Å	Atom	Atom	Length/Å
S1	C1	1.747(3)	C19	C20	1.397(4)
S1	C12	1.752(3)	C7	C12	1.404(4)
O1	C23	1.371(3)	C7	C6	1.447(5)
O1	C27	1.381(3)	C7	C8	1.398(4)
O3	C28	1.197(3)	C27	C26	1.458(3)
O2	C27	1.201(3)	C1	C6	1.393(4)
O4	C28	1.311(3)	C1	C2	1.386(5)
O4	C29	1.460(4)	C10	C13	1.437(4)
N1	C11	1.385(4)	C10	C9	1.393(4)
N1	C19	1.434(3)	C14	C13	1.403(4)
N1	C18	1.386(3)	C14	C15	1.360(6)
C23	C22	1.391(3)	C13	C18	1.408(4)
C23	C24	1.384(3)	C15	C16	1.395(6)
C22	C25	1.431(3)	C6	C5	1.402(5)
C22	C21	1.395(3)	C8	C9	1.365(5)
C25	C26	1.345(3)	C18	C17	1.370(5)
C24	C19	1.375(4)	C16	C17	1.377(5)
C21	C20	1.371(4)	C2	C3	1.382(5)
C28	C26	1.498(3)	C4	C5	1.373(6)
C11	C10	1.415(3)	C4	C3	1.368(6)
C11	C12	1.384(4)	C29	C30	1.460(7)

Table S4. Bond angles (°) for **BTCz7-Cum**.

Atom	Atom	Atom	Angle/˚	Atom	Atom	Atom	Angle/˚
C1	S1	C12	91.11(15)	C2	C1	C6	121.2(3)
C23	O1	C27	122.99(18)	C25	C26	C28	117.8(2)
C28	O4	C29	115.2(3)	C25	C26	C27	120.0(2)
C11	N1	C19	125.7(2)	C27	C26	C28	122.2(2)
C11	N1	C18	108.6(2)	C11	C10	C13	106.0(2)
C18	N1	C19	125.3(2)	C9	C10	C11	120.1(3)
O1	C23	C22	120.8(2)	C9	C10	C13	133.9(3)
O1	C23	C24	117.2(2)	C11	C12	S1	128.3(2)
C24	C23	C22	121.9(2)	C11	C12	C7	119.6(2)
C23	C22	C25	117.3(2)	C7	C12	S1	112.0(2)
C23	C22	C21	118.1(2)	C15	C14	C13	119.3(3)
C21	C22	C25	124.6(2)	C14	C13	C10	134.3(3)
C26	C25	C22	122.2(2)	C14	C13	C18	118.2(3)
C19	C24	C23	118.3(2)	C18	C13	C10	107.5(2)
C20	C21	C22	121.2(2)	C14	C15	C16	121.5(3)
O3	C28	O4	123.7(2)	C1	C6	C7	112.0(3)
O3	C28	C26	122.0(2)	C1	C6	C5	119.2(3)
O4	C28	C26	114.3(2)	C5	C6	C7	128.8(3)
N1	C11	C10	109.3(2)	C9	C8	C7	120.9(3)
C12	C11	N1	131.1(2)	C21	C20	C19	119.1(2)
C12	C11	C10	119.6(2)	C8	C9	C10	120.0(3)
C24	C19	N1	118.8(2)	N1	C18	C13	108.6(3)
C24	C19	C20	121.4(2)	C17	C18	N1	129.0(3)
C20	C19	N1	119.8(2)	C17	C18	C13	122.4(3)
C12	C7	C6	112.1(3)	C17	C16	C15	120.5(4)
C8	C7	C12	119.8(3)	C18	C17	C16	118.2(3)
C8	C7	C6	128.1(3)	C3	C2	C1	118.3(4)
O1	C27	C26	116.7(2)	C3	C4	C5	121.3(4)

Atom	Atom	Atom	Angle/°	Atom	Atom	Atom	Angle/°
O2	C27	O1	115.8(2)	C4	C5	C6	118.9(4)
O2	C27	C26	127.5(2)	C4	C3	C2	121.1(4)
C6	C1	S1	112.7(2)	O4	C29	C30	107.3(4)
C2	C1	S1	126.0(3)				

Structural characterization of ***(t-Bu)Cz7-Cum***

Compound **(*t*-Bu)Cz7-Cum** (CCDC deposition number 2503540) was recrystallized from chloroform/hexane, yielding yellow rod-shaped crystals. Single crystal X-ray diffraction analysis shows that the compound crystallizes in the monoclinic space group $P2_1/c$, with two crystallographically independent molecules in the asymmetric unit (**Figure S21, Tables S5-S7**).

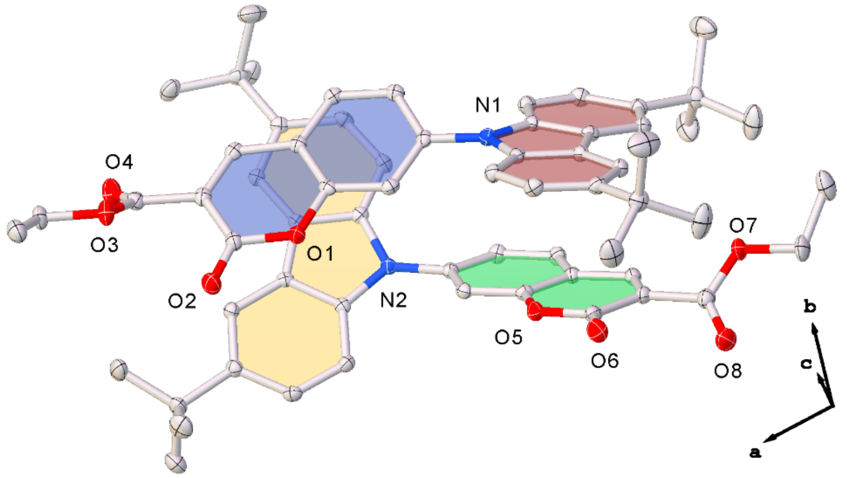

Figure S20. X-ray crystal structure of **(*t*-Bu)Cz7-Cum** with thermal ellipsoids drawn at 50% probability level. H atoms were omitted for clarity.

The two molecules differ primarily in the dihedral angle between the mean planes defined by the carbazole and coumarin fragments, which amount to 39.7° in one molecule and 45.0° in the other. The two independent molecules engage in π–π stacking interactions, exhibiting short centroid–centroid distances of 3.53 and 3.66 Å, respectively. The crystal packing is further stabilized by additional π–π interactions between symmetry-related coumarin moieties in adjacent molecules (**Figure S20**).

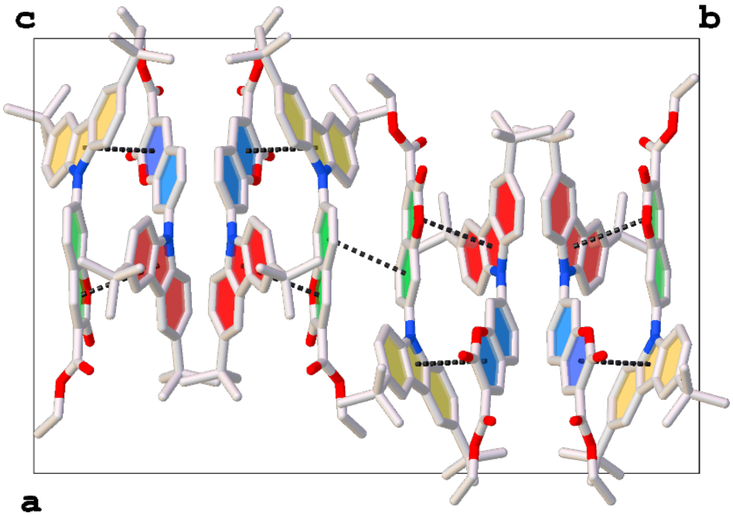

Figure S21. Portion of the crystal packing of ***(t*-Bu)Cz7-Cum** along the *c*-axis. π-π interactions are drawn as dashed black lines.

Table S5. Crystal data and structure refinement for **(*t*-Bu)Cz7-Cum**.

Empirical formula	$C_{32}H_{33}NO_4$
Formula weight	495.59
Temperature/K	100(2)
Crystal system	monoclinic
Space group	$P2_1/c$
a/Å	18.1849(15)
b/Å	27.113(2)
c/Å	10.8870(8)
α/°	90
β/°	103.320(3)
γ/°	90
Volume/Å^3	5223.5(7)
Z	8
ρ_{calc}g/cm^3	1.260
μ/mm^{-1}	0.082
F(000)	2112.0
Crystal size/mm^3	0.34 × 0.17 × 0.14
Radiation	MoKα (λ = 0.71073)
2θ range for data collection/°	4.128 to 55.07
Index ranges	$-23 \le h \le 23, -35 \le k \le 35, -12 \le l \le 14$
Reflections collected	89805
Independent reflections	11975 [R_{int} = 0.0594, R_{sigma} = 0.0307]
Data/restraints/parameters	11975/0/681
Goodness-of-fit on F^2	1.025
Final R indexes [I ≥ 2σ (I)]	R_1 = 0.0430, wR_2 = 0.0993
Final R indexes [all data]	R_1 = 0.0535, wR_2 = 0.1052
Largest diff. peak/hole / e Å^{-3}	0.54/−0.27

Table S6. Bond lengths (Å) for ***(t*-Bu)Cz7-Cum**.

Atom	Atom	Length/Å	Atom	Atom	Length/Å
O5	C41	1.3792(15)	C24	C19	1.3991(18)
O5	C33	1.3874(16)	C54	C55	1.3796(19)
O1	C9	1.3721(15)	C54	C53	1.4071(19)
O1	C1	1.3842(16)	C55	C56	1.3919(18)
O3	C10	1.3314(17)	C52	C53	1.3900(18)
O3	C11	1.4492(16)	C13	C18	1.4036(18)
O7	C42	1.3451(17)	C13	C14	1.3951(18)
O7	C43	1.4579(16)	C8	C7	1.3916(18)
O6	C33	1.1980(17)	C39	C38	1.4017(18)
O2	C1	1.1994(17)	C18	C17	1.3945(18)
O8	C42	1.2018(18)	C18	C19	1.4484(18)
O4	C10	1.2046(17)	C23	C22	1.3804(19)
N2	C45	1.4136(16)	C14	C15	1.3839(19)
N2	C56	1.4056(16)	C53	C61	1.5307(18)
N2	C39	1.4099(16)	C17	C16	1.3932(19)
N1	C24	1.4030(17)	C48	C47	1.4076(19)
N1	C13	1.4025(17)	C48	C57	1.5327(18)
N1	C7	1.4041(17)	C3	C2	1.3479(19)
C51	C50	1.4456(17)	C16	C15	1.4063(19)
C51	C56	1.3999(18)	C16	C25	1.5335(18)
C51	C52	1.3938(18)	C1	C2	1.4652(19)
C45	C50	1.4031(18)	C19	C20	1.3966(19)
C45	C46	1.3934(18)	C37	C38	1.3775(19)
C50	C49	1.3947(18)	C22	C21	1.4043(19)
C46	C47	1.3863(19)	C20	C21	1.3901(19)
C49	C48	1.3870(18)	C2	C10	1.4899(19)
C4	C5	1.4028(18)	C21	C29	1.5294(19)
C4	C9	1.3960(18)	C57	C58	1.526(2)

Atom	Atom	Length/Å	Atom	Atom	Length/Å
C4	C3	1.4286(18)	C57	C59	1.5302(19)
C40	C41	1.3780(18)	C57	C60	1.532(2)
C40	C39	1.3944(18)	C61	C63	1.532(2)
C41	C36	1.3942(18)	C61	C62	1.536(2)
C36	C35	1.4290(18)	C61	C64	1.531(2)
C36	C37	1.4044(19)	C25	C28	1.544(2)
C5	C6	1.3728(19)	C25	C26	1.519(2)
C9	C8	1.3784(19)	C25	C27	1.519(2)
C34	C35	1.3520(19)	C11	C12	1.500(2)
C34	C33	1.4650(19)	C43	C44	1.496(2)
C34	C42	1.4900(18)	C29	C31	1.536(2)
C6	C7	1.4041(18)	C29	C30	1.524(2)
C24	C23	1.3965(18)	C29	C32	1.528(2)

Table S7. Bond angles (°) for **(*t*-Bu)Cz7-Cum**.

Atom	Atom	Atom	Angle/°	Atom	Atom	Atom	Angle/°
C41	O5	C33	122.76(10)	O5	C33	C34	115.88(11)
C9	O1	C1	122.46(11)	O6	C33	O5	117.08(12)
C10	O3	C11	116.03(11)	O6	C33	C34	127.03(12)
C42	O7	C43	115.62(11)	C16	C17	C18	120.21(12)
C56	N2	C45	107.62(10)	O7	C42	C34	110.31(12)
C56	N2	C39	124.13(11)	O8	C42	O7	124.32(13)
C39	N2	C45	126.06(11)	O8	C42	C34	125.36(13)
C24	N1	C7	125.30(11)	C49	C48	C47	117.95(12)
C13	N1	C24	108.01(11)	C49	C48	C57	122.00(12)
C13	N1	C7	126.30(11)	C47	C48	C57	120.01(12)
C56	C51	C50	106.91(11)	C2	C3	C4	121.03(13)
C52	C51	C50	132.39(12)	C17	C16	C15	118.28(12)
C52	C51	C56	120.64(12)	C17	C16	C25	121.79(12)
C50	C45	N2	108.58(11)	C15	C16	C25	119.91(12)
C46	C45	N2	131.63(12)	O1	C1	C2	115.93(11)
C46	C45	C50	119.80(12)	O2	C1	O1	116.66(12)
C45	C50	C51	107.52(11)	O2	C1	C2	127.38(13)
C49	C50	C51	131.52(12)	C14	C15	C16	122.66(12)
C49	C50	C45	120.95(12)	C24	C19	C18	106.82(11)
C47	C46	C45	118.23(12)	C20	C19	C24	120.35(12)
C48	C49	C50	120.09(12)	C20	C19	C18	132.83(12)
C5	C4	C3	124.86(12)	C6	C7	N1	120.69(12)
C9	C4	C5	117.83(12)	C8	C7	N1	119.15(12)
C9	C4	C3	117.31(12)	C8	C7	C6	120.15(12)
C41	C40	C39	119.15(12)	C46	C47	C48	122.97(12)
O5	C41	C36	121.19(12)	C38	C37	C36	121.12(12)
C40	C41	O5	116.58(11)	C23	C22	C21	123.21(13)
C40	C41	C36	122.22(12)	C21	C20	C19	120.48(12)

Atom	Atom	Atom	Angle/°	Atom	Atom	Atom	Angle/°
C41	C36	C35	117.73(12)	C3	C2	C1	121.40(12)
C41	C36	C37	117.71(12)	C3	C2	C10	118.63(12)
C37	C36	C35	124.54(12)	C1	C2	C10	119.96(12)
C6	C5	C4	121.10(12)	C37	C38	C39	119.83(12)
O1	C9	C4	121.69(12)	C22	C21	C29	119.43(12)
O1	C9	C8	116.22(12)	C20	C21	C22	117.65(12)
C8	C9	C4	122.09(12)	C20	C21	C29	122.90(12)
C35	C34	C33	121.30(12)	C58	C57	C48	112.38(11)
C35	C34	C42	121.89(12)	C58	C57	C59	107.72(12)
C33	C34	C42	116.79(12)	C58	C57	C60	108.17(13)
C5	C6	C7	119.76(12)	C59	C57	C48	108.68(11)
C34	C35	C36	121.12(12)	C59	C57	C60	109.49(13)
C23	C24	N1	130.60(12)	C60	C57	C48	110.34(12)
C23	C24	C19	120.16(12)	O3	C10	C2	111.92(11)
C19	C24	N1	109.19(11)	O4	C10	O3	124.92(13)
C55	C54	C53	123.21(12)	O4	C10	C2	123.13(13)
C54	C55	C56	117.74(12)	C53	C61	C63	109.31(11)
C51	C56	N2	109.37(11)	C53	C61	C62	109.67(12)
C55	C56	N2	129.93(12)	C63	C61	C62	109.02(12)
C55	C56	C51	120.51(12)	C64	C61	C53	111.88(11)
C53	C52	C51	119.83(12)	C64	C61	C63	108.33(13)
N1	C13	C18	108.71(11)	C64	C61	C62	108.58(12)
C14	C13	N1	130.65(12)	C16	C25	C28	108.26(12)
C14	C13	C18	120.59(12)	C26	C25	C16	110.79(12)
C9	C8	C7	118.96(12)	C26	C25	C28	108.01(13)
C40	C39	N2	119.03(11)	C27	C25	C16	111.64(12)
C40	C39	C38	119.93(12)	C27	C25	C28	108.47(14)
C38	C39	N2	121.03(12)	C27	C25	C26	109.55(14)
C13	C18	C19	107.26(11)	O3	C11	C12	106.71(11)

Atom	Atom	Atom	Angle/°	Atom	Atom	Atom	Angle/°
C17	C18	C13	120.15(12)	O7	C43	C44	106.76(12)
C17	C18	C19	132.55(12)	C21	C29	C31	110.85(12)
C22	C23	C24	118.14(12)	C30	C29	C21	111.32(12)
C15	C14	C13	118.11(12)	C30	C29	C31	107.03(13)
C54	C53	C61	118.81(12)	C30	C29	C32	110.32(15)
C52	C53	C54	118.04(12)	C32	C29	C21	108.46(12)
C52	C53	C61	123.12(12)	C32	C29	C31	108.83(14)

Electrochemistry characterization

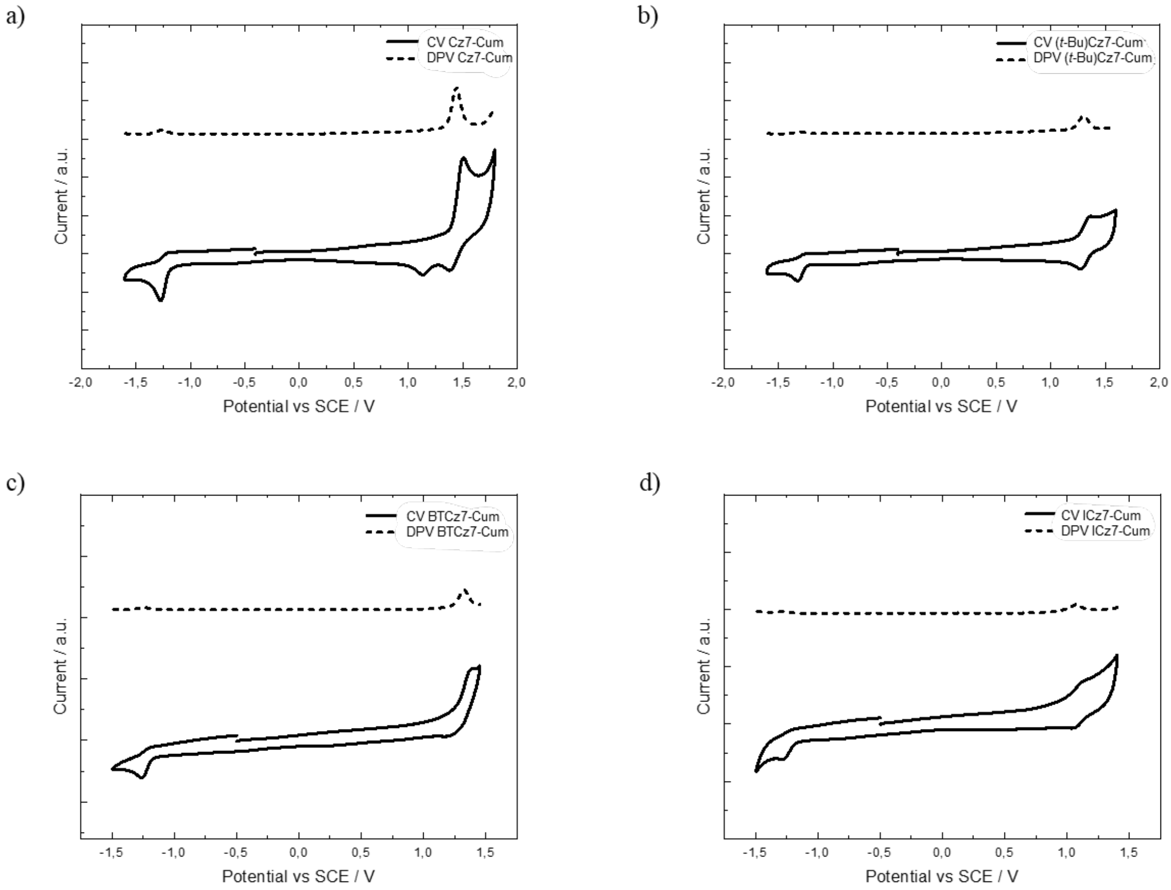

Figure S22. Cyclic voltammetry (solid lines) and differential pulse voltammetry (dashed lines) for a) **Cz7-Cum,** b) **(*t*-Bu)Cz7-Cum**, c) **BTCz7-Cum,** and d) **ICz7**in degassed DCM solution containing [^{n}Bu$_4$N]PF$_6$ as the supporting electrolyte and using Fc/Fc$^+$ as an external standard (Fc/Fc$^+$ = 0.46 eV vs SCE, scan rate = 100 mV s^{-1}).[23]

Table S8. Electrochemical properties of **Cz7-Cum**, **(*t*-Bu)Cz7-Cum**, **BTCz7-Cum** and **ICz7-Cum**.

Compound	E_{ox} ^{a}/ V	E_{red} ^{a}/ V	HOMO^b / eV	LUMO^b / eV	ΔE$_{H-L}$^{c} / eV
**Cz7-Cum**	1.44	- 1.26	-5.79	-3.08	2.71
**(*t*-Bu)Cz7-Cum**	1.31	- 1.31	-5.64	-3.03	2.61
**BTCz7-Cum**	1.32	- 1.25	-5.66	-3.09	2.57
**ICz7-Cum**	1.07	- 1.29	-5.41	-3.06	2.35

^{a}In degassed DCM containing 0.1 M [nBu$_4$N]PF$_6$ as supporting electrolyte and ferrocene/ferrocenium (Fc/Fc$^+$) as the internal reference (0.4 V vs SCE).[25] ^{b}HOMO and LUMO energies were estimated using the equation $E_{HOMO/LUMO} = -(E_{ox/red} + 4.8)$ eV,[26] where E_{ox} and E_{red} are the anodic and cathodic peak potentials, respectively calculated from DPV related to Fc/Fc$^+$. $^c E_{H\text{-}L} = |E_{HOMO}\text{-}E_{LUMO}|$.

Photophysical characterization

Solution-state photophysical study

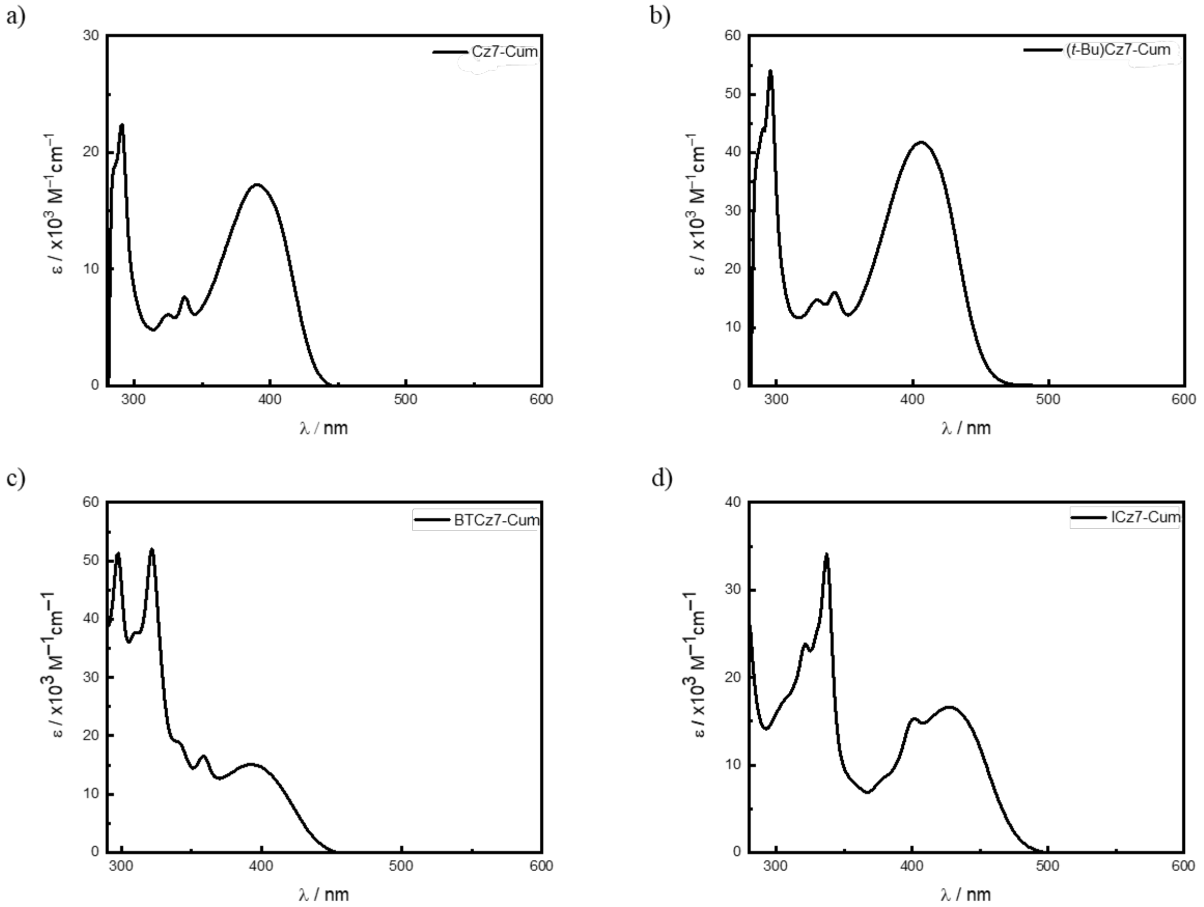

Figure S23. Molar absorptivity for a) **Cz7-Cum,** b) **(*t*-Bu)Cz7-Cum**, c) **BTCz7-Cum,** and d) **ICz7-Cum** in optically dilute toluene solutions (≈10^{-5} M).

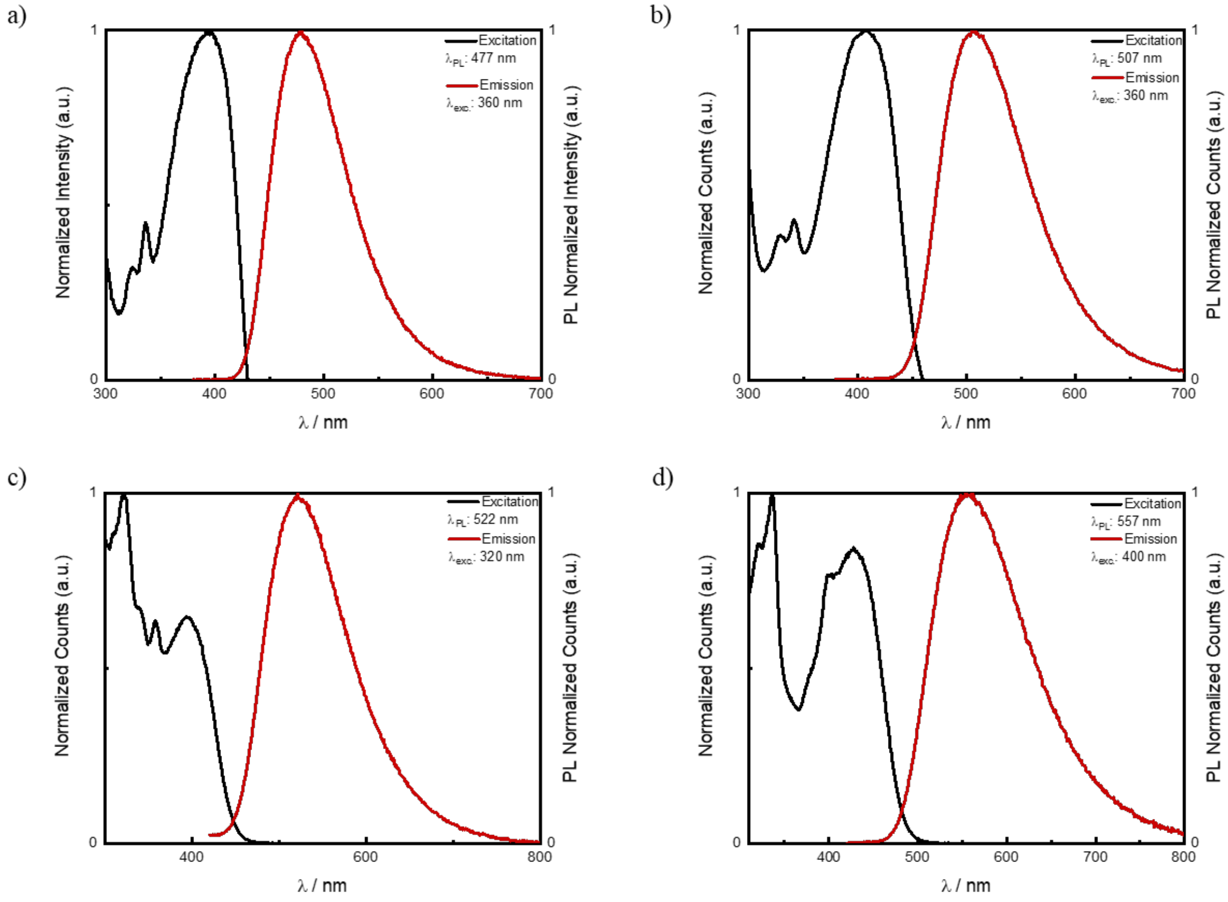

Figure S24. Normalized emission and excitation of a) **Cz7-Cum,** b) **(*t*-Bu)Cz7-Cum**, c) **BTCz7-Cum,** and d) **ICz7-Cum** in optically dilute toluene solutions (≈10^{-5} M).

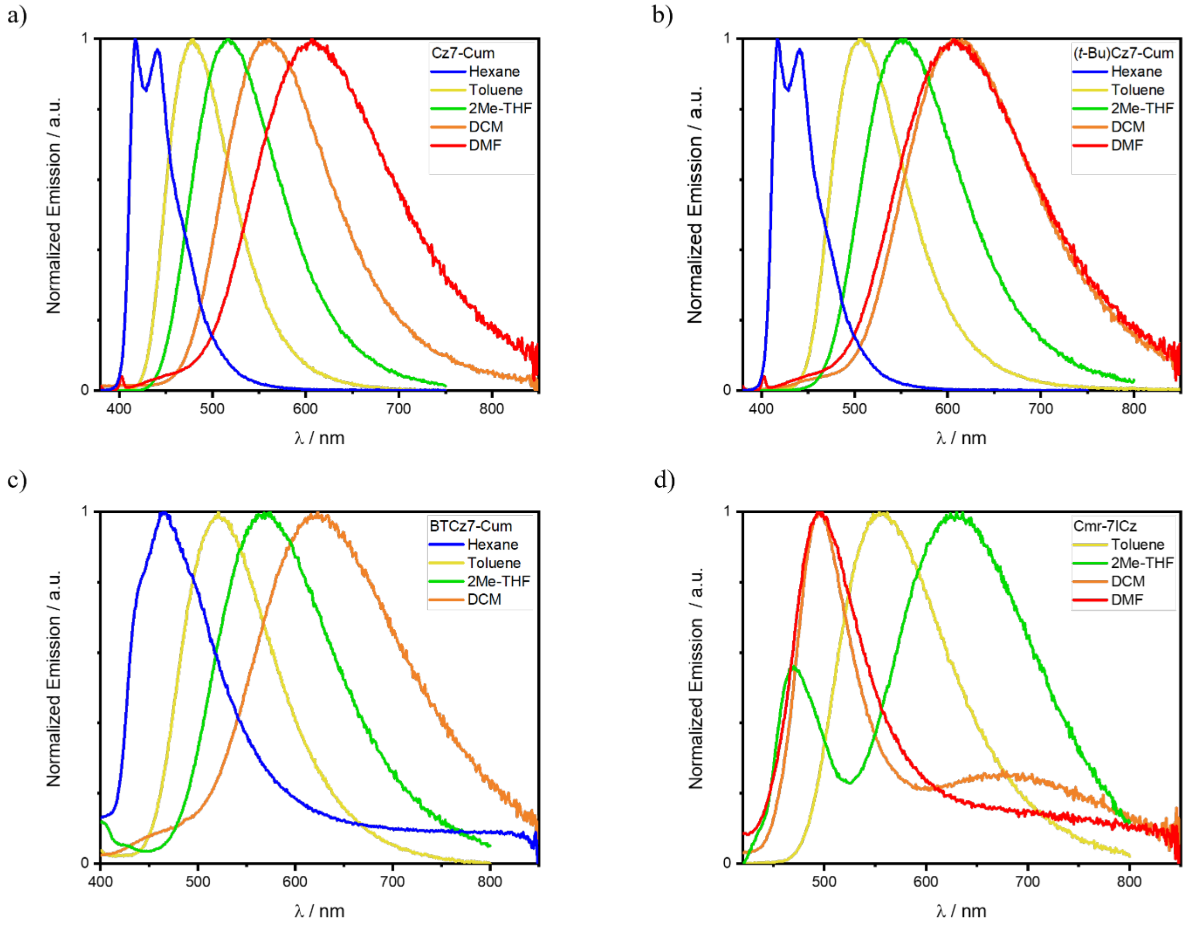

Figure S25. PL spectra in different solvents of a) **Cz7-Cum** (λ_{exc} = 360 nm), b) **(*t*-Bu)Cz7-Cum** (λ_{exc} = 360 nm), c) **BTCz7-Cum** (λ_{exc} = 320 nm), d) **ICz7-Cum** (λ_{exc} = 400). PL spectra of **BTCz7-Cum** in DMF and **ICz7-Cum** in hexane are not shown due to limited solubility of the emitters in these solvents.

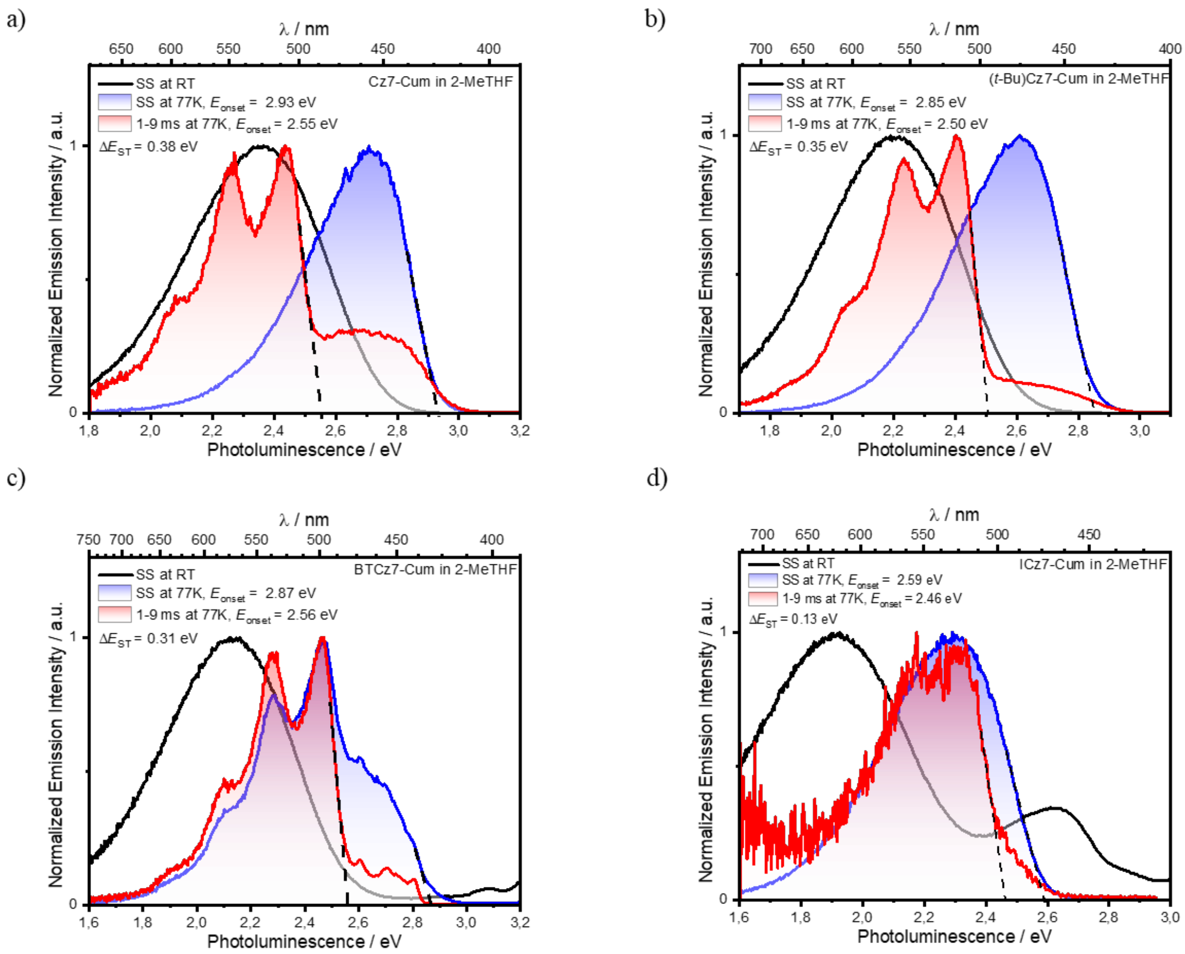

Figure S26. SS PL spectra at RT and at 77K, along with phosphorescence spectra (time-gated window: 1-10 ms) of a) **Cz7-Cum** (λ_{exc} = 360 nm), b) ***(t*-Bu)Cz7-Cum** (λ_{exc} = 360 nm), c) **BTCz7-Cum** (λ_{exc} = 320 nm), d) **ICz7-Cum** (λ_{exc} = 400 nm) in optically dilute 2-MeTHF solutions (≈10^{-5} M).

Table S9. Photophysical properties of coumarin derivatives Cum-7Cz, Cum-7(*t*-Bu)Cz, Cum-7BTCz, Cum-7ICz in dilute toluene solution.

**Compound**	**λ_{PL} / nm [(a)]**	**τ_p / ns [(b)]**	**S_1 / eV [(c)]**	**T_1 / eV [(c)]**	**ΔE_{ST} / eV [(d)]**	**Φ_{PL} / % air [(a) (e)]**
Cum-7Cz	477	5.8	2.93	2.55	0.38	52
Cum-7(*t*-Bu)Cz	500	7.5	2.85	2.50	0.35	55
Cum-7BTCz	522	3.3	2.87	2.56	0.31	31
Cum-7ICz	557	5.2	2.59	2.46	0.13	25

[(a)] In dilute toluene at 300 K (10^{-5} M, λ_{exc} = 360 nm (**Cum-7Cz** and **Cum-7(*t*-Bu)Cz**), 320 nm (**Cum-7BTCz**) and 400 nm (**Cum-7ICz**).

[(b)] In degassed dilute toluene solution (10^{-5} M). τ_p measured by TCSPC λ_{exc} = 375 nm.

[(c)] In 2-MeTHF at 77 K, λ_{exc} = 360 nm (**Cum-7Cz** and **Cum-7(*t*-Bu)Cz**), 320 nm (**Cum-7BTCz**) and 400 nm (**Cum-7ICz**).

[(d)] Determined from the onsets of the steady-state PL and phosphorescence spectra in 2-MeTHF at 77 K, λ_{exc} = 360 nm (**Cum-7Cz** and **Cum-7(*t*-Bu)Cz**), 320 nm (**Cum-7BTCz**) and 400 nm (**Cum-7ICz**).

[(e)] Determined using an integrating sphere.

Polymethylmethacrylate (PMMA) Photoluminescence Study

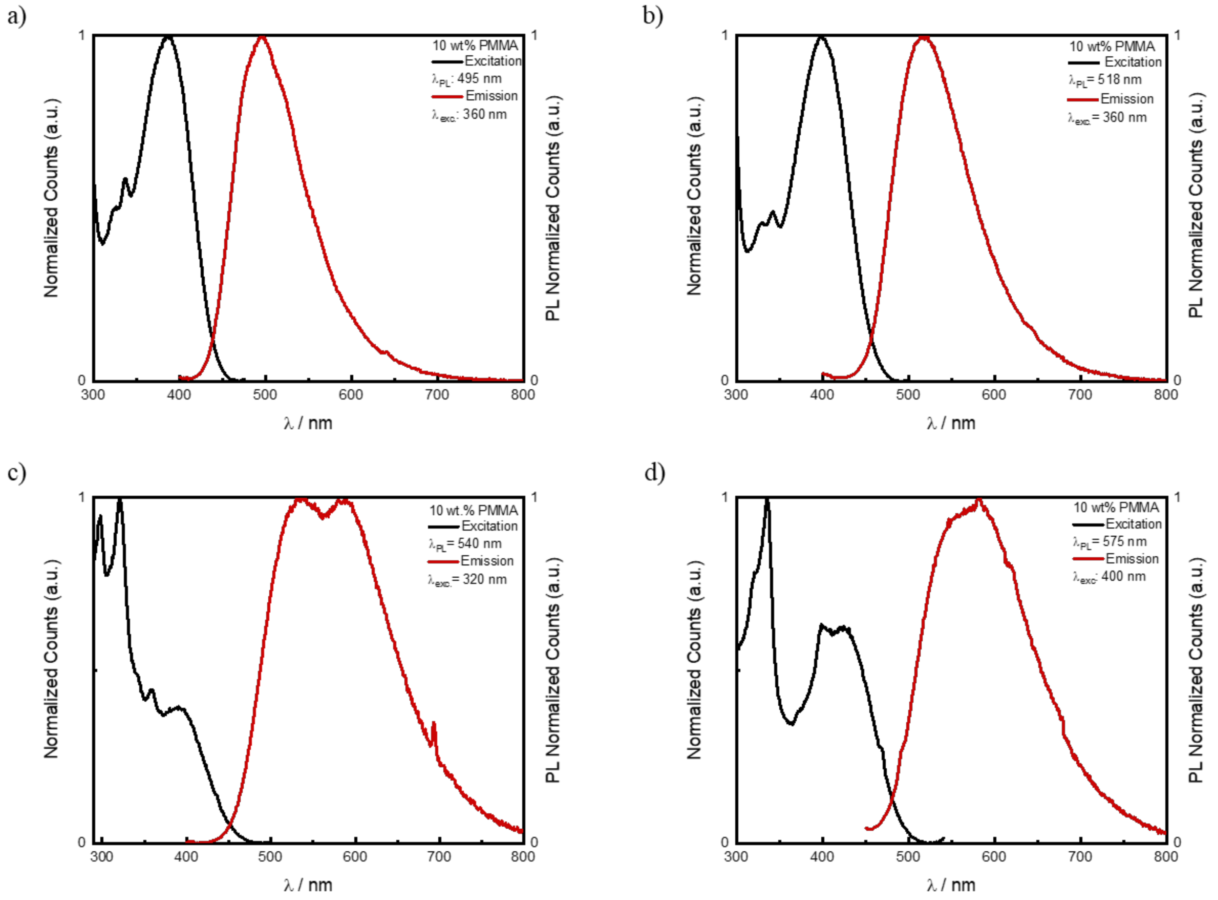

Figure S27. Normalized emission and excitation of a) **Cz7-Cum,** b) **(*t*-Bu)Cz7-Cum**, c) **BTCz7-Cum,** and d) **ICz7-Cum** in 10 wt% films in PMMA.

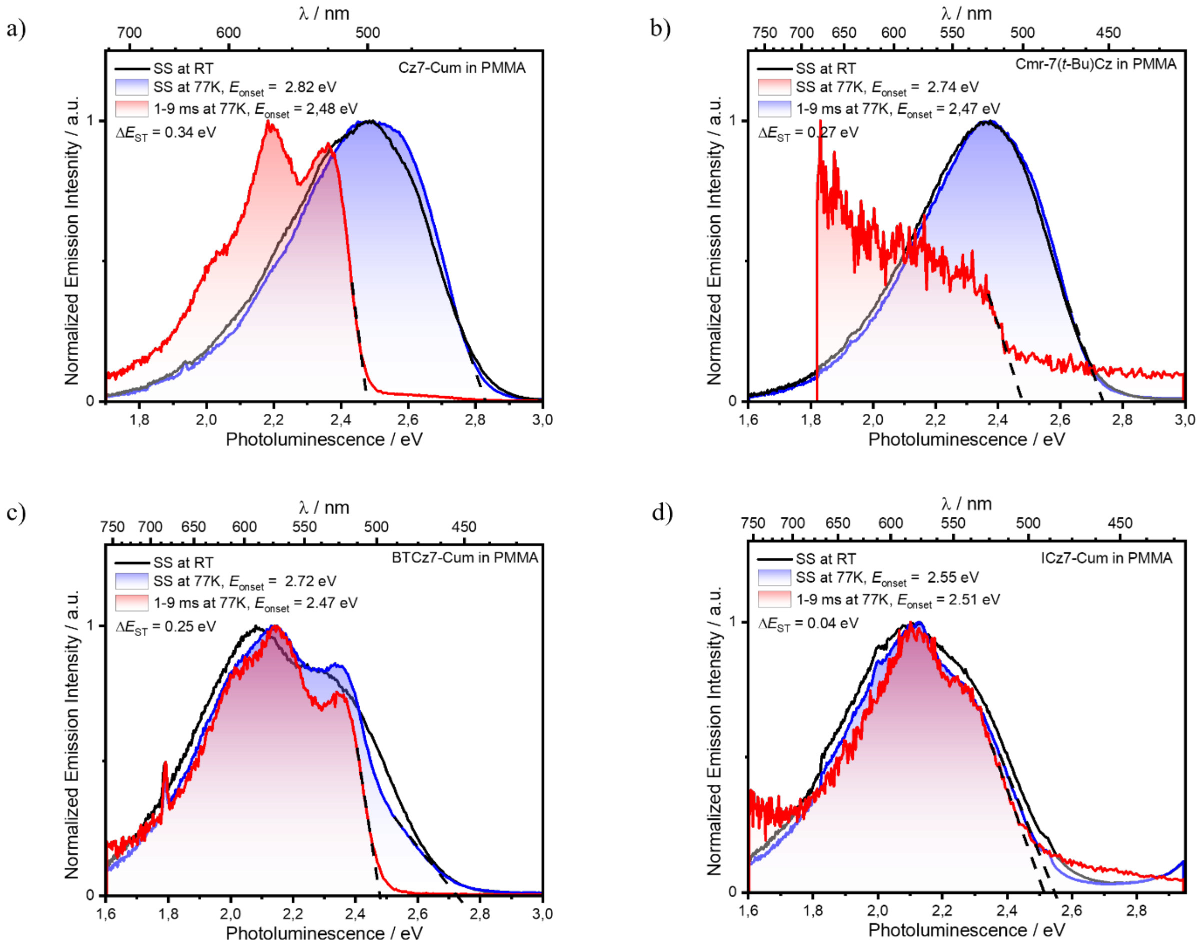

Figure S28. SS PL spectra at RT and at 77 K, along with phosphorescence spectra (time-gated window: 1-10 ms) of a) **Cz7-Cum** (λ_{exc} = 360 nm), b) **(*t*-Bu)Cz7-Cum** (λ_{exc} = 360 nm), c) **BTCz7-Cum** (λ_{exc} = 320 nm), d) **ICz7-Cum** (λ_{exc} = 400 nm) in 10 wt% in PMMA.

Polymers investigation

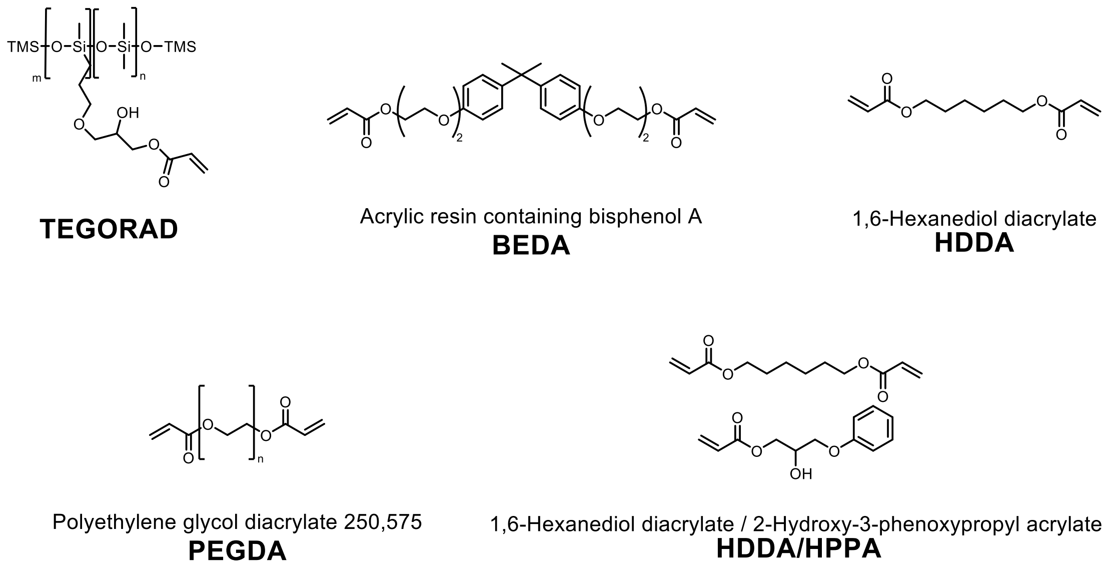

Figure S29. Polymeric resins investigated for the selection of suitable material for three-dimensional sensors.

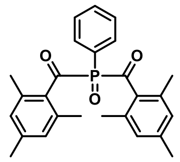

Figure S30. Phenylbis(2,4,6-trimethylbenzoyl)phosphine oxide (**BAPO**) photoinitiator.

Table S10. T_g and water contact angles of monomers.

Monomer	T_g / °C*	Water contact angle / °	Reference
**Pegda 250**	67/30	81	[27-29]
**Pegda 575**	-22/-20	68	[28, 30]
**BEDA**	45	108	[30]
**Tegorad**	-48	115	[31-32]
**HDDA**	52/86	73	[30, 33]

*Measured by DMTA (frequency 1Hz). Different values from different sources could be due to different amounts of photoinitiator used.

The Glass transition temperature of the polymer can give an indication of its rigidity, while the water contact angle of its polarity.

Table S11. Summary of tested 3D printing formulations with varying resin types and **Cz7-Cum** emitter.

Polymer resin type	Polymer resin / g	Photoinitiator / %	Cum-7Cz / wt%	Solvent
**Pegda 250**	1	1	0.2	-
**Pegda 575**	1	1	0.2	-
**Beda**	1	1	0.2	-
**Tegorad**	1	1	0.2	Acetone
**HDDA**	1	1	0.2	-
**HDDA+HPPA**	0.7+0.3	1	0.2	-

Photorheology of Cz7-Cum, (t-Bu)Cz7-Cum, BTCz7-Cum and ICz7-Cum in HDDA

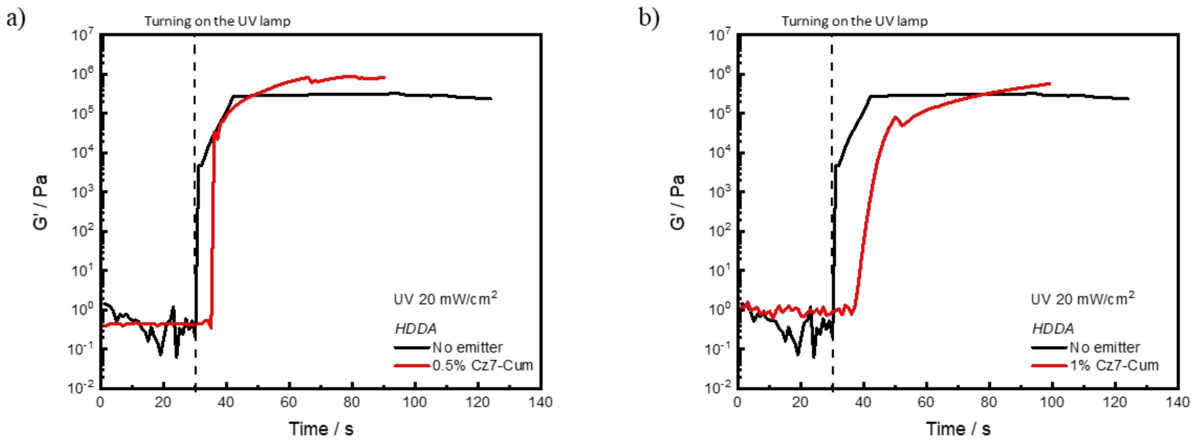

Figure S31. Storage modulus, G' versus irradiation time for HDDA formulations containing **Cz7-Cum** *a*) 0.5 wt%, b) 1 wt%. Film thickness 100 µm, irradiation power 20 mW.

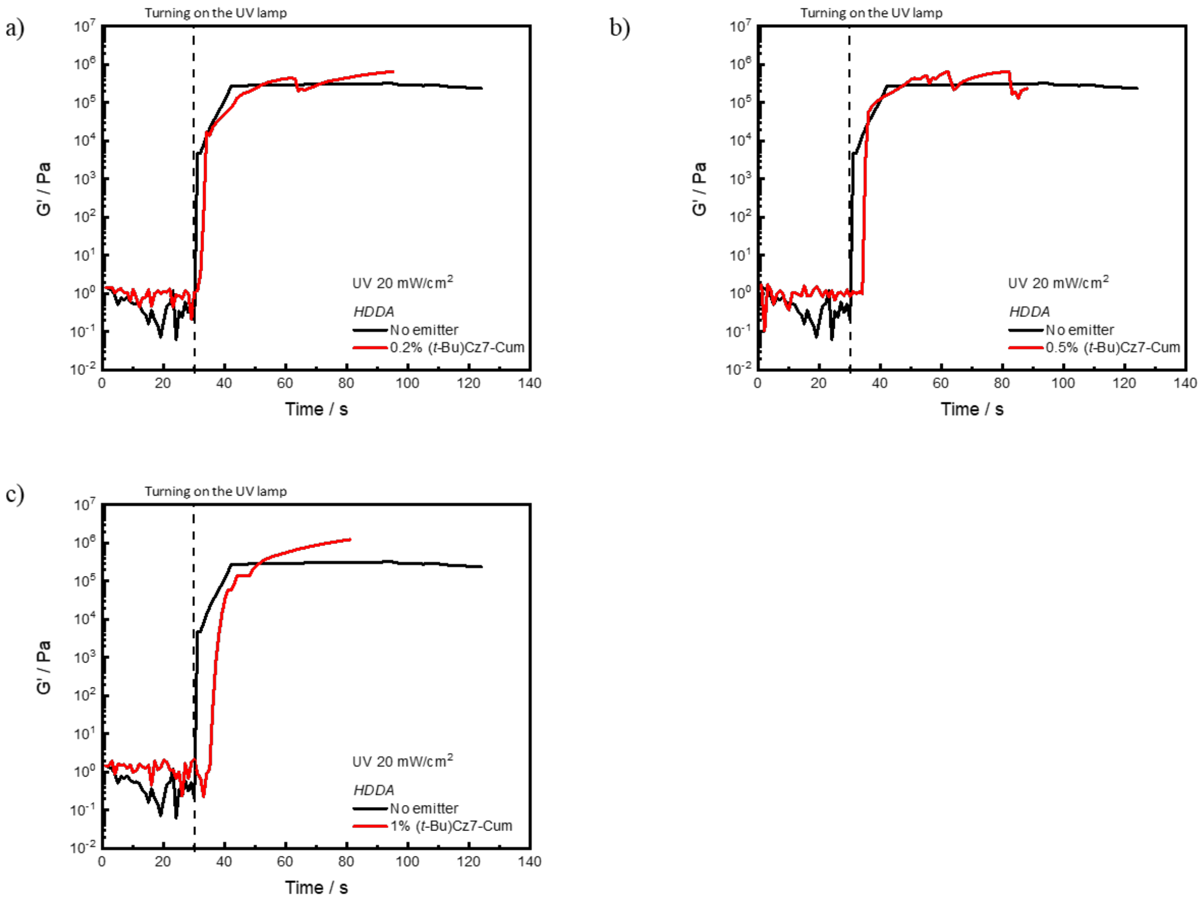

Figure S32. Storage modulus, G' versus irradiation time for HDDA formulations containing **(*t*-Bu)Cz7-Cum** *a*) 0.2 wt%, b) 0.5 wt% and c) 1 wt%. Film thickness 100 µm, irradiation power 20 mW.

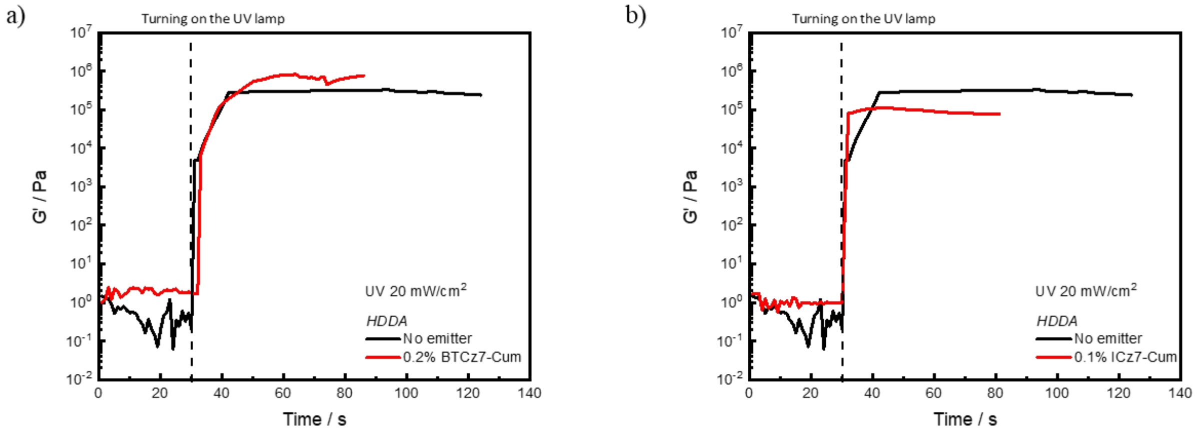

Figure S33. Storage modulus, G' versus irradiation time for HDDA formulations containing a) **BTCz7-Cum** 0.2 wt%, b) **ICz7-Cum** 0.1 wt%. Film thickness 100 µm, irradiation power 20 mW. At higher dye loadings (0.5% and 1 wt% for **BTCz7-Cum** and 0.2%, 0.5% and 1 wt% for **ICz7-Cum**), proper dispersion of the emitter was not achievable, likely due to solubility limits, preventing meaningful photorheological analysis at those concentrations.

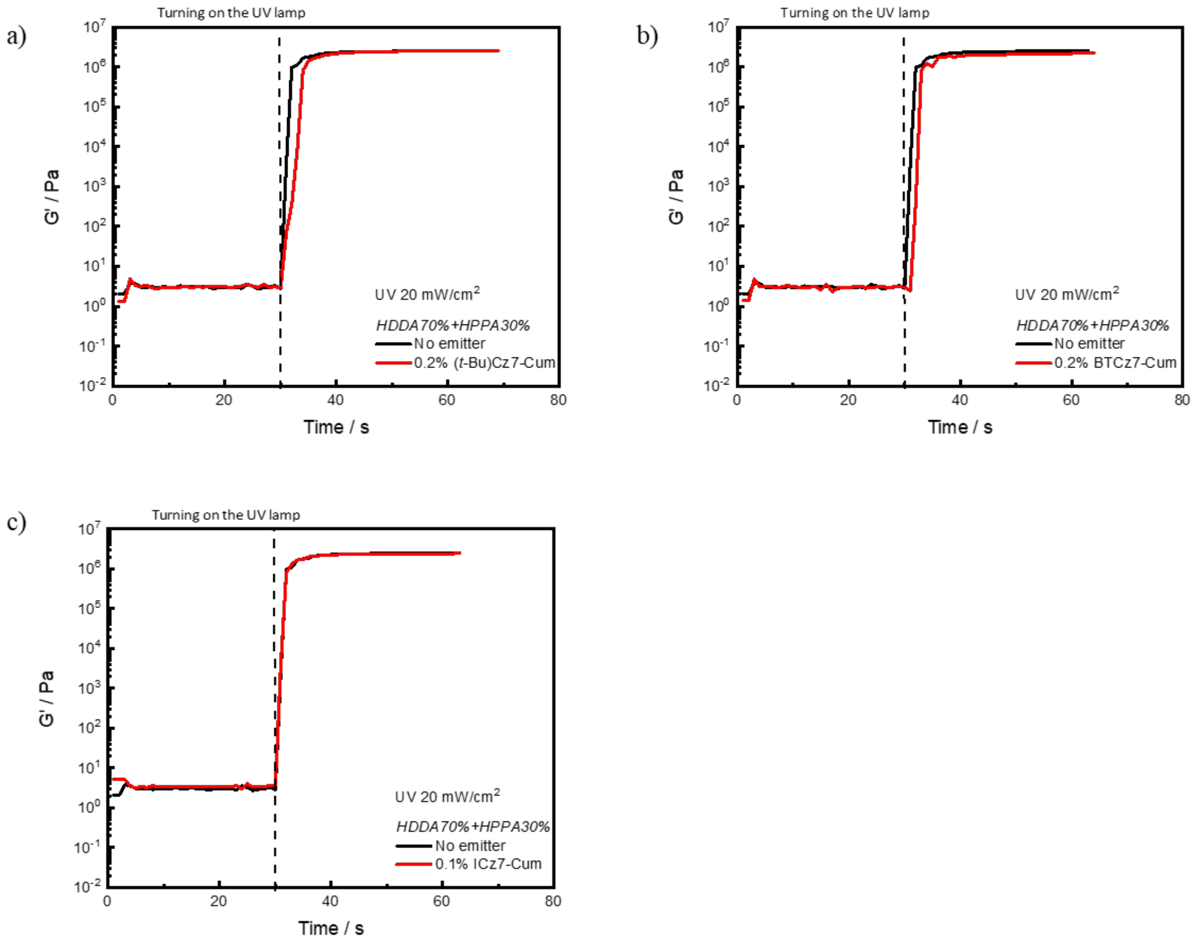

Figure S34. Storage modulus, G' versus irradiation time for HDDA 70% - HPPA 30% formulations containing *a*) 0.2 wt% **(*t*-Bu)Cz7-Cum**, b) 0.2 wt% **BTCz7-Cum,** c) 0.1 wt% **ICz7-Cum**. Film thickness 300 μm, irradiation power 20 mW.

Photophysical Study of Cz7-Cum in the Examined Polymer Resins

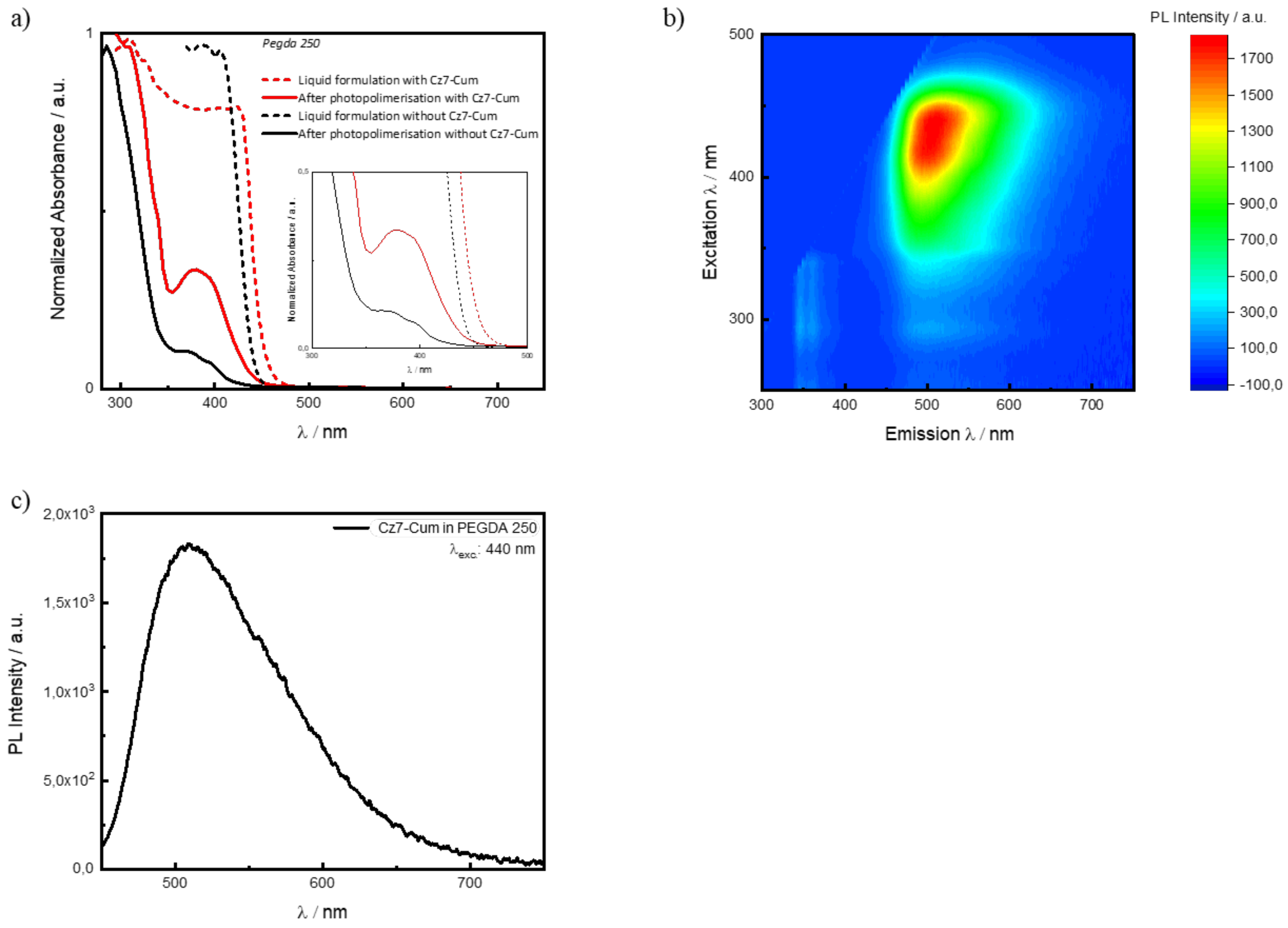

Figure S35. a) UV-Vis absorption before (dashed line) and after (solid line) photopolymerization, without (black) and with (red) 0.2 wt% **Cz7-Cum** in PEGDA 250. b) Excitation-emission map of 3D printed 0.2 wt% **Cz7-Cum** in PEGDA 250. c) Emission of 3D printed 0.2 wt% **Cz7-Cum** in PEGDA 250.

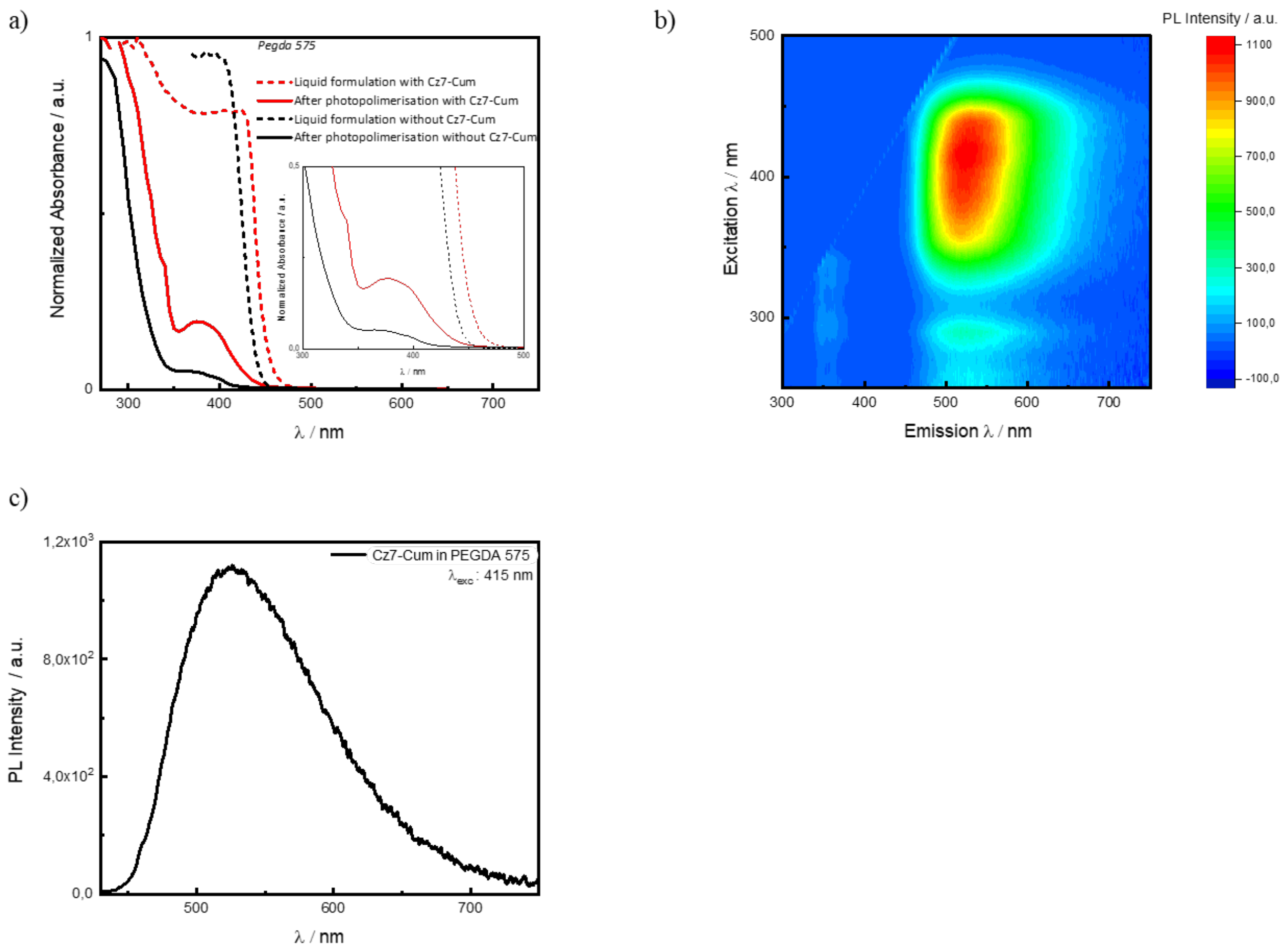

Figure S36. a) UV-Vis absorption before (dashed line) and after (solid line) photopolymerization, without (black) and with (red) 0.2 wt% **Cz7-Cum** in PEGDA 575. b) Excitation-emission map of 3D printed 0.2 wt% **Cz7-Cum** in PEGDA 575. c) Emission of 3D printed 0.2 wt% **Cz7-Cum** in PEGDA 575.

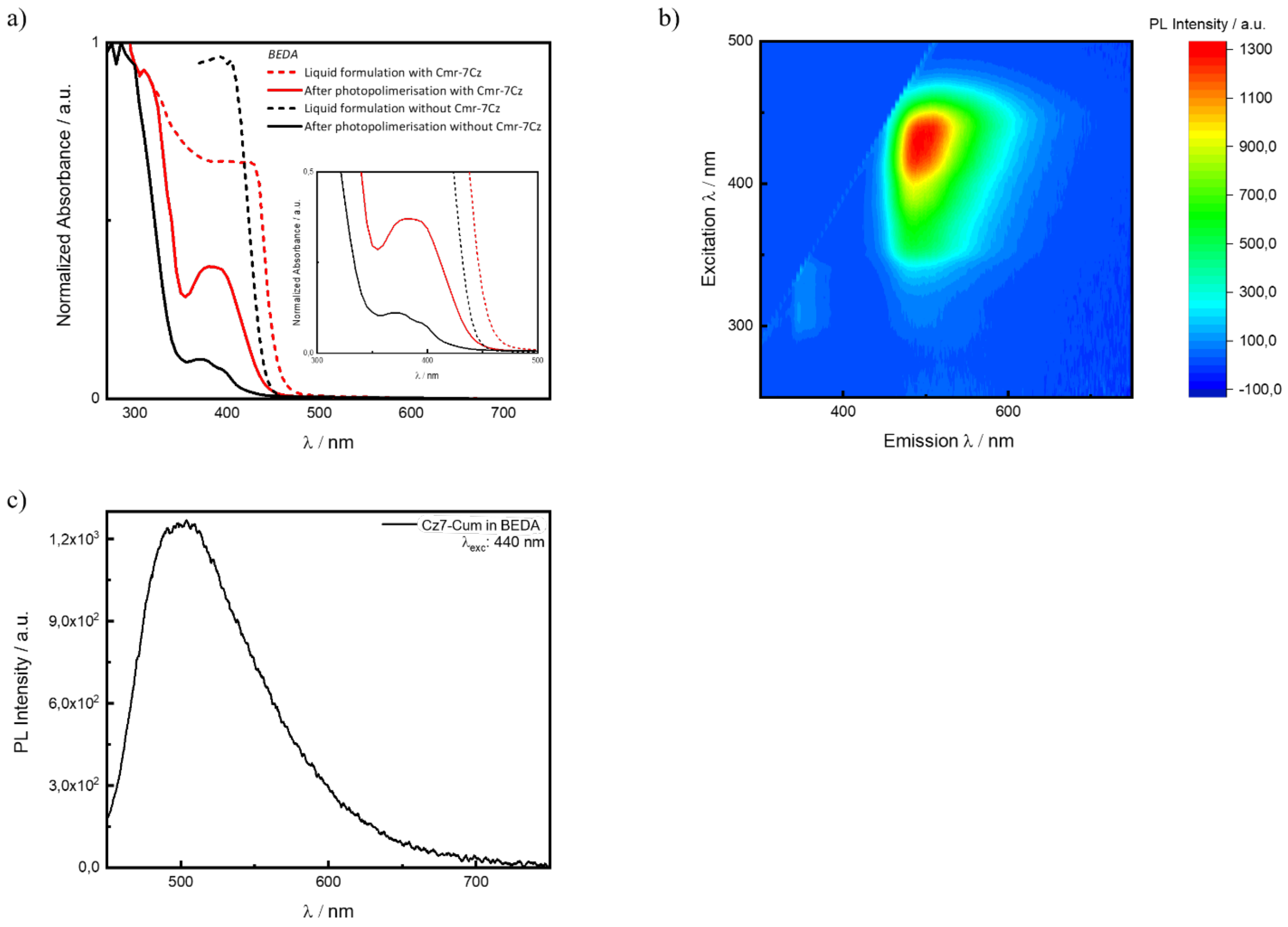

Figure S37. a) UV-Vis absorption before (dashed line) and after (solid line) photopolymerization, without (black) and with (red) 0.2 wt% **Cz7-Cum** in BEDA. b) Excitation-emission map of 3D printed 0.2 wt% **Cz7-Cum** in BEDA. c) Emission of 3D printed 0.2 wt% **Cz7-Cum** in BEDA.

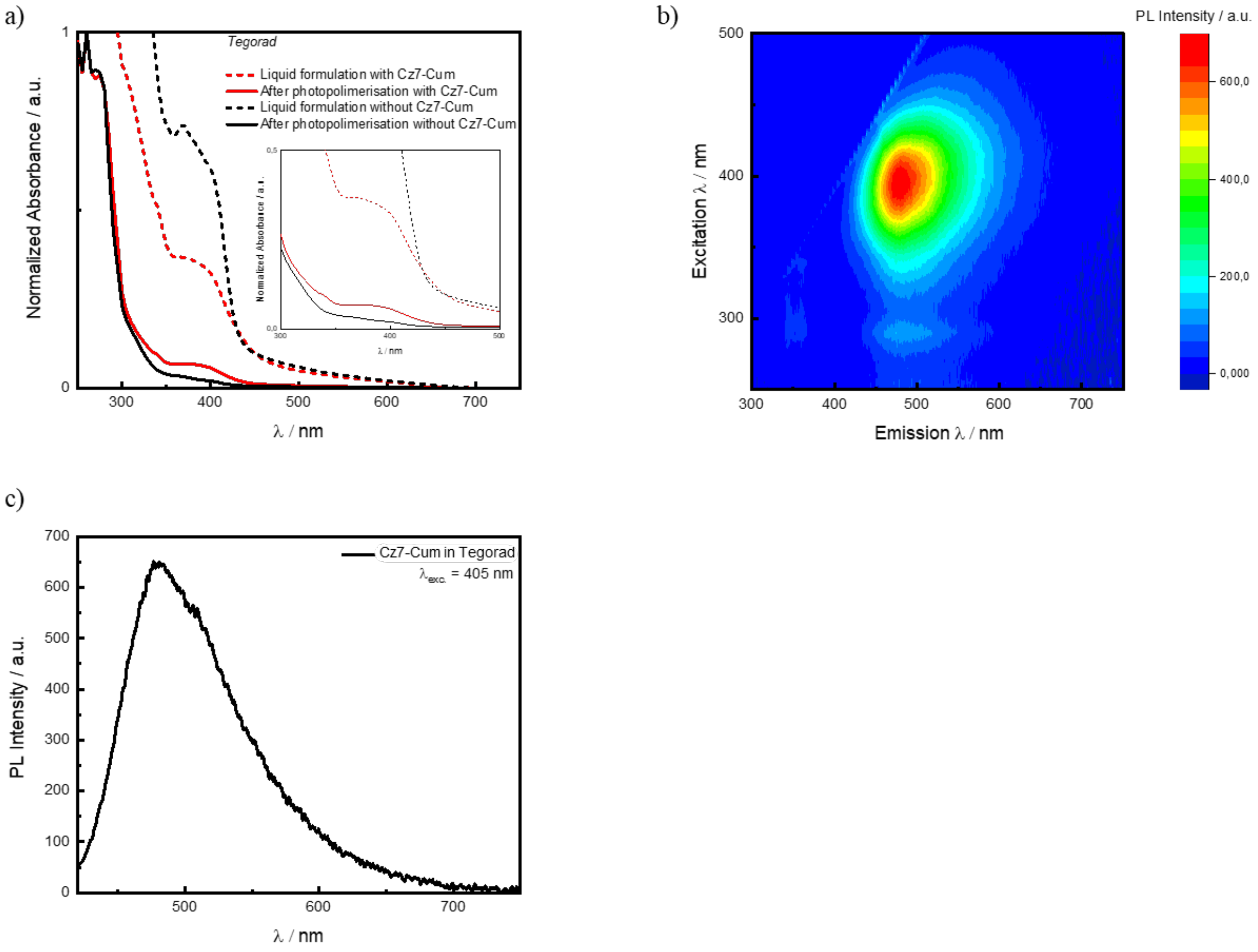

Figure S38. a) UV-Vis absorption before (dashed line) and after (solid line) photopolymerization, without (black) and with (red) 0.2 wt% **Cz7-Cum** in Tegorad. b) Excitation-emission map of 3D printed 0.2 wt% **Cz7-Cum** in tegorad. c) Emission of 3D printed 0.2 wt% **Cz7-Cum** in tegorad.

DLP 3D printing

The photocurable resins were prepared by dissolving the emitter **Cz7-Cum**, **(*t*-Bu)Cz7-Cum**, **BTCz7-Cum** and **ICz7-Cum** at the desired concentration of 0.2 (for the first three) and 0.1 wt% (for **ICz7-Cum**), into a monomeric mixture of 70% HDDA and 30% HPPA. The formulations were homogenized by sonication at room temperature. After complete dissolution, 1 wt% of photoinitiator BAPO (phenylbis(2,4,6-trimethylbenzoyl)phosphine oxide) was added and the formulations were sonicated, protected from the light for 10 minutes. Notably, **ICz7-Cum** required an extended sonication time, likely due to its lower solubility in the resin mixture compared to the other derivatives. The resultant formulation was then placed in the tray of an Anycubic Mono SE 3D printer to obtain the corresponding polymeric material through 3D printing. A rectangular geometry was selected for all prints, with dimensions of 4 mm width × 1.5 cm length × 0.5 mm thickness, chosen to ensure easy handling and suitability for subsequent VOC sensing test. These emitters concentrations were selected based on the resulted previous photophysical and rheological results reported, ensuring optimal printability, structural integrity and sensing performance.

3D printing and scanning of Cz7-Cum in HDDA 70% - HPPA 30%

To fabricate the birdcage-shaped geometry (**Figure S32**), high resolution was required, particularly in the xy-plane and sufficient definition along the z-axis. The printing process was optimized by tuning the parameters reported in **Table S4**. The printed structures were digitized using a 3D scanner, which provided a digital model of the actual structures (**Figure S33**). This model was then compared with the original CAD design to assess the quality of reproduction. The comparison revealed the printing fidelity, highlighting the degree to which the thinner structural elements of the geometry were accurately reproduced with respect to the original model.

Table S12. Printing parameters.

	**Burn-in**	**Range 1**
**Layer thickness / mm**	0.05	0.05
**Light intensity / mW/cm^2**	40	40
**Irradiation time / s**	10	8

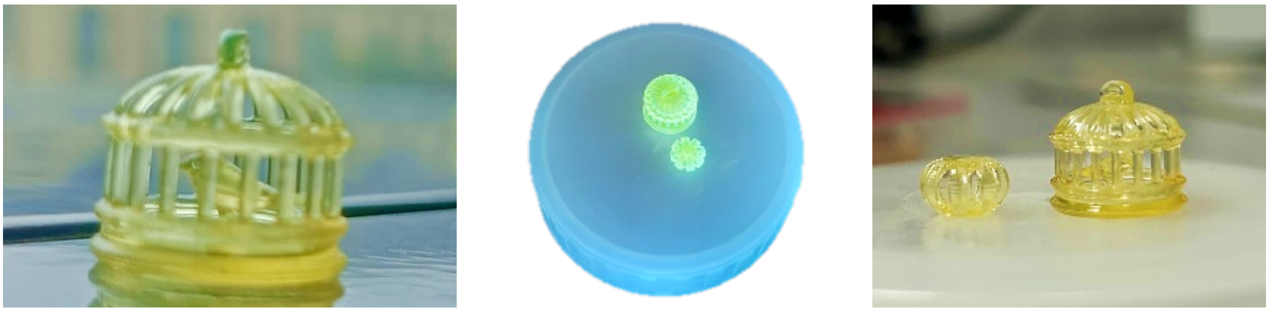

Figure S39. 3D printed birdcage-shaped geometry obtained using 0.2 wt% **Cz7-Cum** in HDDA 70% - HPPA 30%. Images under 365 nm UV excitation (in the centre) and natural daylight (on the sides).

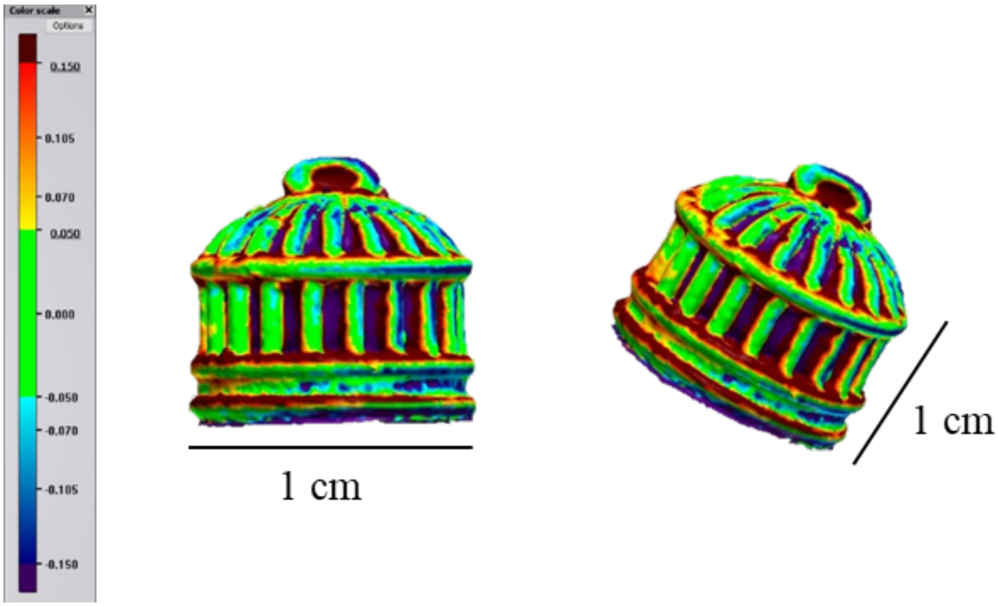

Figure S40. 3D scanning of birdcage-shaped structures printed with **Cz7-Cum**. Colour maps indicate the deviation of the printed samples from the original CAD model, providing a quantitative evaluation of the printing fidelity. Scale bars: 1 cm.

Sensing analysis

Sensing analysis was performed using a custom-built setup at 25°C, to which 500 μL of solvent was added to generate saturated vapours. Fluorescence changes were recorded over 1–2 hours, depending on solvent volatility, alongside UV–Vis absorption, fluorescence lifetime, and ϕ_{PL} measurements to obtain a complete photophysical profile. It is important to note that, since the coumarin-based emitters were physically dispersed within the host matrix rather than covalently incorporated, slight variations in their dispersion between samples prepared on different benches may have introduced minor differences in absorbance and internal quantum efficiency (IQE).

Table S13. Selection of Volatile Organic Compounds used for sensing analysis.

VOC	Boiling Point / °C	Polarity Index	Vapor Pressure 20°C / hPa
**Cyclohexane**	80.7	0.2	104
**Hexane**	69.0	0.0	160
**Toluene**	110.6	2.4	29
**Benzene**	80.1	2.7	101
**Diethyl Ether**	35.0	2.8	587
**DCE**	57.3	3.5	240
**Isopropanol**	82.4	3.9	44
**THF**	66.6	4.0	200
**Ethyl Acetate**	77.0	4.4	97
**Acetone**	56.2	5.1	240
**MeOH**	64.6	5.1	128
**Propionaldehyde**	48.0	-	313

Fluorimetric Analysis

Ethyl 7-(9H-carbazol-9-yl)-2-oxo-2H-chromene-3-carboxylate (Cz7-Cum) spectra

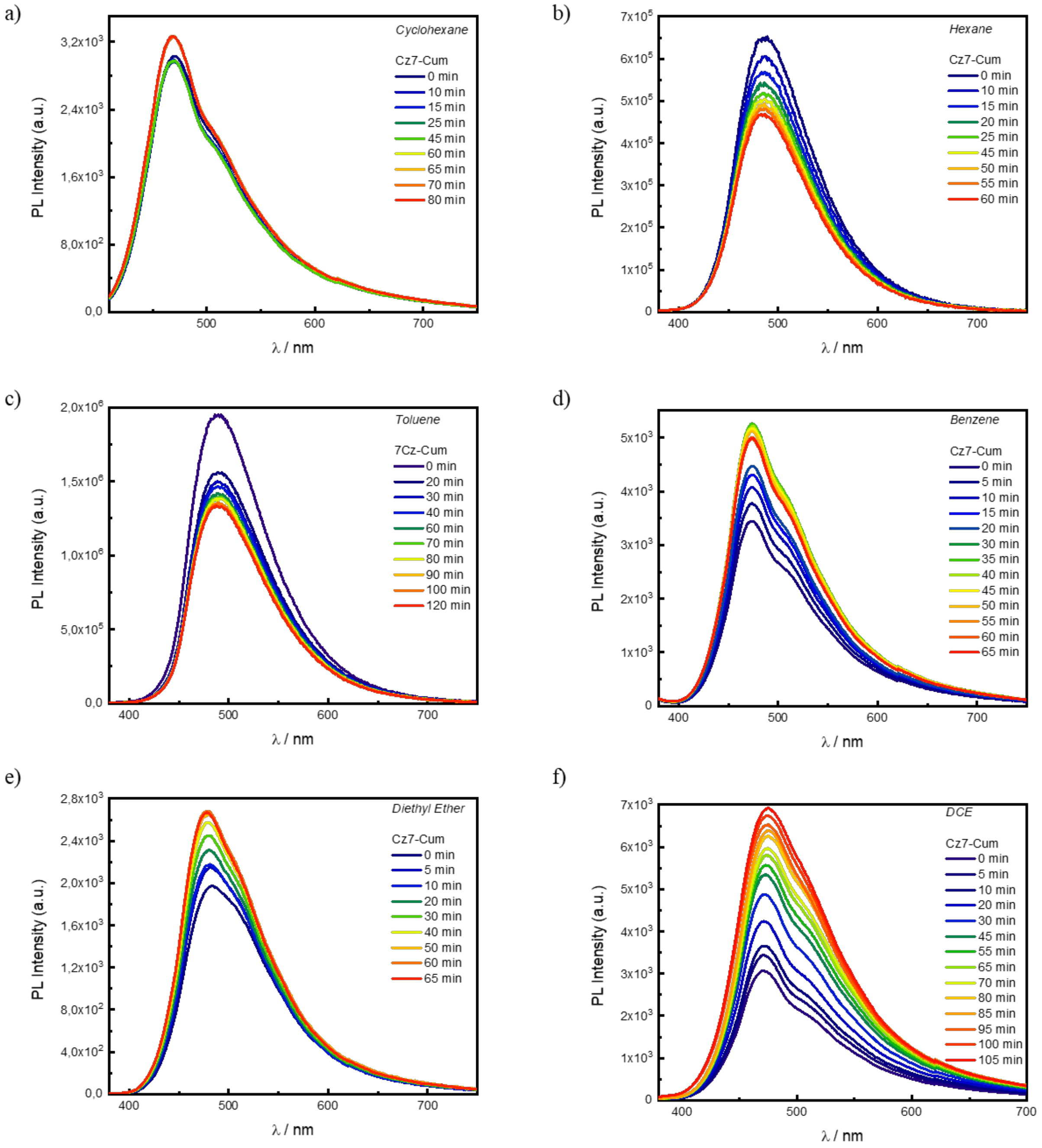

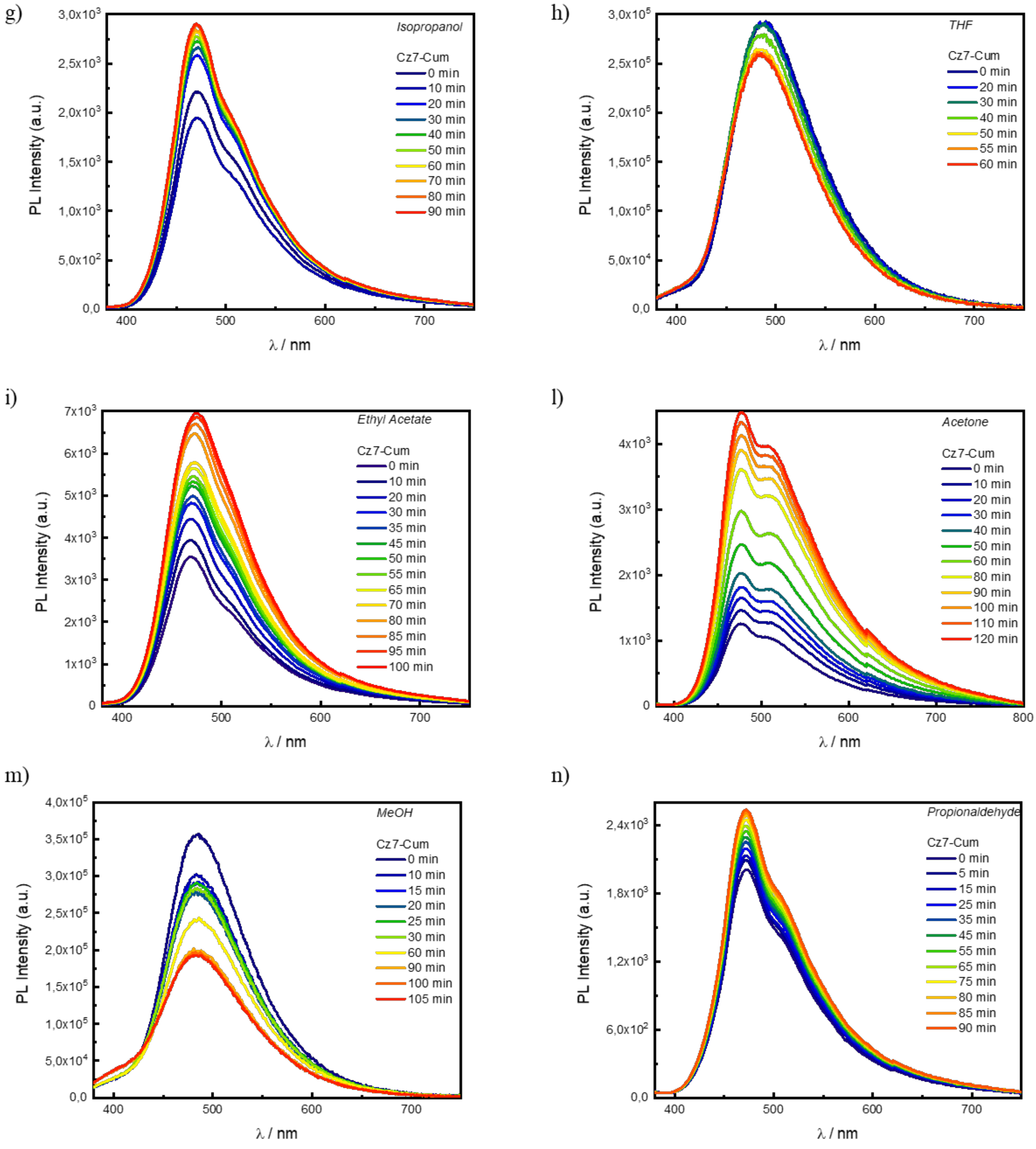

Figure S41. Emission spectra of a film composed of HDDA 70% - HPPA 30%containing 0.2 wt% **Cz7-Cum** (λ_{exc} = 360 nm), recorded over time during exposure to a) cyclohexane, b) hexane, c) toluene, d) benzene, e) diethyl ether, f) DCE, g) isopropanol, h) THF, i) ethyl acetate, l) acetone, m) methanol, n) propionaldehyde vapor.

Ethyl 7-(3,6-di-tert-butyl-9H-carbazol-9-yl)-2-oxo-2H-chromene-3-carboxylate ((*t*-Bu)Cz7-Cum) spectra

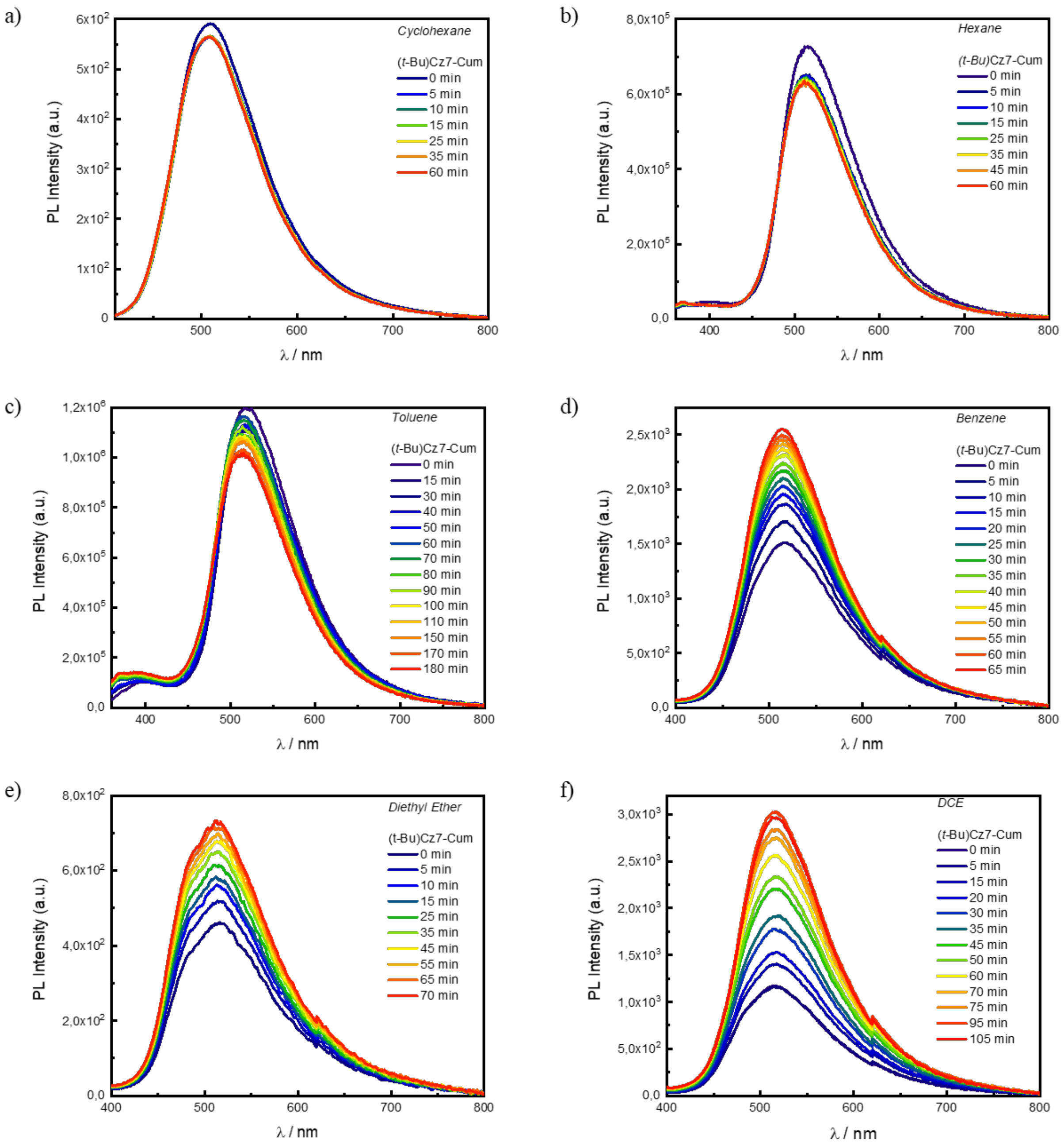

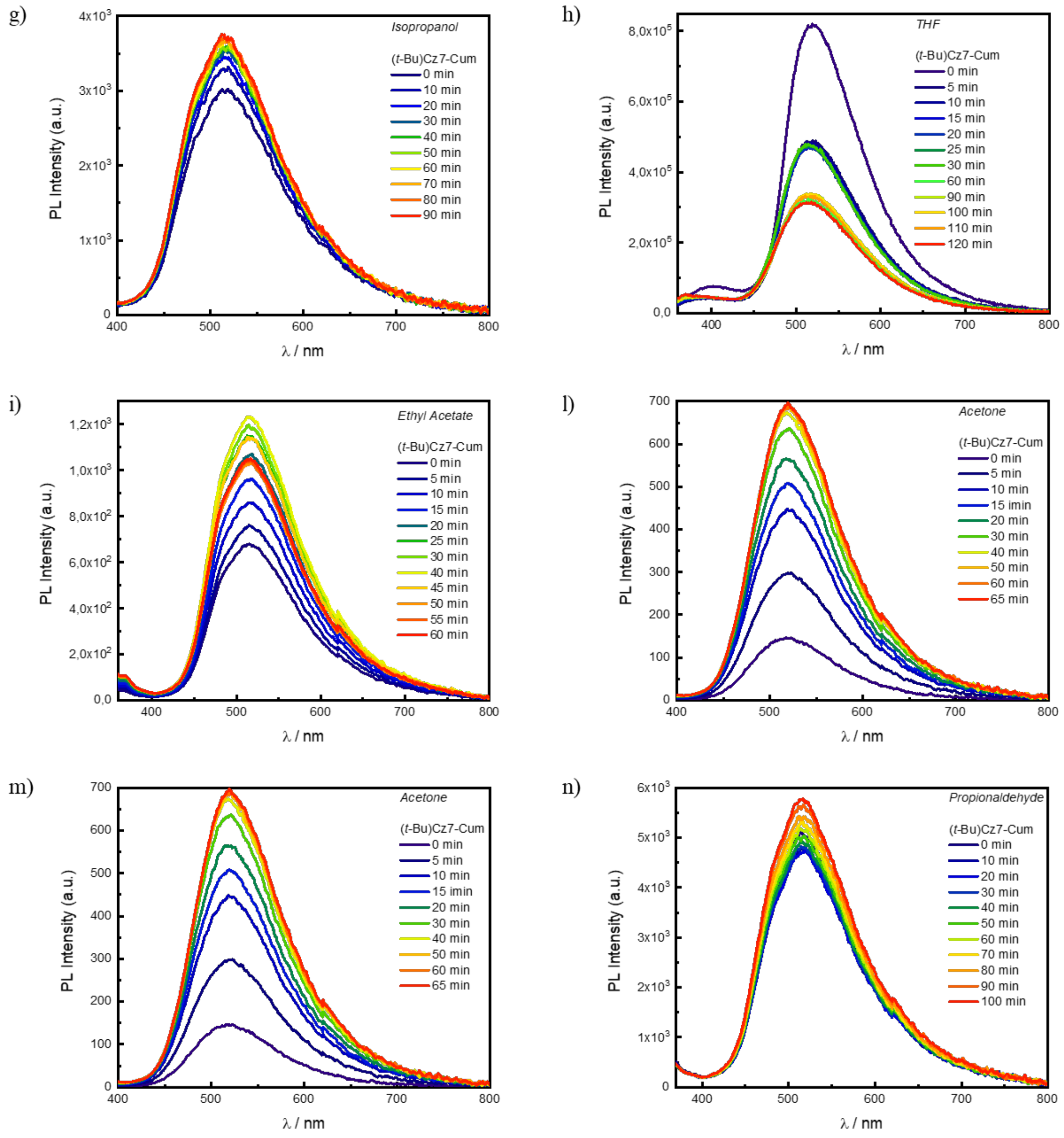

Figure S42. Emission spectra of a film composed of HDDA 70% - HPPA 30% containing 0.2 wt% ***(t*-Bu)Cz7-Cum** (λ_{exc} = 340 nm), recorded over time during exposure to a) cyclohexane, b) hexane, c) toluene, d) benzene, e) diethyl ether, f) DCE, g) isopropanol, h) THF, i) ethyl acetate, l) acetone, m) methanol, n) propionaldehyde vapor.

Ethyl 7-(12H-benzo[4,5]thieno[2,3-a]carbazol-12-yl)-2-oxo-2H-chromene-3-carboxylate (BTCz7-Cum) spectra

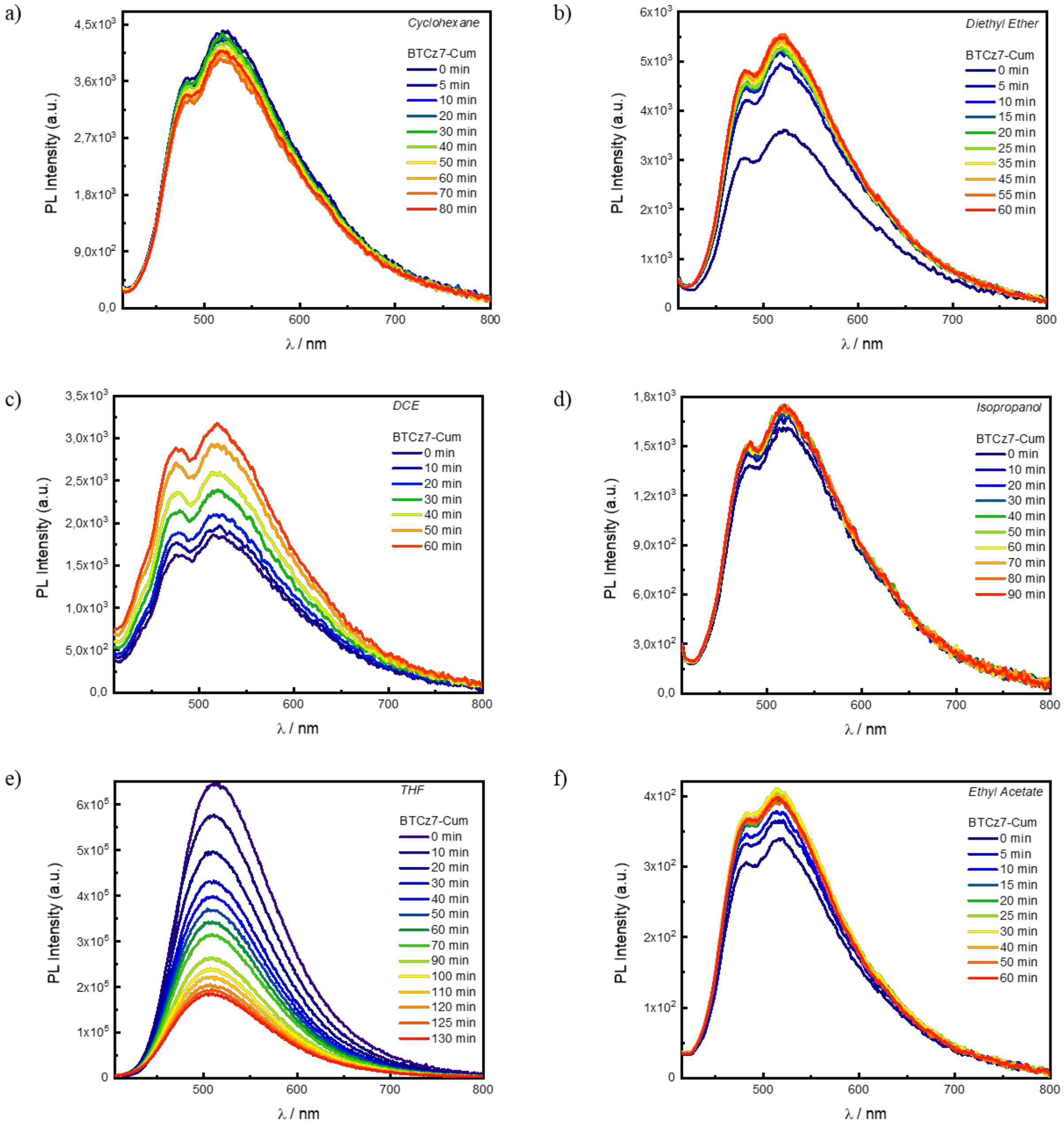

g)

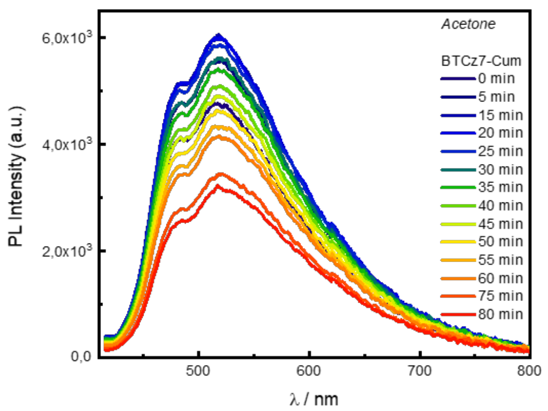

h)

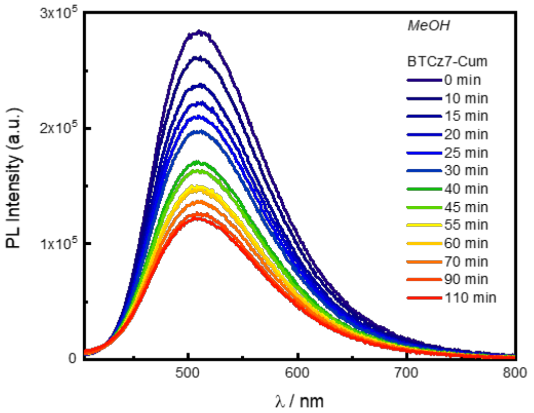

i)

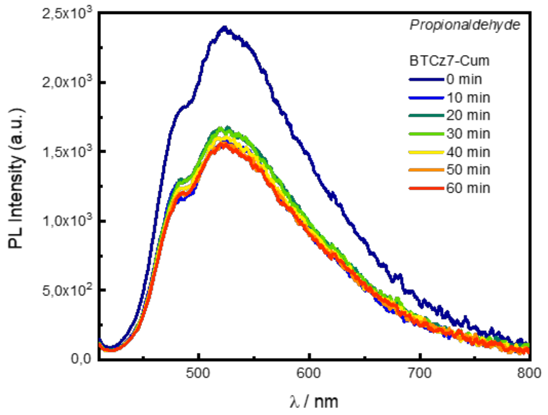

Figure S43. Emission spectra of a film composed of HDDA 70% - HPPA 30% containing 0.2 wt% **BTCz7-Cum** (λ_{exc} = 390 nm), recorded over time during exposure to *a)* cyclohexane, *b)* diethyl ether, *c)* DCE, *d)* isopropanol, *e)* THF, *f)* ethyl acetate, *g)* acetone, *h)* methanol, *i)* propionaldehyde vapor.

Diethyl 7,7'-(indolo[3,2-b]carbazole-5,11-diyl)bis(2-oxo-2H-chromene-3-carboxylate) (ICz7-Cum) spectra

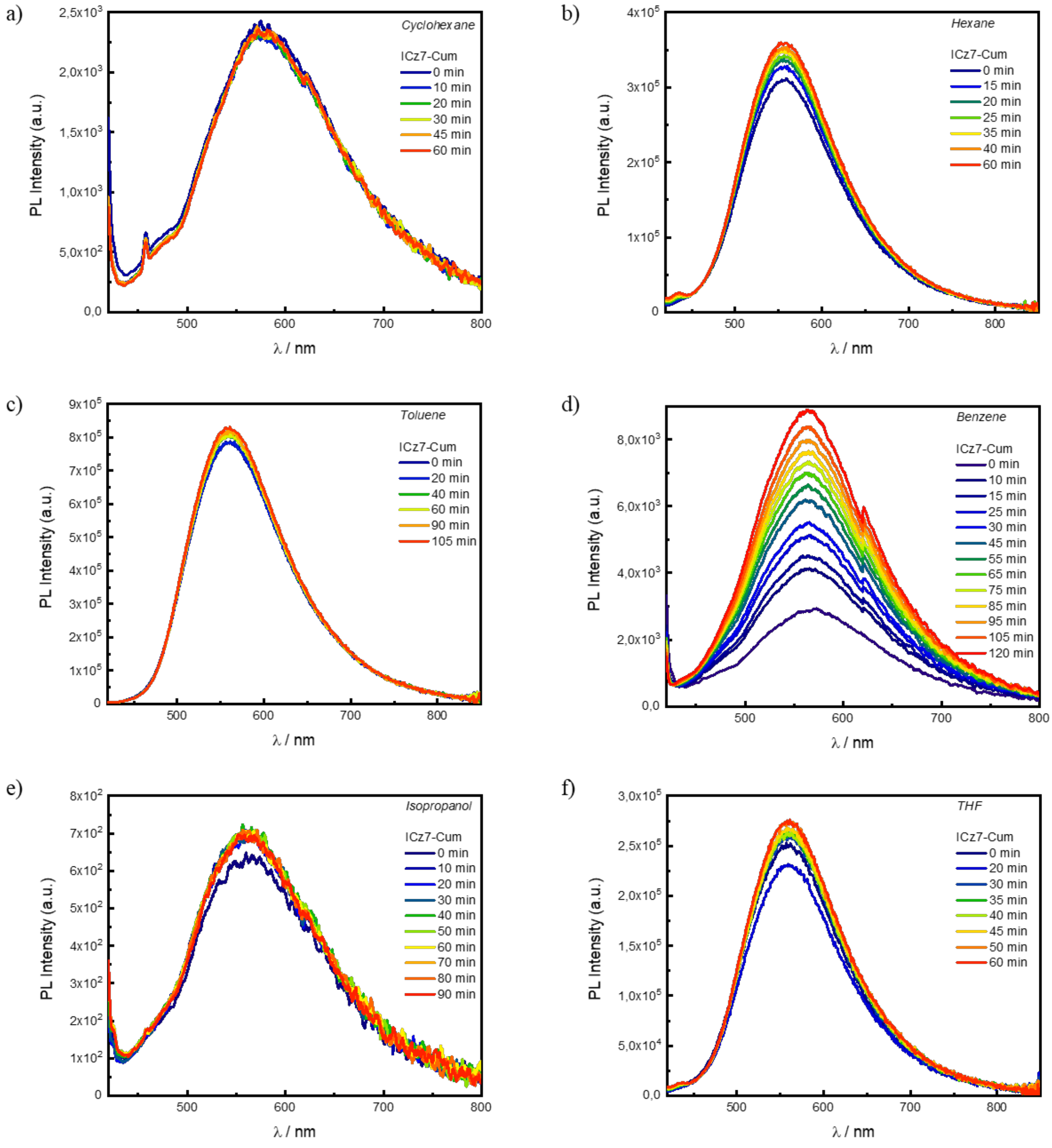

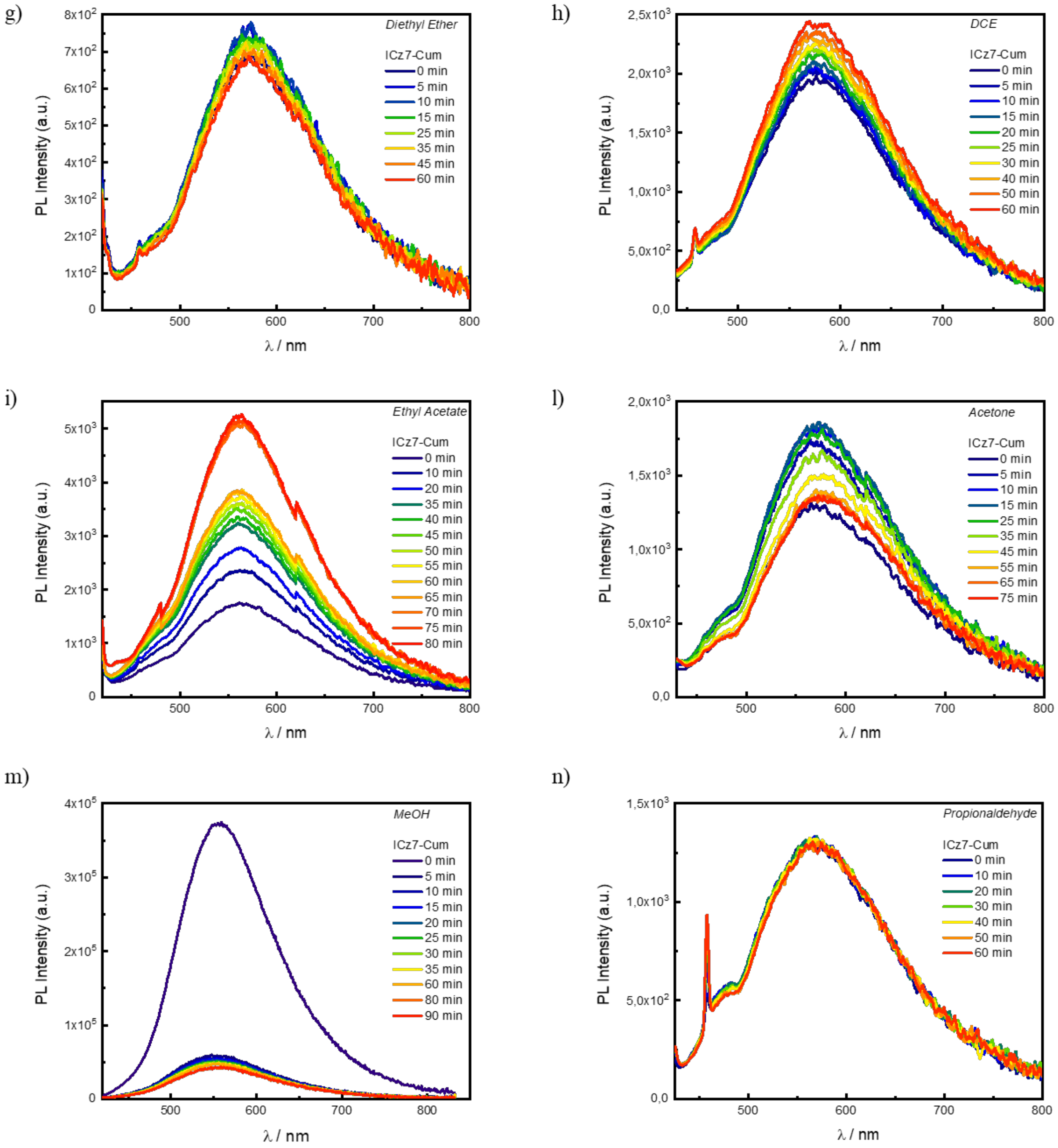

Figure S44. Emission spectra of a film composed of HDDA 70% - HPPA 30% containing 0.1 wt% **ICz7-Cum** (λ_{exc} = 400 nm), recorded over time during exposure to a) cyclohexane, b) hexane, c) toluene, d) benzene, e) diethyl ether, f) DCE, g) isopropanol, h) THF, i) ethyl acetate, l) acetone, m) methanol, n) propionaldehyde vapor. Measurements were carried out under identical conditions.

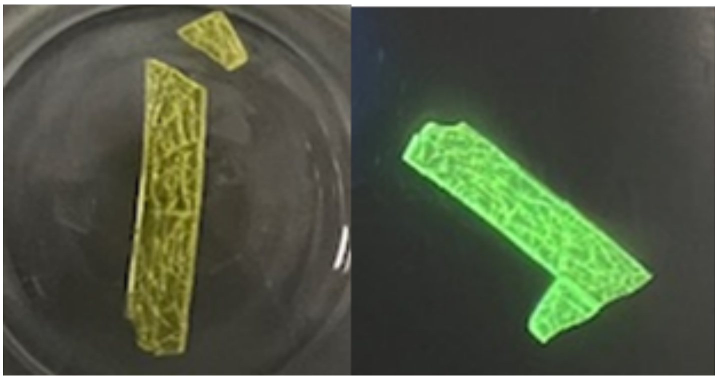

Figure S45. Photographs pf 3D-pinted samples composed of HDDA 70% - HPPA 30% after exposure to dichloromethane vapor. Images show the material under natural daylight (on the left) and 365 nm UV excitation (on the right).

Time-Dependent PL intensity Profile

a)

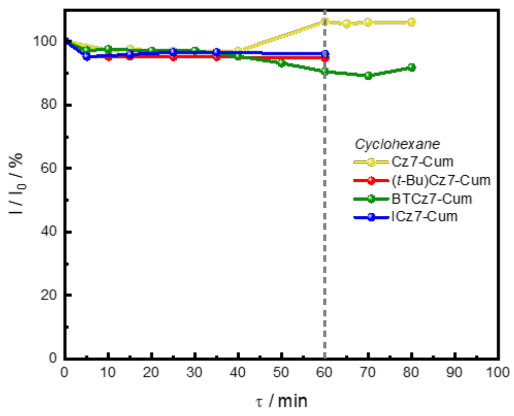

b)

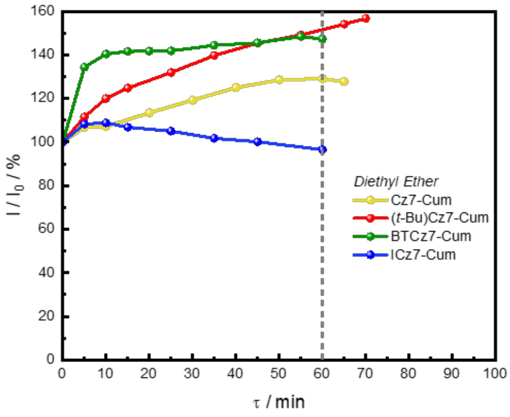

c)

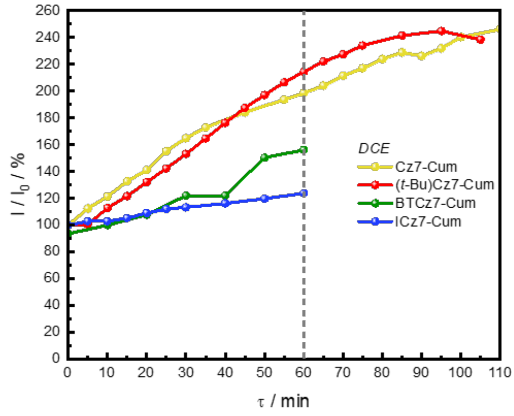

d)

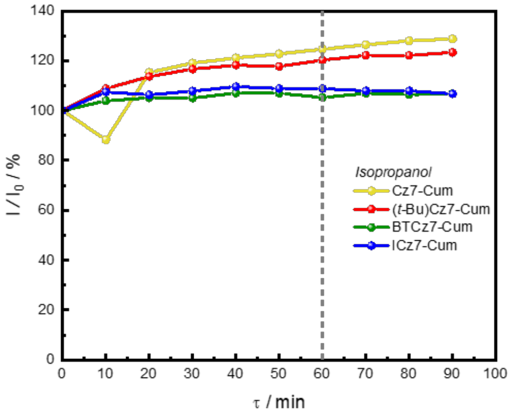

e)

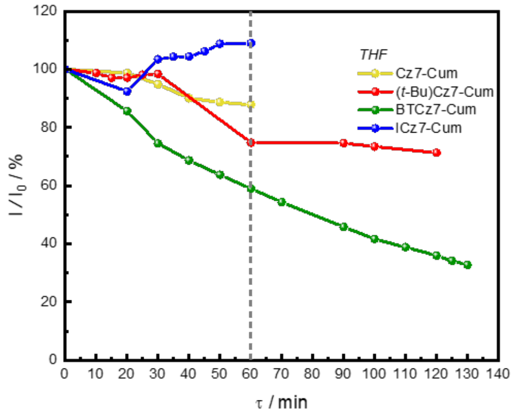

f)

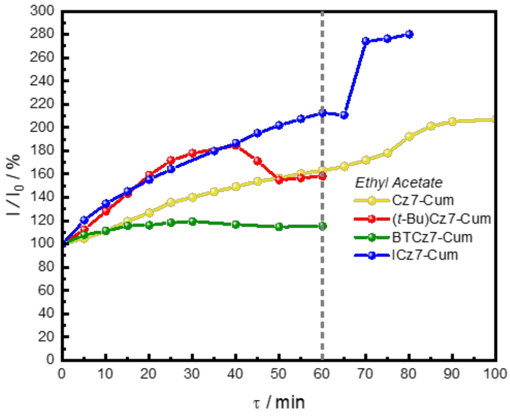

g)

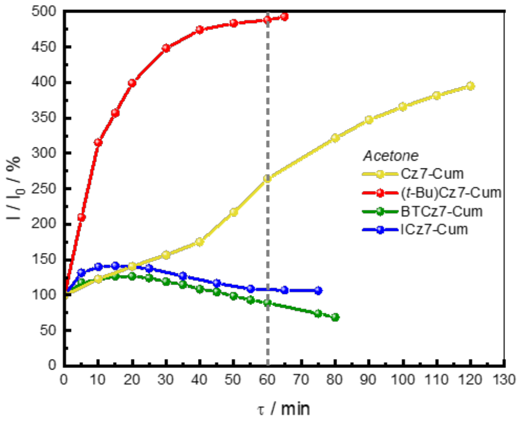

h)

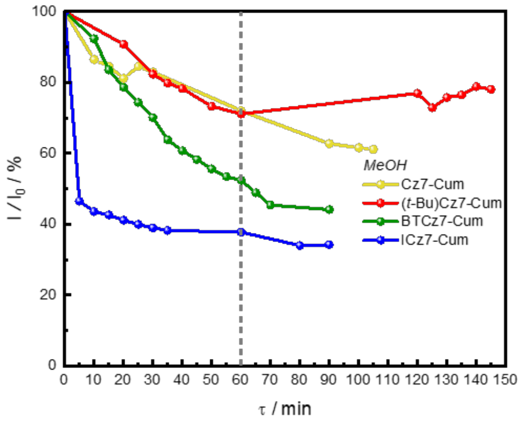

i)

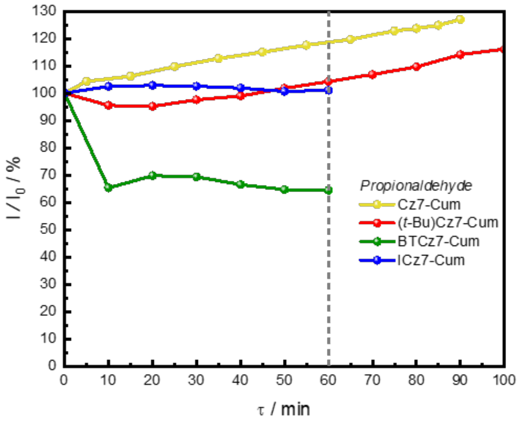

Figure S46. Time-dependent PL intensity of the sensor material composed of HDDA 70% - HPPA 30%, expressed as a percentage relative to its initial value (100 %), upon exposure to a) cyclohexane, b) diethyl ether, c) DCE, d) isopropanol, e) THF, f) ethyl acetate, g) acetone, h) methanol, i) propionaldehyde vapor. Concentration of 0.2 wt% **Cz7-Cum** (λ_{exc} = 360 nm), 0.2 wt% **(*t*-Bu)Cz7-Cum** (λ_{exc} = 340 nm), 0.2 wt% **BTCz7-Cum** (λ_{exc} = 390 nm), 0.1 wt% **ICz7-Cum** (λ_{exc} = 400 nm).

Fluorescence lifetime analysis

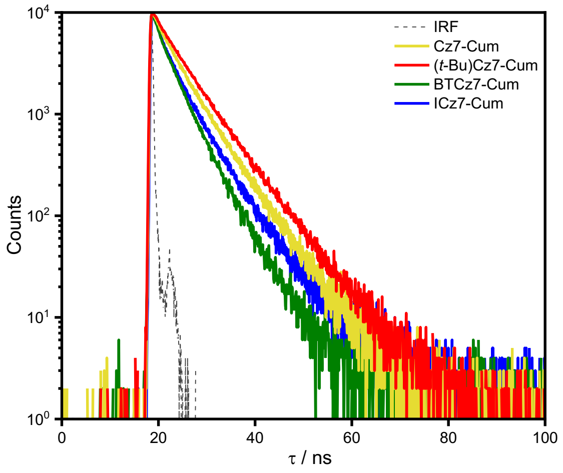

Figure S47. Time-resolved PL decays of the 0.2 wt% emitters (**Cz7-Cum** λ_{PL} = 490 nm, **(*t*-Bu)Cz7-Cum** λ_{PL} = 518 nm, **BTCz7-Cum** λ_{PL} = 510 nm, **ICz7-Cum** λ_{PL} = 562 nm) in HDDA 70% - HPPA 30% 3D printed materials, recorded by TCSPC, λ_{exc} = 375 nm.

Table S14. Photophysical properties in 0.2 wt% (0.1 wt% of **ICz7-Cum**) 3D printed materials in HDDA 70% - HPPA 30%.

Compound	λ_{PL} / nm	$\tau_{avg,p}$ / ns [(a)]	$\tau_{avg,d}$ / µs [(b)]
**Cz7-Cum**	490	5.2	-
**(*t*-Bu)Cz7-Cum**	518	6.0	-
**BTCz7-Cum**	510	3.8	58.2
**ICz7-Cum**	562	4.5	-

[(a)] Prompt lifetime was measured by TCSPC. λ_{exc} = 375 nm.

[(b)] Delayed lifetime was measured by MCS. λ_{exc} = 360 nm (**Cz7-Cum** and (**(*t*-Bu)Cz)7-Cum**), 320 nm (**BTCz7-Cum**) and 400 nm (**ICz7-Cum**).

Table S15. Lifetimes of the samples upon exposure to solvent vapours.

**Cz7-Cum**		**(*t*-Bu)Cz7-Cum**		**ICz7-Cum**	
	**$\tau_{avg,p}$ / ns** [(a)]		**$\tau_{avg,p}$ / ns** [(a)]		**$\tau_{avg,p}$ / ns** [(a)]
**Hexane**	5.2	**Hexane**	5.7	**Hexane**	4.6
**MeOH**	5.0	**MeOH**	5.2	**MeOH**	4.1
**THF**	5.0	**THF**	5.6	**THF**	4.1
**Toluene**	5.0	**Toluene**	5.8	**Toluene**	4.7

**BTCz7-Cum**		
	**$\tau_{avg,p}$ / ns** [(a)]	**$\tau_{avg,d}$ / μs** [(b)]
**Hexane**	4.0	373.0
**MeOH**	10.3	50.4
**THF**	4.0	54.0
**Toluene**	4.5	52.00

[(a)] Prompt lifetime was measured by TCSPC. λ_{exc} = 375 nm.

[(b)] Delayed lifetime was measured by MCS. λ_{exc} = nm (**BTCz7-Cum**).

Ethyl 7-(9H-carbazol-9-yl)-2-oxo-2H-chromene-3-carboxylate (Cz7-Cum)

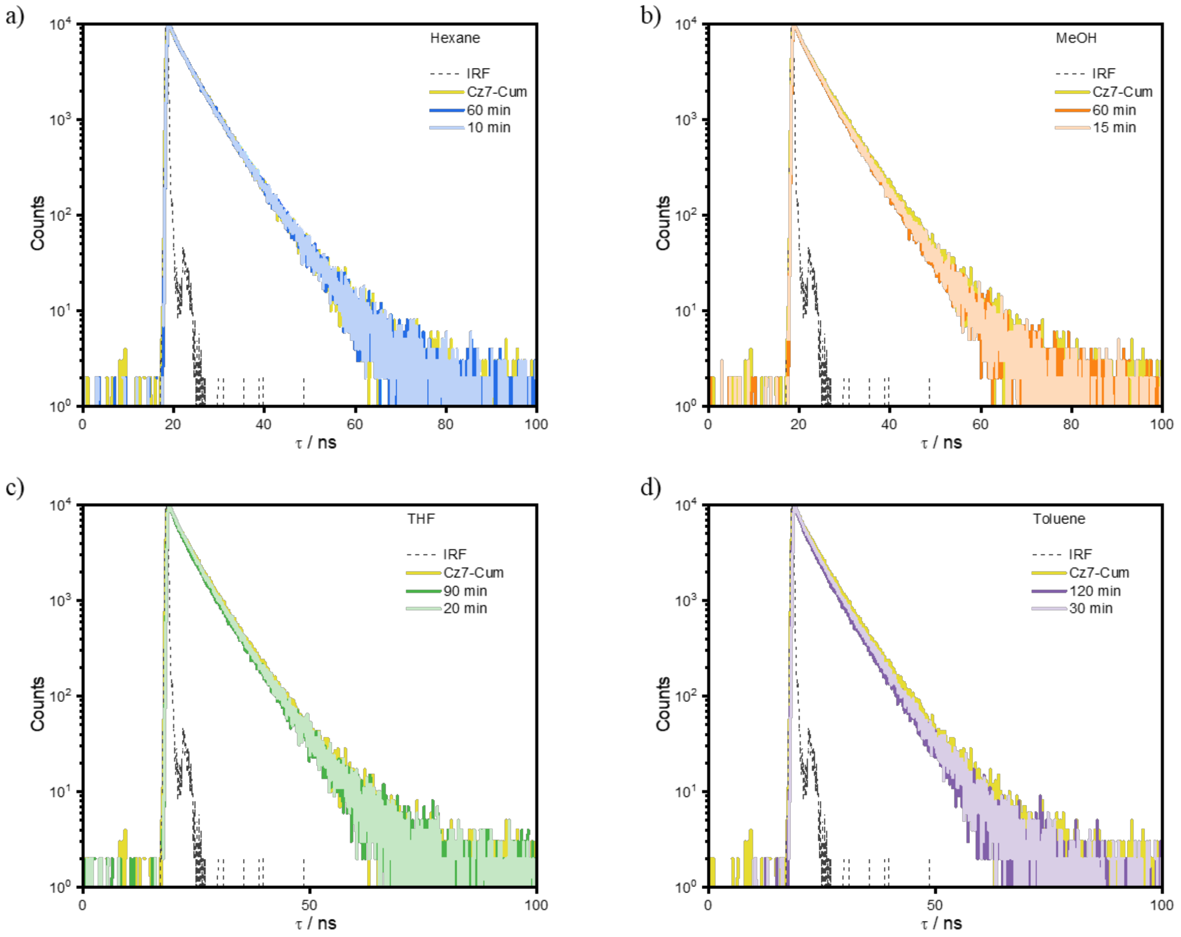

Figure S48. Time-resolved PL decays of the 0.2 wt% **Cz7-Cum** (λ_{PL} = 490 nm) in HDDA 70% - HPPA 30% 3D printed materials upon exposure to a) hexane, b) MeOH, c) THF and d) toluene, recorded by TCSPC, λ_{exc} = 375 nm.

Ethyl 7-(3,6-di-tert-butyl-9H-carbazol-9-yl)-2-oxo-2H-chromene-3-carboxylate ((*t*-Bu)Cz7-Cum)

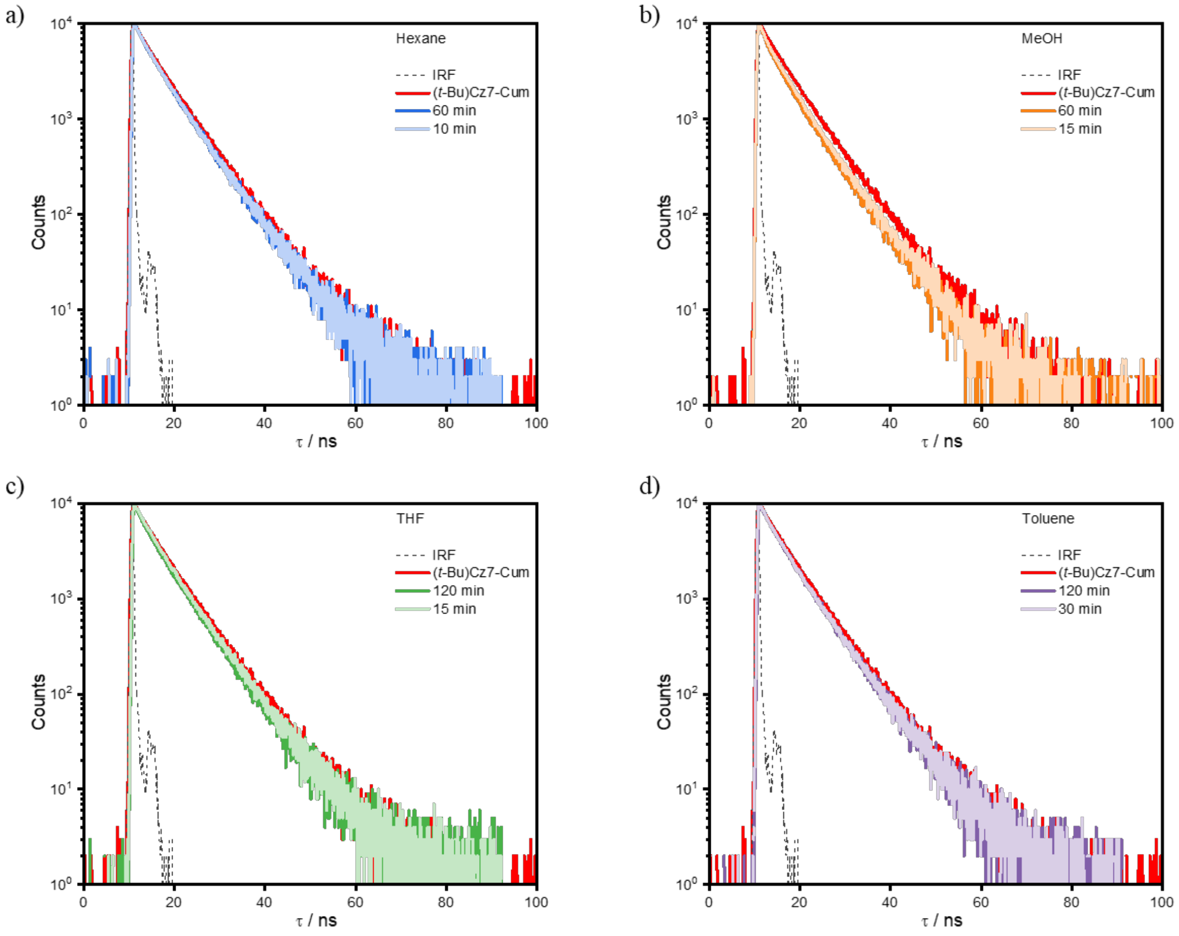

Figure S49. Time-resolved PL decays of the 0.2 wt% **(*t*-Bu)Cz7-Cum** (λ_{PL} = 518 nm) in HDDA 70% - HPPA 30% 3D printed materials upon exposure to a) hexane, b) MeOH, c) THF, and d) toluene, recorded by TCSPC, λ_{exc} = 375 nm.

Ethyl 7-(12H-benzo[4,5]thieno[2,3-a]71arbazole-12-yl)-2-oxo-2H-chromene-3-carboxylate (BTCz7-Cum)

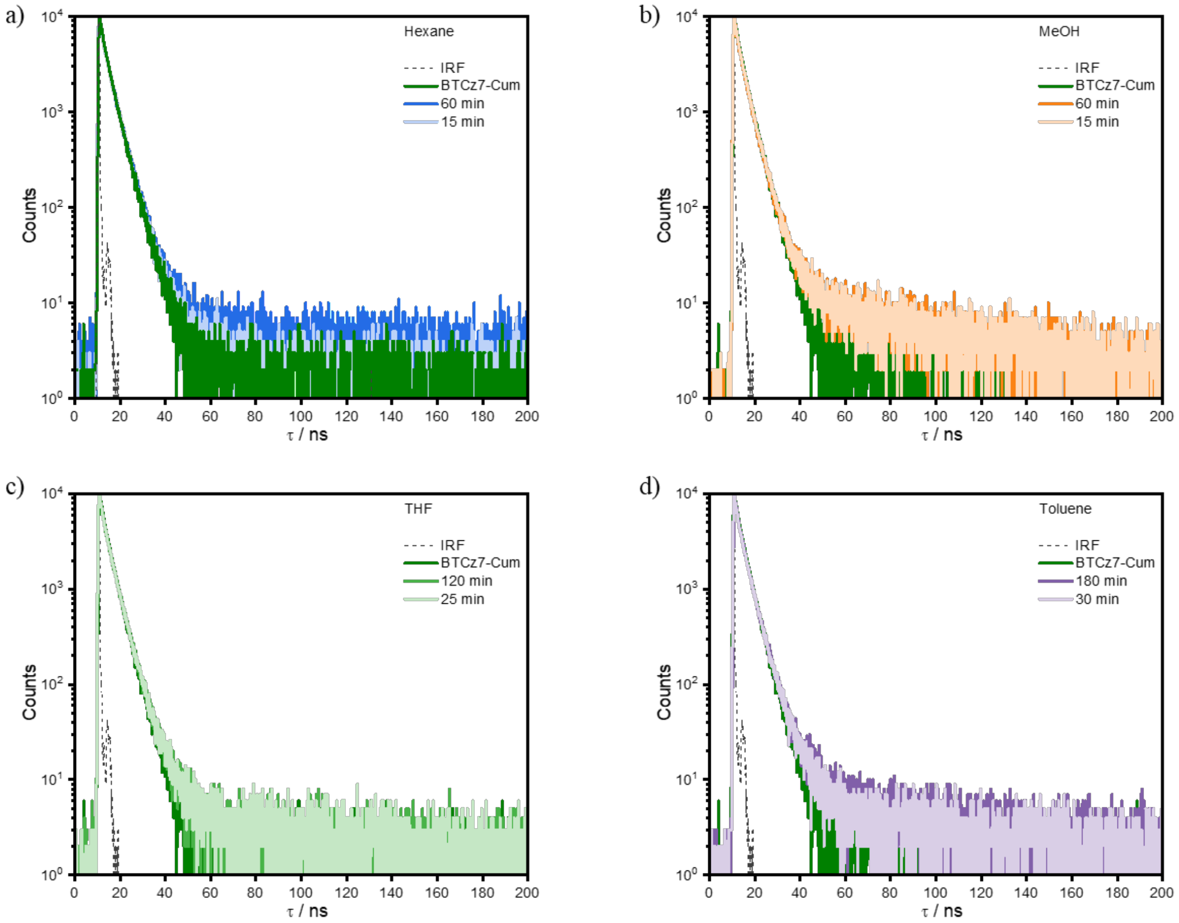

Figure S50. Time-resolved PL decays of the 0.2 wt% **BTCz7-Cum** (λ_{PL} = 510 nm) in HDDA 70% - HPPA 30% 3D printed materials upon exposure to a) hexane, b) MeOH, c) THF and d) toluene, recorded by TCSPC, λ_{exc} = 375 nm.

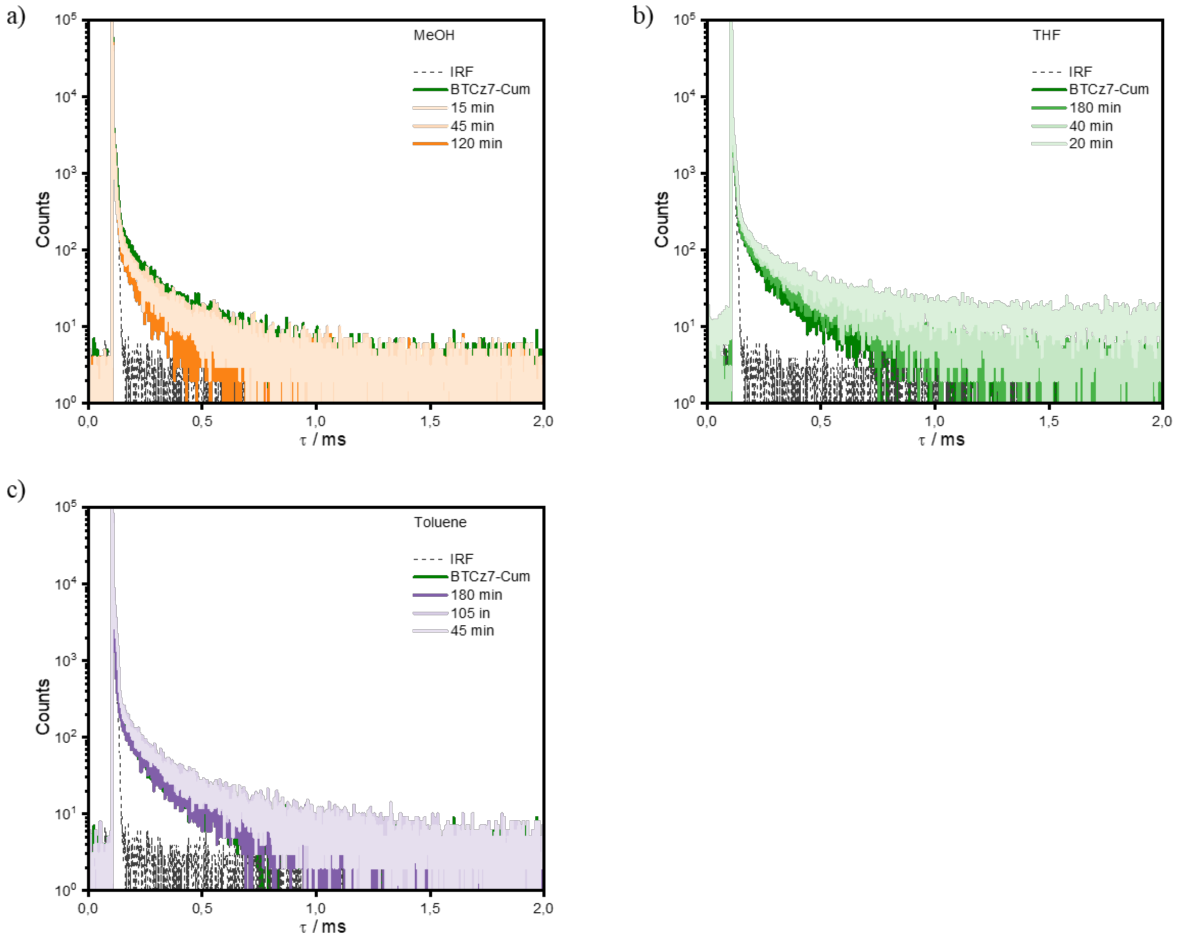

Figure S51. Time-resolved decays of the delayed emission of **BTCz7-Cum** (λ_{exc} = 390 nm, λ_{PL} = 510 nm) in 0.2 wt% 3D-printed material in HDDA 70% - HPPA 30% upon exposure to a) MeOH, b) THF and c) toluene, collected by multi-channel scaling (MCS).

Diethyl 7,7'-(indolo[3,2-b]carbazole-5,11-diyl)bis(2-oxo-2H-chromene-3-carboxylate) (ICz7-Cum) spectra

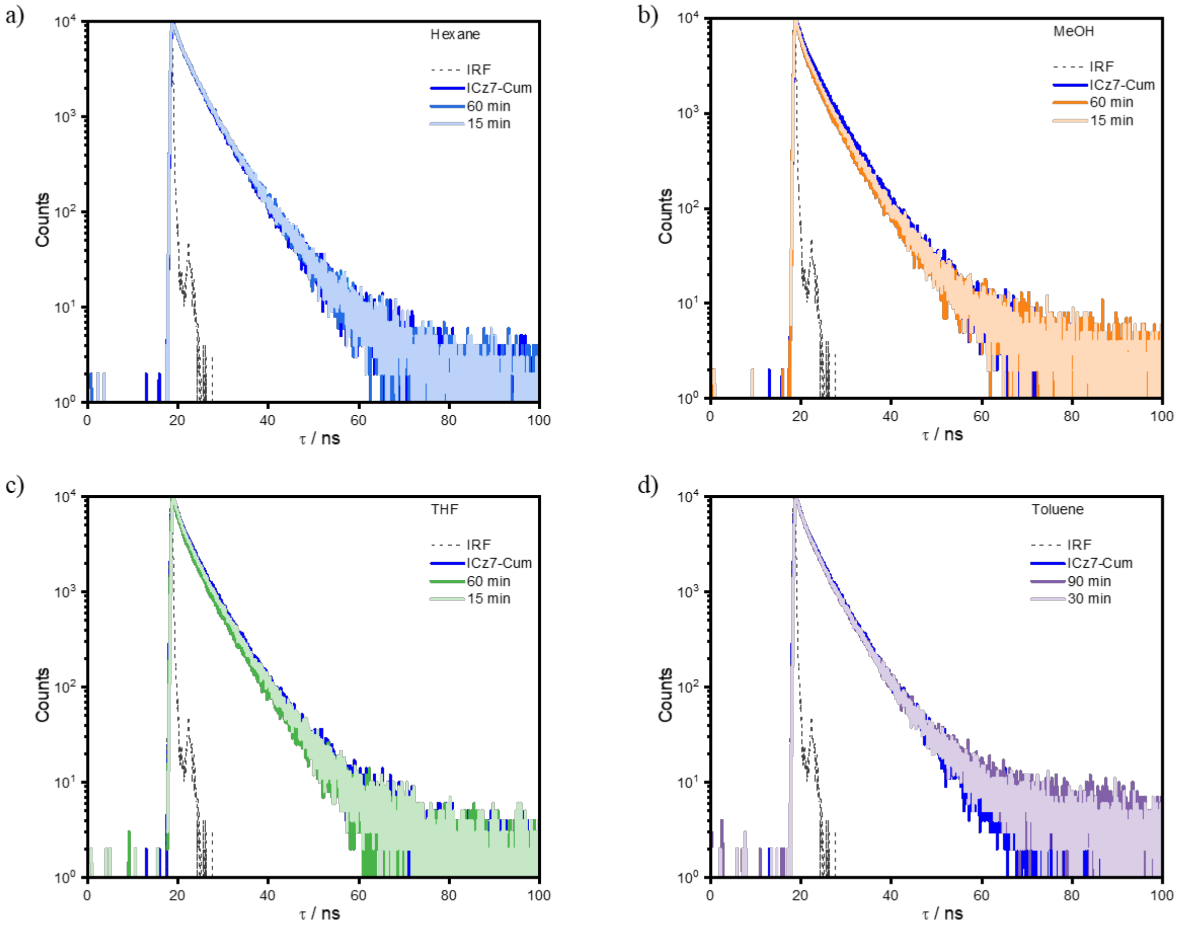

Figure S52. Time-resolved PL decays of the 0.1 wt% **ICz7-Cum** (λ_{PL} = 562 nm) in HDDA 70% - HPPA30% 3D printed materials upon exposure to *a)* hexane, *b)* MeOH, *c)* THF and *d)* toluene, recorded by TCSPC, λ_{exc} = 375 nm.

Spectroscopic UV-Vis absorption and PLQY analysis

Ethyl 7-(9H-carbazol-9-yl)-2-oxo-2H-chromene-3-carboxylate (Cz7-Cum)

Table S16. Photophysical properties of 3D printed 0.2 wt% **Cz7-Cum** in HDDA 70% - HPPA 30% when exposed to acetone vapours.

	No solvent	After acetone	Δ / %
**Absorbance / %**	18.12	31.8	+74.8
**EQE / %**	6.2	10.9	+77.1
**IQE / %**	33.8	34.3	+1.3

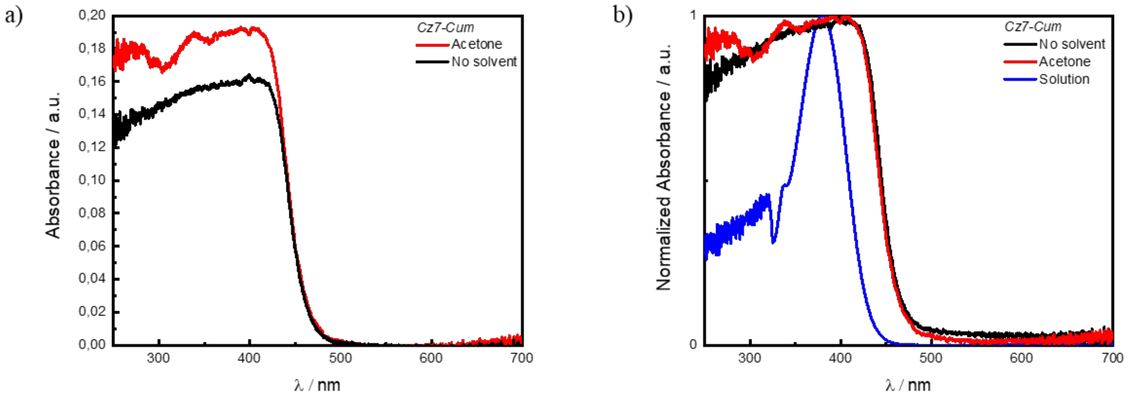

Figure S53. UV-Vis absorption spectra of 3D printed 0.2 wt% **Cz7-Cum** embedded in HDDA 70% - HPPA 30%. a) Comparison before and after exposure to acetone vapor. b) Normalized absorption spectra of the same sample compared with **Cz7-Cum** in acetone solution (≈ 10^{-5} M).

Table S17. Photophysical properties of 3D printed 0.2 wt% **Cz7-Cum** in HDDA 70% - HPPA 30% when exposed to DCE vapours.

	No solvent	After DCE	Δ / %
**Absorbance / %**	26.5	28.2	+6.4
**EQE / %**	8.6	9.7	+12.8
**IQE / %**	32.5	34.6	+6.5

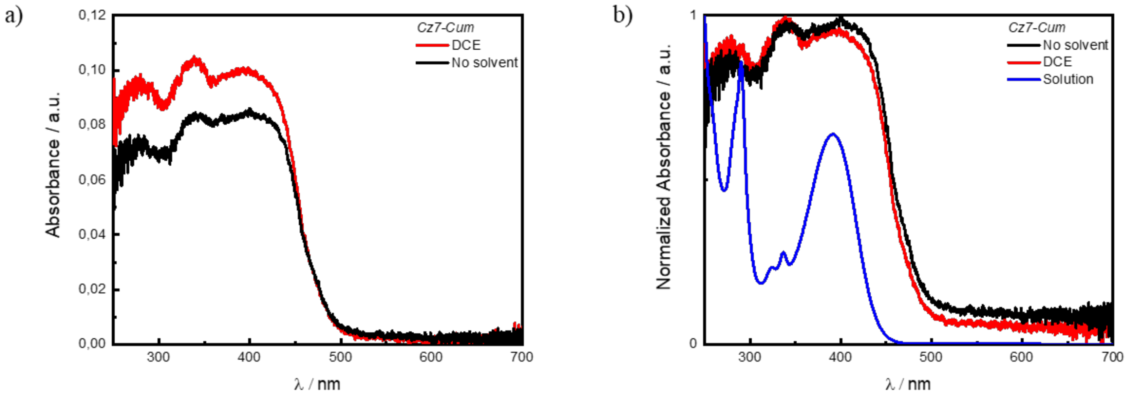

Figure S54. UV-Vis absorption spectra of 3D printed 0.2 wt% **Cz7-Cum** embedded in 70% HDDA – 30% HPPA. a) Comparison before and after exposure to DCE vapor. b) Normalized absorption spectra of the same sample compared with **Cz7-Cum** in DCE solution (≈ 10^{-5} M).

Ethyl 7-(3,6-di-tert-butyl-9H-carbazol-9-yl)-2-oxo-2H-chromene-3-carboxylate ((*t*-Bu)Cz7-Cum)

Table S18. Photophysical properties of 3D printed 0.2 wt% **(*t*-Bu)Cz7-Cum** in HDDA 70% - HPPA 30% when exposed to acetone vapours.

	No solvent	After acetone	Δ / %
**Absorbance / %**	25.0	36.2	+44.8
**EQE / %**	5.6	6.6	+17.9
**IQE / %**	22.4	18.1	-19.2

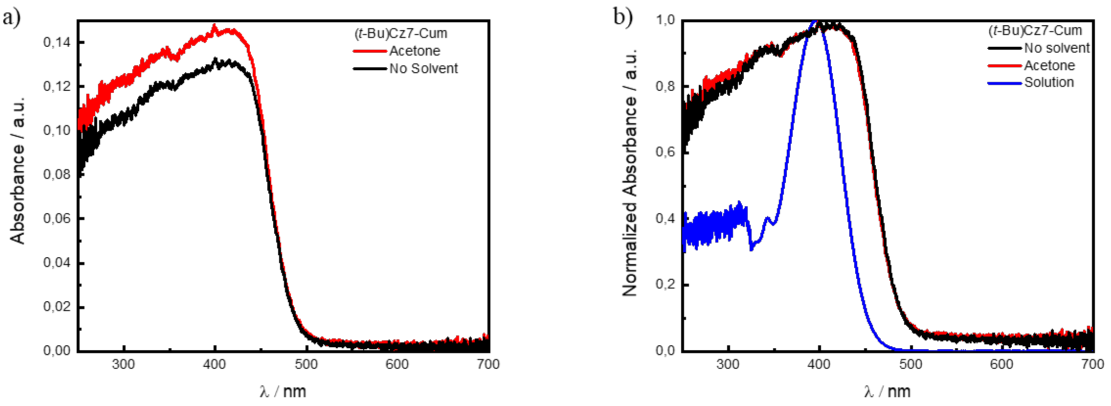

Figure S55. UV-Vis absorption spectra of 3D printed 0.2 wt% **(*t*-Bu)Cz7-Cum** embedded in HDDA 70% - HPPA 30%. a) Comparison before and after exposure to acetone vapor. b) Normalized absorption spectra of the same sample compared with **(*t*-Bu)Cz7-Cum** in acetone solution (≈ 10^{-5} M).

Table S19. Photophysical properties of 3D printed 0.2 wt% **(*t*-Bu)Cz7-Cum** in HDDA 70% - HPPA 30% when exposed to DCE vapours.

	**No solvent**	**After DCE**	**Δ / %**
**Absorbance / %**	25.4	33.4	+31.5
**EQE / %**	5.5	5.5	+/- 0
**IQE / %**	21.6	16.5	-23.6

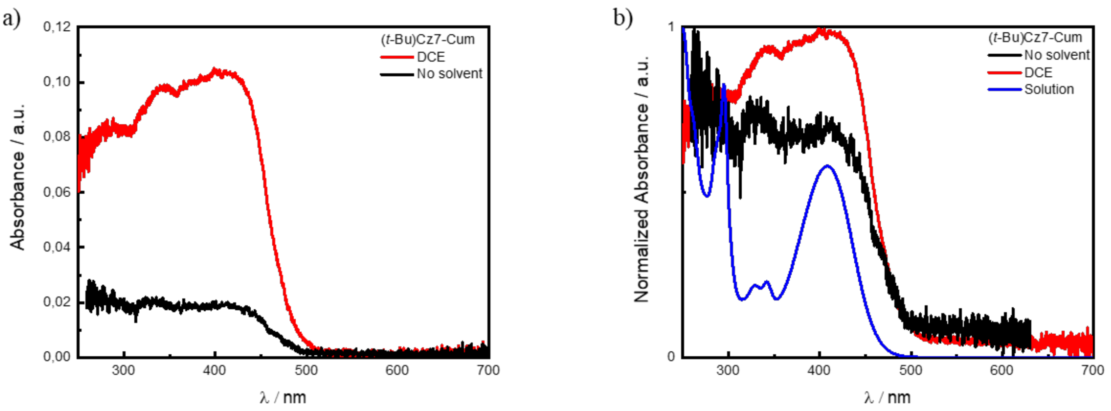

Figure S56. UV-Vis absorption spectra of 3D printed 0.2 wt% **(*t*-Bu)Cz7-Cum** embedded in HDDA 70% - HPPA 30%. a) Comparison before and after exposure to DCE vapor. b) Normalized absorption spectra of the same sample compared with **(*t*-Bu)Cz7-Cum** in DCE solution (≈10^{-5} M).

Ethyl 7-(12H-benzo[4,5]thieno[2,3-a]carbazol-12-yl)-2-oxo-2H-chromene-3-carboxylate (BTCz7-Cum)

Table S20. Photophysical properties of 3D printed 0.2 wt% **BTCz7-Cum** in HDDA 70% - HPPA 30% when exposed to hexane vapours.

	No solvent	After Hexane	Δ / %
**Absorbance / %**	21.2	14.8	-30.3
**EQE / %**	1.7	1.2	-26.2
**IQE / %**	8.0	8.4	+6.0

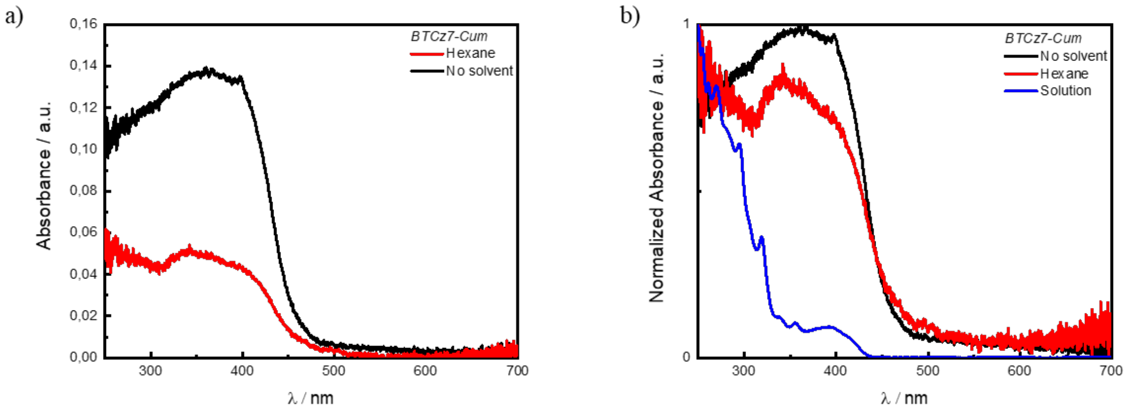

Figure S57. UV-Vis absorption spectra of 3D printed 0.2 wt% **BTCz7-Cum** embedded in HDDA 70% - HPPA 30%. a) Comparison before and after exposure to hexane vapor. b) Normalized absorption spectra of the same sample compared with **BTCz7-Cum** in hexane solution (≈10^{-5} M).

Table S21. Photophysical properties of 3D printed 0.2 wt% **BTCz7-Cum** in HDDA 70% - HPPA30% when exposed to toluene vapours.

	No solvent	After Toluene	Δ / %
**Absorbance / %**	20.5	10.9	-46.5
**EQE / %**	2.3	1.9	-19.7
**IQE / %**	11.3	17.0	+50.2

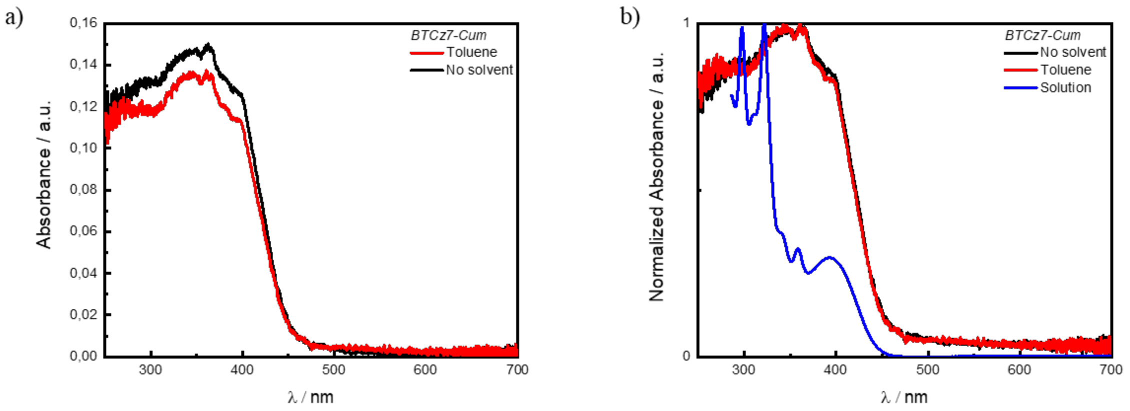

Figure S58. UV-Vis absorption spectra of 3D printed 0.2 wt% **BTCz7-Cum** embedded in HDDA 70% - HPPA 30%. a) Comparison before and after exposure to toluene vapor. b) Normalized absorption spectra of the same sample compared with **BTCz7-Cum** in toluene solution (≈10^{-5} M).

Table S22. Photophysical properties of 3D printed 0.2 wt% **BTCz7-Cum** in HDDA 70% - HPPA 30% when exposed to benzene vapours.

	No solvent	After Benzene	Δ / %
**Absorbance / %**	15.7	1.3	-91.9
**EQE / %**	1.8	0.7	-60.4
**IQE / %**	11.4	55.7	+388.3

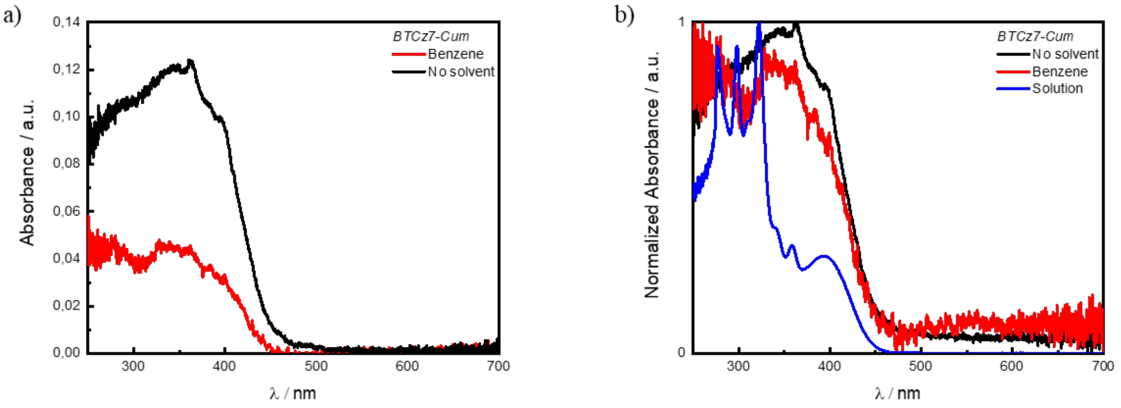

Figure S59. UV-Vis absorption spectra of 3D printed 0.2 wt% **BTCz7-Cum** embedded in HDDA 70% - HPPA 30%. a) Comparison before and after exposure to benzene vapor. b) Normalized absorption spectra of the same sample compared with **BTCz7-Cum** in benzene solution (≈10^{-5} M).

Table S23. Photophysical properties of 3D printed 0.2 wt% **BTCz7-Cum** in HDDA 70% - HPPA 30% when exposed to diethyl ether vapours.

	No solvent	After Diethyl Ether	Δ / %
**Absorbance / %**	13.9	22.9	+64.7
**EQE / %**	1.3	1.6	+2.3
**IQE / %**	9.1	7.1	-28.2

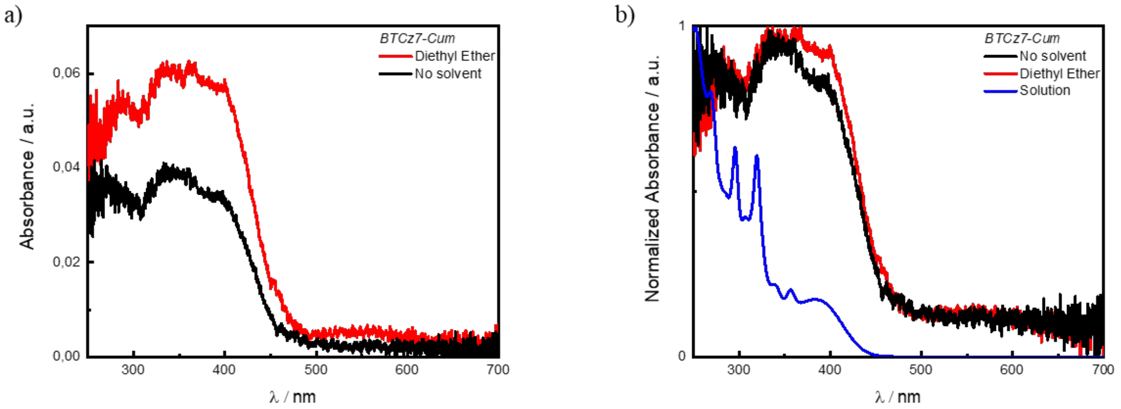

Figure S60. UV-Vis absorption spectra of 3D printed 0.2 wt% **BTCz7-Cum** embedded in HDDA 70% - HPPA 30%. *a)* Comparison before and after exposure to diethyl ether vapor. *b)* Normalized absorption spectra of the same sample compared with **BTCz7-Cum** in diethyl ether solution (≈10^{-5} M).

Diethyl 7,7'-(indolo[3,2-b]carbazole-5,11-diyl)bis(2-oxo-2H-chromene-3-carboxylate) (ICz7-Cum) spectra

Table S24. Photophysical properties of 3D printed 0.1 wt% **ICz7-Cum** in HDDA 70% - HPPA 30% when exposed to benzene vapours.

	No solvent	After Benzene	Δ / %
**Absorbance / %**	23.2	29.5	+27.5
**EQE / %**	2.4	2.6	+6.5
**IQE / %**	10.4	8.7	-16.5

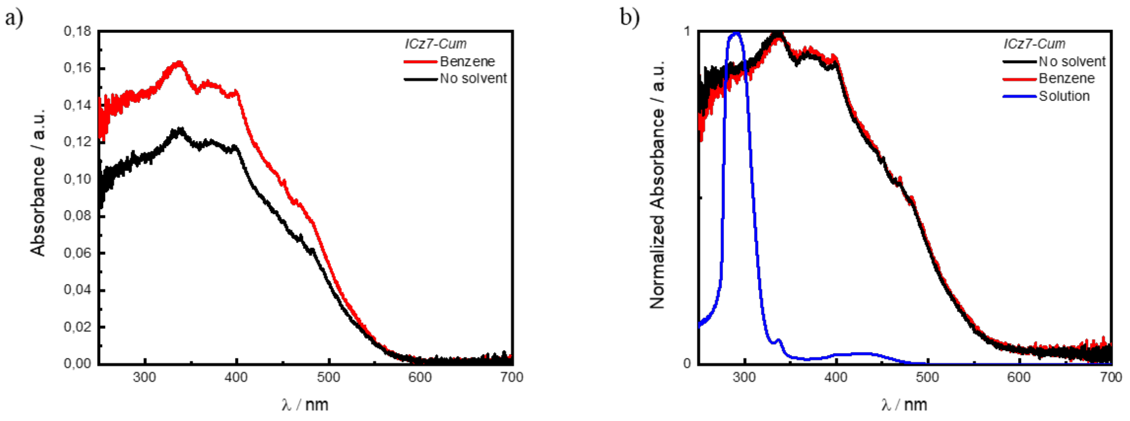

Figure S61. UV-Vis absorption spectra of 3D printed 0.1 wt% **ICz7-Cum** embedded in HDDA 70% - HPPA 30%. a) Comparison before and after exposure to benzene vapor. b) Normalized absorption spectra of the same sample compared with **ICz7-Cum** in benzene solution (≈10^{-5} M).

Table S25. Photophysical properties of 3D printed 0.1 wt% **ICz7-Cum** in HDDA 70% - HPPA 30% when exposed to ethyl acetate vapours.

	**No solvent**	**After Ethyl Acetate**	**Δ / %**
**Absorbance / %**	11.2	17.3	+54.5
**EQE / %**	2.1	2.7	+27.6
**IQE / %**	18.8	15.6	-17.3

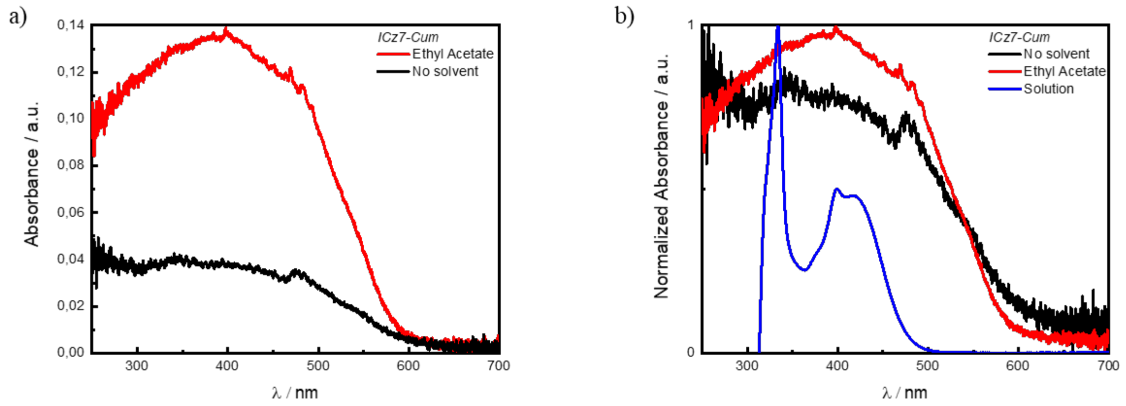

Figure S62. UV-Vis absorption spectra of 3D printed 0.1 wt% **ICz7-Cum** embedded in HDDA 70% - HPPA 30%. *a)* Comparison before and after exposure to ethyl acetate vapor. *b)* Normalized absorption spectra of the same sample compared with **ICz7-Cum** in ethyl acetate solution (≈10^{-5} M).

Table S26. Photophysical properties of 3D printed 0.1 wt% **ICz7-Cum** in HDDA 70% - HPPA 30% when exposed to MeOH vapours.

	No solvent	After MeOH	Δ / %
**Absorbance / %**	24.8	12.6	-49.3
**EQE / %**	2.3	1.4	-39.2
**IQE / %**	9.2	11.0	+20.0

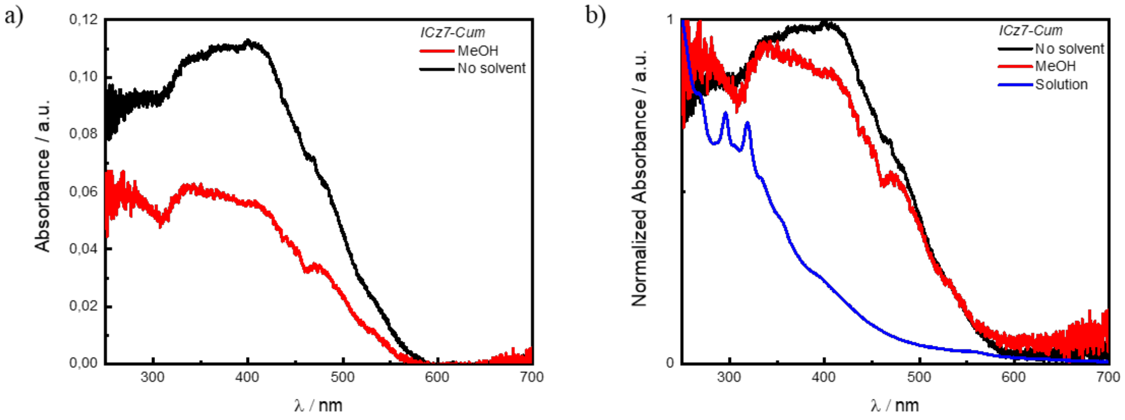

Figure S63. UV-Vis absorption spectra of 3D printed 0.1 wt% **ICz7-Cum** embedded in HDDA 70% - HPPA 30%. *a)* Comparison before and after exposure to MeOH vapor. *b)* Normalized absorption spectra of the same sample compared with **ICz7-Cum** in MeOH solution (≈10^{-5} M).

Repeatability

Ethyl 7-(9H-carbazol-9-yl)-2-oxo-2H-chromene-3-carboxylate (Cz7-Cum) in DCE

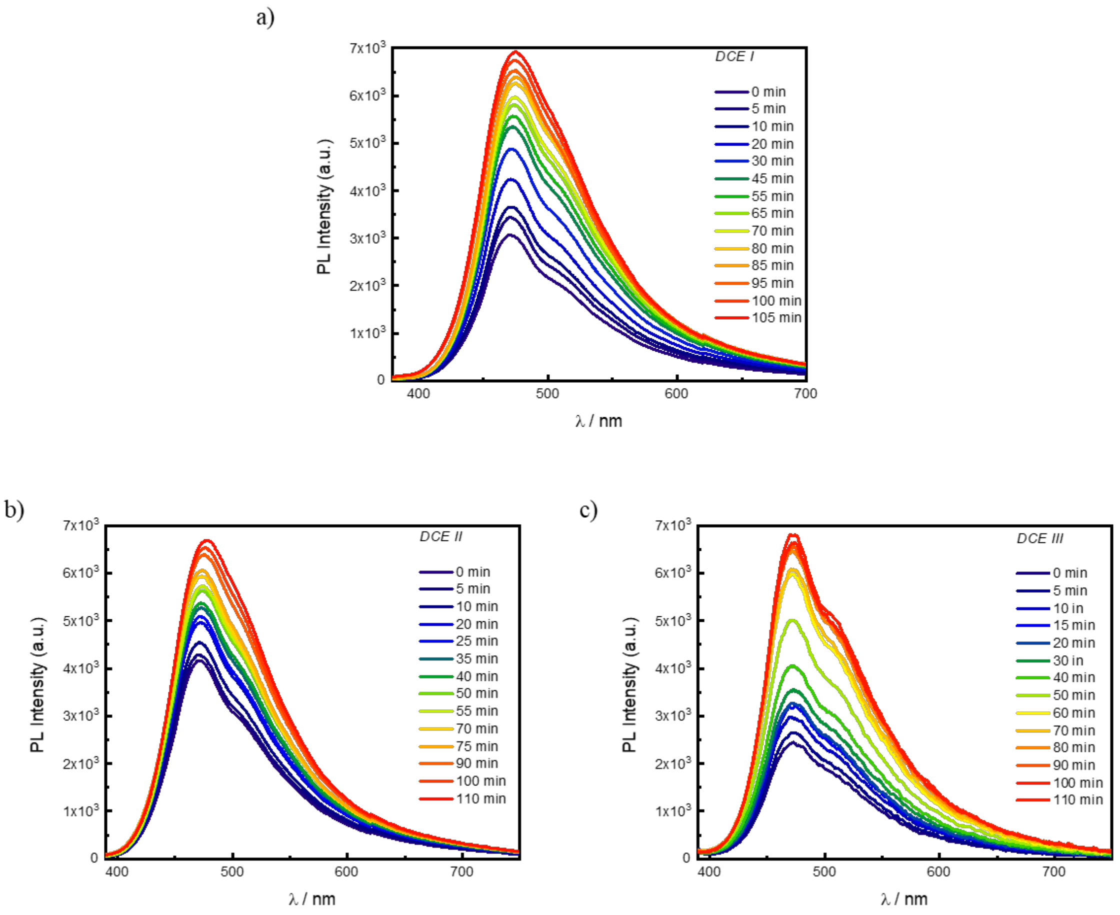

Figure S64. Emission spectra of three different, a), b), c) film composed of HDDA 70% - HPPA 30% containing 0.2 wt% **Cz7-Cum** (λ_{exc} = 360 nm), recorded over time during exposure to DCE, demonstrating consistent fluorescence response.

Ethyl 7-(9H-carbazol-9-yl)-2-oxo-2H-chromene-3-carboxylate (Cz7-Cum) in acetone

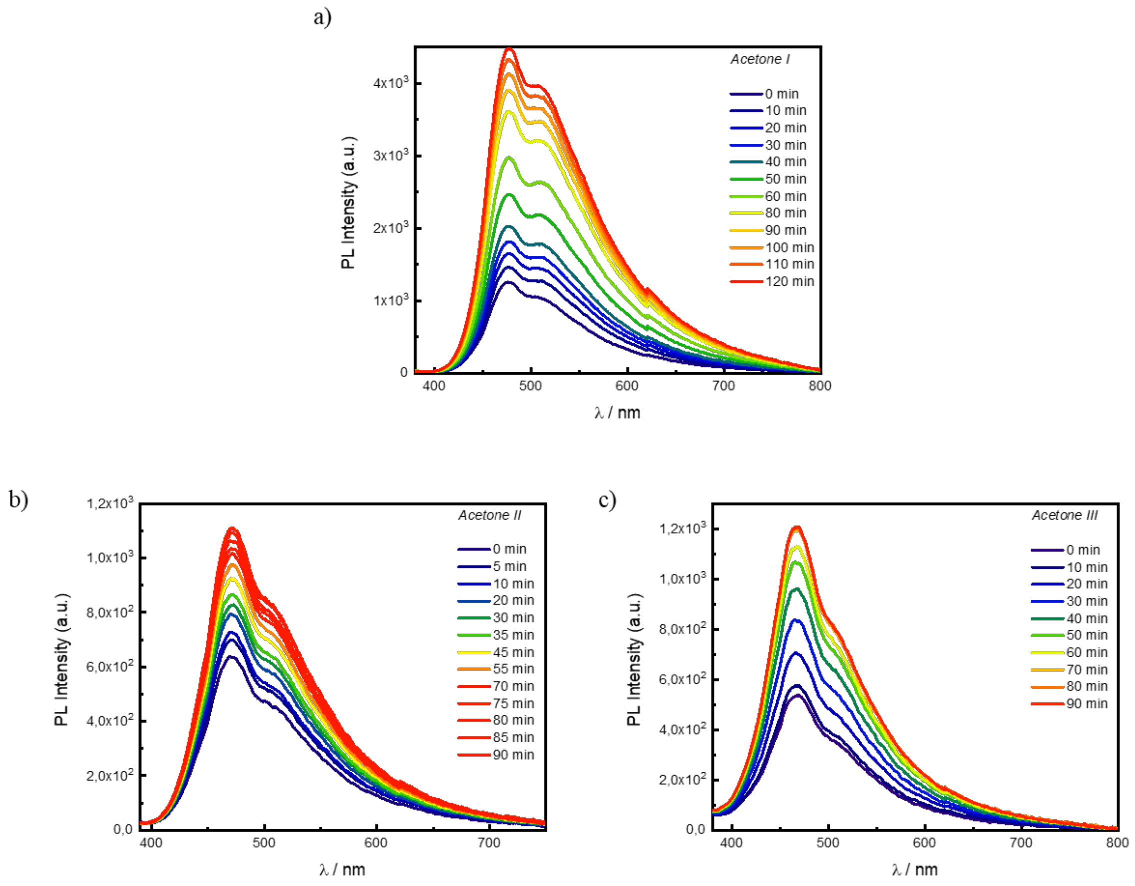

Figure S65. Emission spectra of three different, a), b), c) film composed of HDDA 70% - HPPA 30% containing 0.2 wt% **Cz7-Cum** (λ_{exc} = 360 nm), recorded over time during exposure to acetone, demonstrating consistent fluorescence response.

Ethyl 7-(3,6-di-tert-butyl-9H-carbazol-9-yl)-2-oxo-2H-chromene-3-carboxylate ((*t*-Bu)Cz7-Cum) in DCE

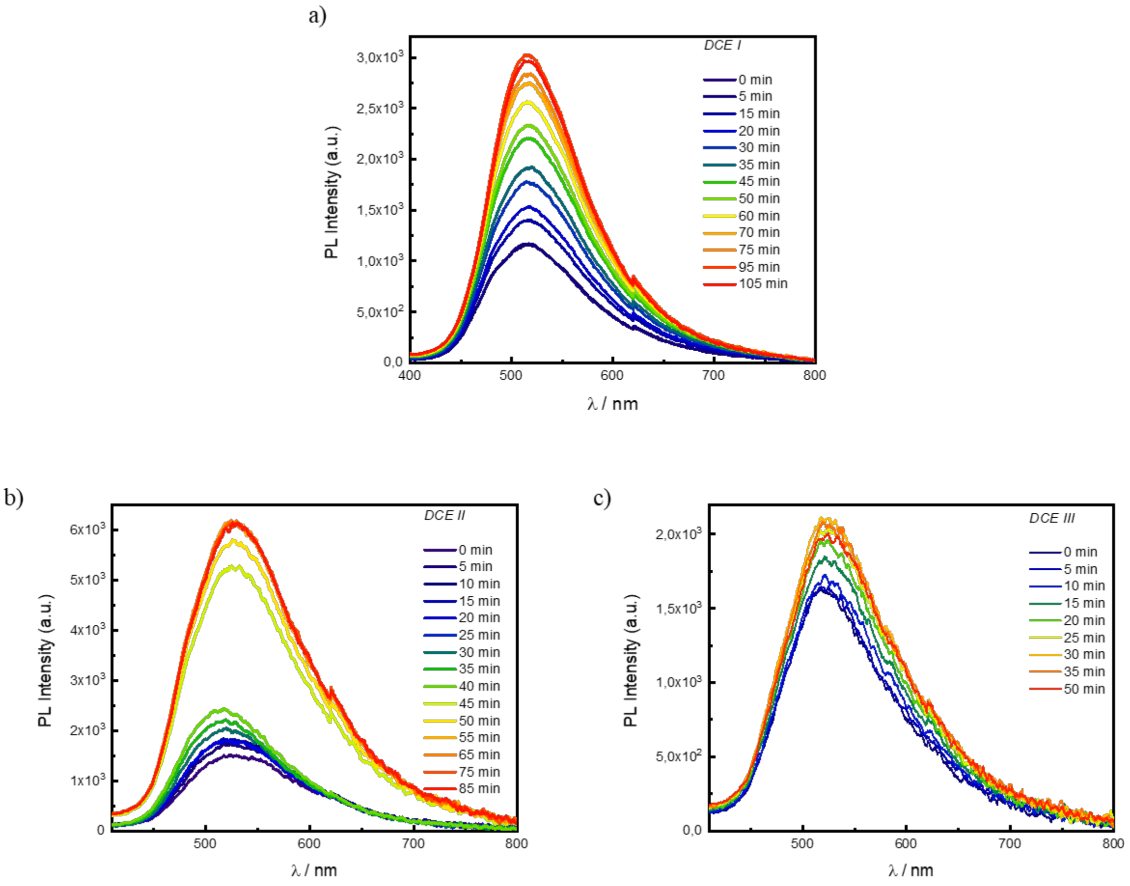

Figure S66. Emission spectra of three different, a), b), c) film composed of HDDA 70% - HPPA 30% containing 0.2 wt% **(*t*-Bu)Cz7-Cum** (λ_{exc} = 340 nm), recorded over time during exposure to DCE, demonstrating consistent fluorescence response.

It should be noted that the absolute values of the PL emission are not fully reproducible across measurements. This variation is most likely because the analysed samples were collected from 3D-printed batches prepared at different times. Nevertheless, the observed trend of increasing PL emission is consistent and in line with the expected behaviour.

Ethyl 7-(3,6-di-tert-butyl-9H-carbazol-9-yl)-2-oxo-2H-chromene-3-carboxylate ((*t*-Bu)Cz7-Cum) in acetone

a)

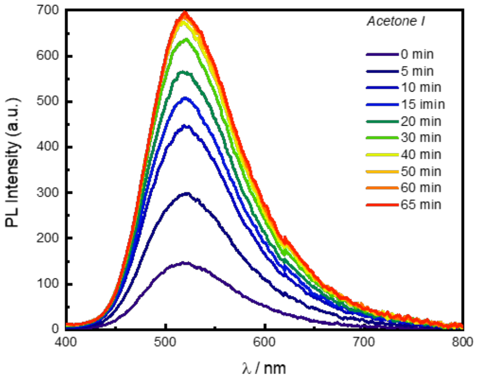

b)

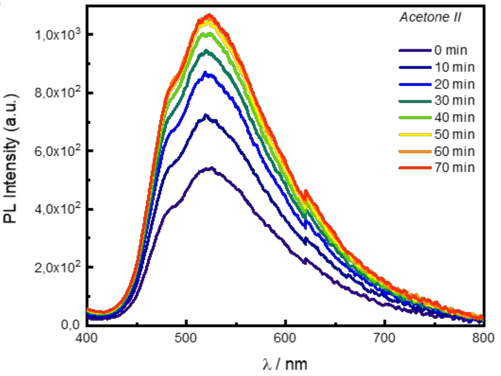

c)

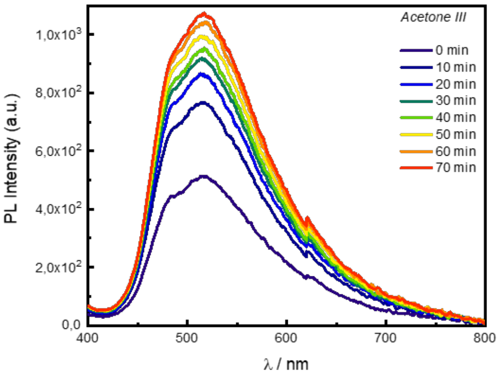

Figure S67. Emission spectra of three different, a), b), c) film composed of HDDA 70% - HPPA 30% containing 0.2 wt% **(*t*-Bu)Cz7-Cum** (λ_{exc} = 340 nm), recorded over time during exposure to acetone, demonstrating consistent fluorescence response.

Ethyl 7-(12H-benzo[4,5]thieno[2,3-a]carbazol-12-yl)-2-oxo-2H-chromene-3-carboxylate (BTCz7-Cum) in hexane

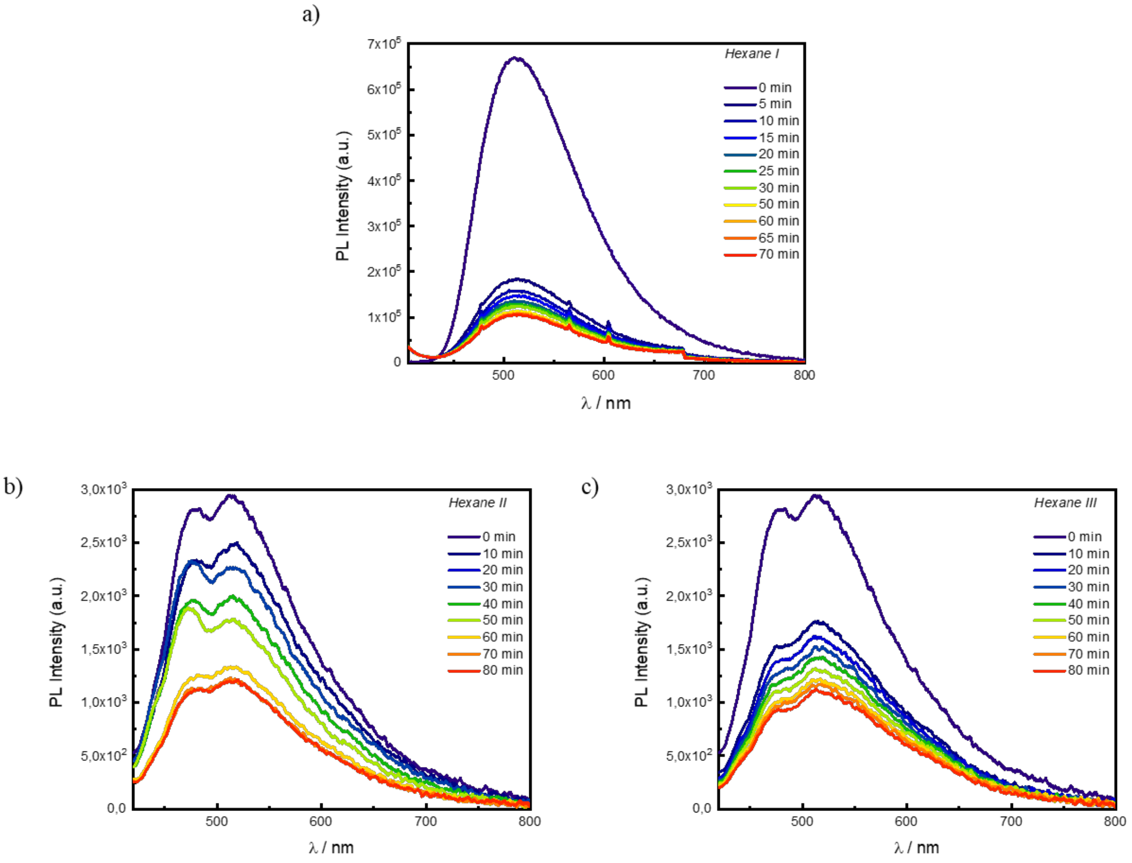

Figure S68. Emission spectra of three different, a), b), c) film composed of HDDA 70% - HPPA 30% containing 0.2 wt% **BTCz7-Cum** (λ_{exc} = 390 nm), recorded over time during exposure to hexane, demonstrating consistent fluorescence response.

Ethyl 7-(12H-benzo[4,5]thieno[2,3-a]carbazol-12-yl)-2-oxo-2H-chromene-3-carboxylate (BTCz7-Cum) in toluene

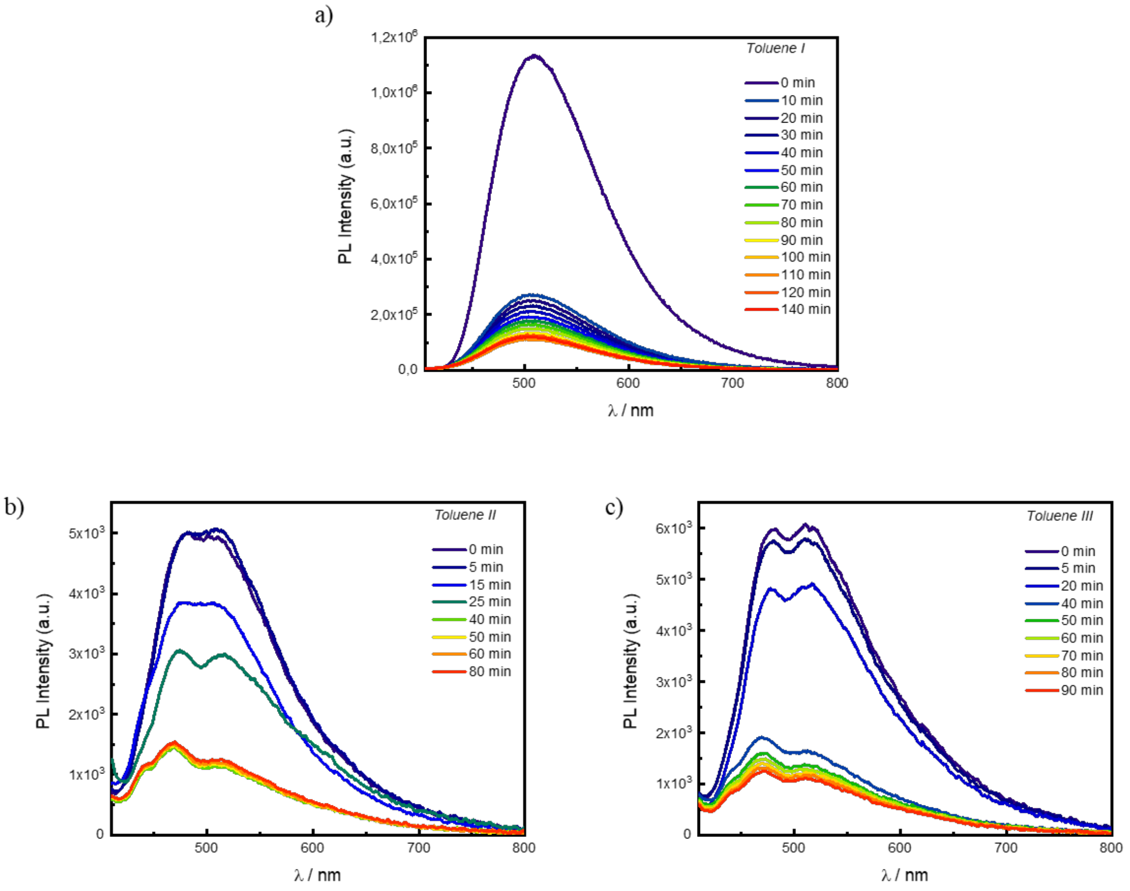

Figure S69. Emission spectra of three different, a), b), c) film composed of HDDA 70% - HPPA 30% containing 0.2 wt% **BTCz7-Cum** (λ_{exc} = 390 nm), recorded over time during exposure to toluene, demonstrating consistent fluorescence response.

Ethyl 7-(12H-benzo[4,5]thieno[2,3-a]carbazol-12-yl)-2-oxo-2H-chromene-3-carboxylate (BTCz7-Cum) in benzene

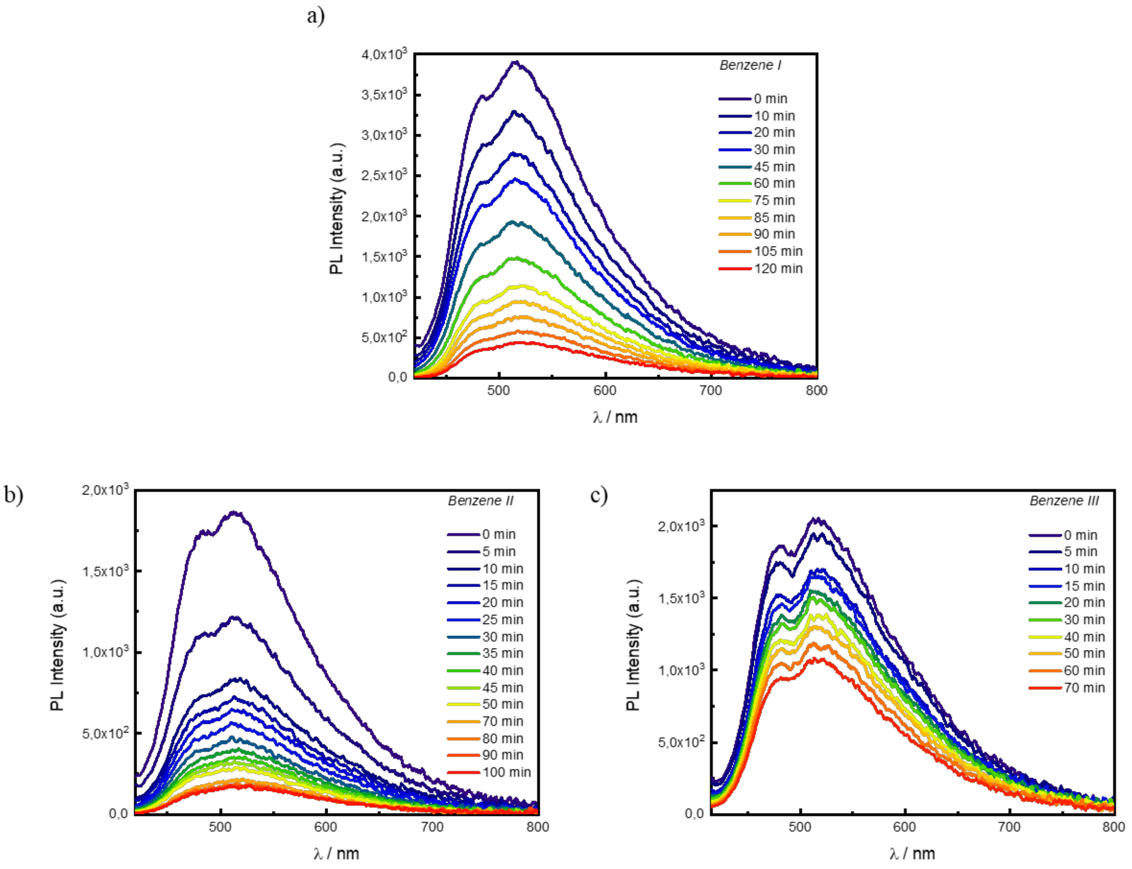

Figure S70. Emission spectra of three different, a), b), c) film composed of HDDA 70% - HPPA 30% containing 0.2 wt% **BTCz7-Cum** (λ_{exc} = 390 nm), recorded over time during exposure to benzene, demonstrating consistent fluorescence response.

Ethyl 7-(12H-benzo[4,5]thieno[2,3-a]carbazol-12-yl)-2-oxo-2H-chromene-3-carboxylate (BTCz7-Cum) in diethyl ether

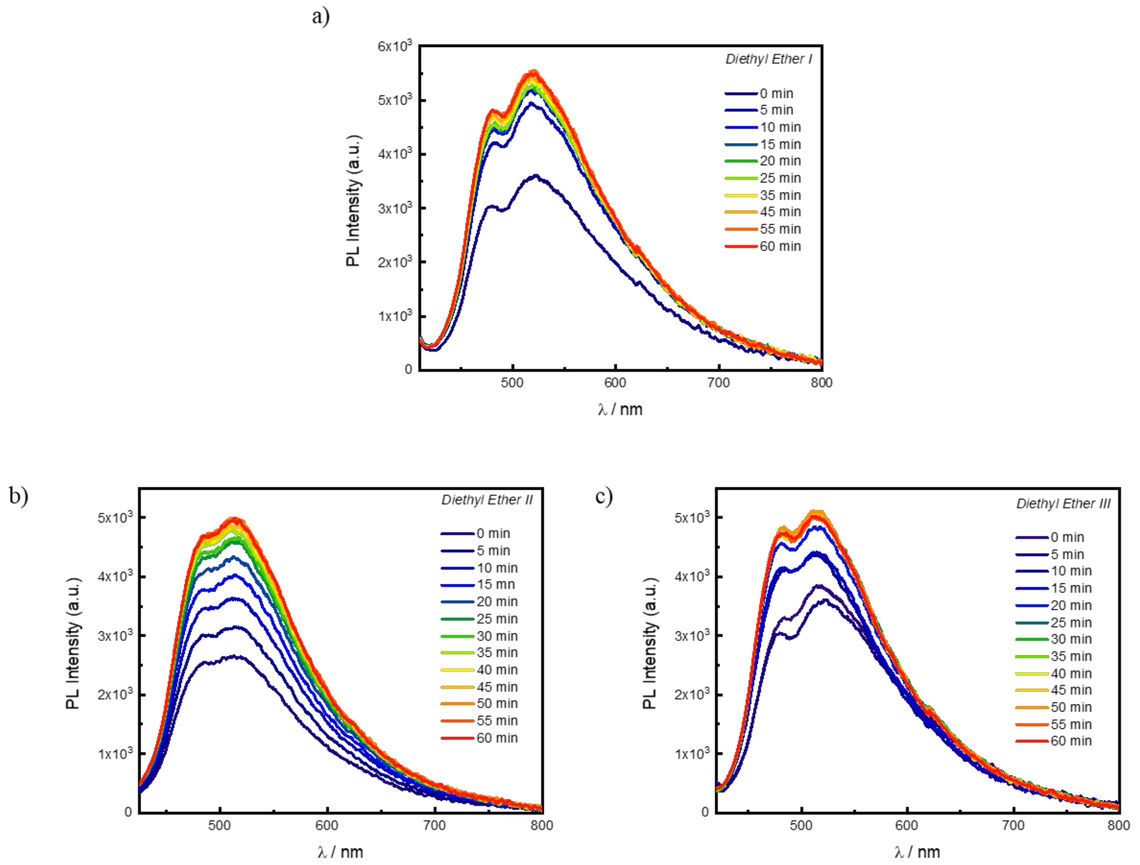

Figure S71. Emission spectra of three different, a), b), c*)* film composed of HDDA 70% - HPPA 30% containing 0.2 wt% **Cum-7BTCz** (λ_{exc} = 390 nm), recorded over time during exposure to diethyl ether, demonstrating consistent fluorescence response.

Diethyl 7,7'-(indolo[3,2-b]carbazole-5,11-diyl)bis(2-oxo-2H-chromene-3-carboxylate) (ICz7-Cum) spectra in benzene

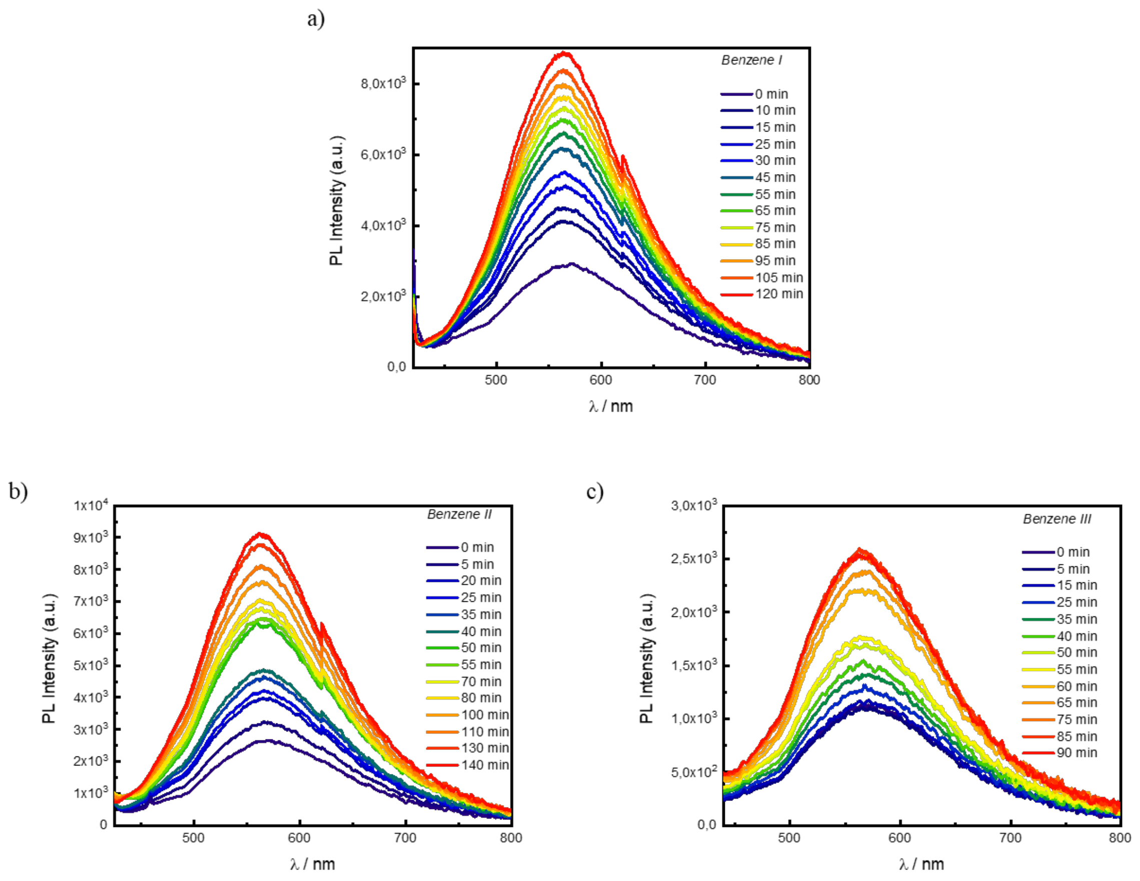

Figure S72. Emission spectra of three different, a), b), c) film composed of HDDA 70% - HPPA 30% containing 0.1 wt% **ICz7-Cum** (λ_{exc} = 400 nm), recorded over time during exposure to benzene, demonstrating consistent fluorescence response.

Diethyl 7,7'-(indolo[3,2-b]carbazole-5,11-diyl)bis(2-oxo-2H-chromene-3-carboxylate) (ICz7-Cum) spectra in ethyl acetate

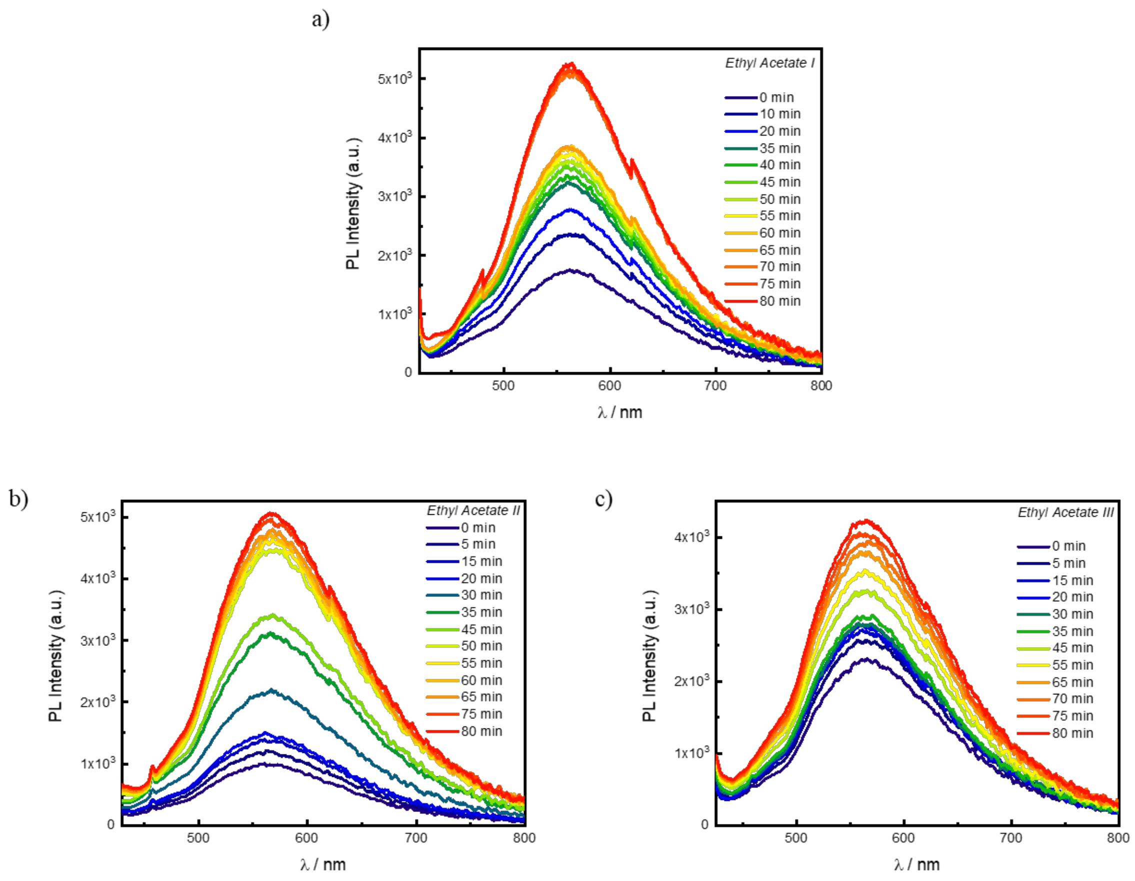

Figure S73. Emission spectra of three different, a), b), c) film composed of HDDA 70% - HPPA 30% containing 0.1 wt% **ICz7-Cum** (λ_{exc} = 400 nm), recorded over time during exposure to ethyl acetate, demonstrating consistent fluorescence response.

Diethyl 7,7'-(indolo[3,2-b]carbazole-5,11-diyl)bis(2-oxo-2H-chromene-3-carboxylate) (ICz7-Cum) spectra in MeOH

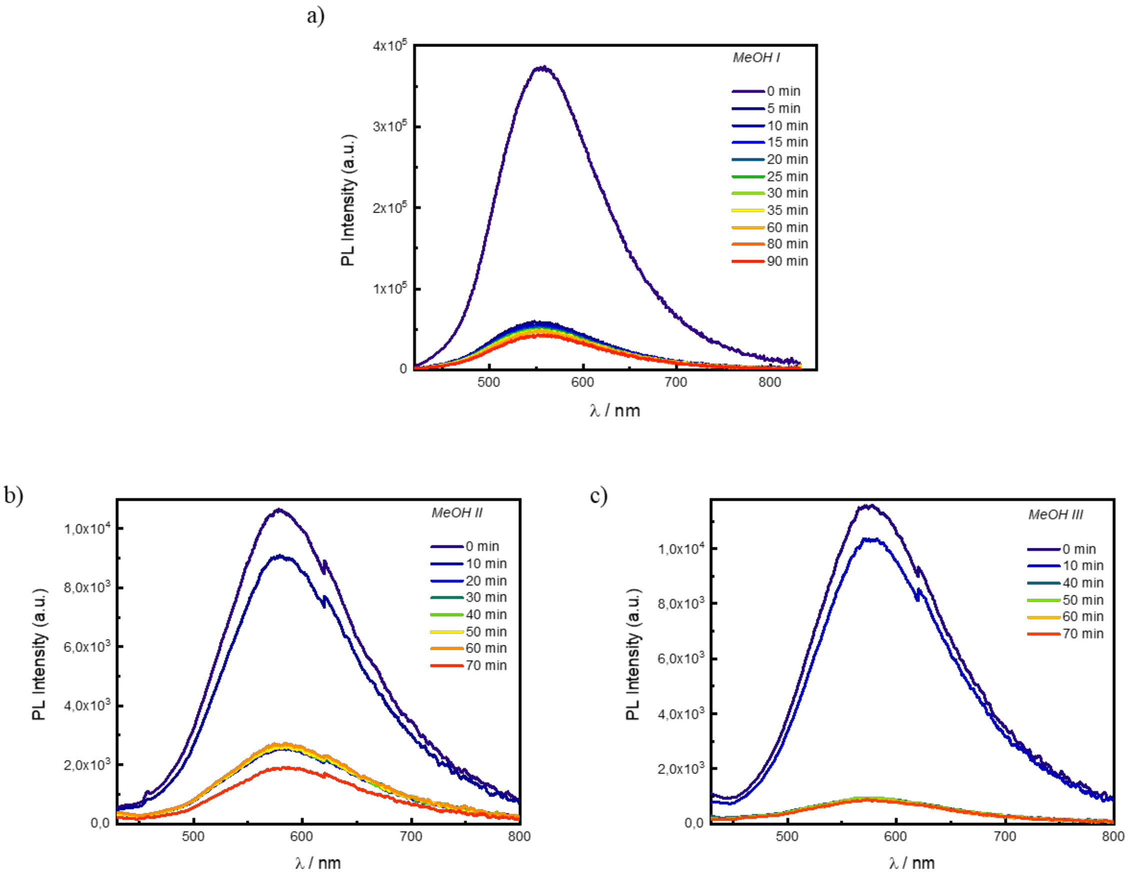

Figure S74. Emission spectra of three different, a), b), c) film composed of HDDA 70% - HPPA 30% containing 0.1 wt% **ICz7-Cum** (λ_{exc} = 400 nm), recorded over time during exposure to methanol, demonstrating consistent fluorescence response.

References

[1] G. M. Sheldrick, *Acta Crystallogr A Found Adv* **2015**, *71*, 3-8.
[2] G. M. Sheldrick, *Acta Crystallogr C Struct Chem* **2015**, *71*, 3-8.
[3] O. V. Dolomanov, L. J. Bourhis, R. J. Gildea, J. A. K. Howard, H. Puschmann, *Journal of Applied Crystallography* **2009**, *42*, 339-341.
[4] G. W. T. M. J. Frisch, H. B. Schlegel, G. E. Scuseria, M. A. Robb, J. R., G. S. Cheeseman, V. Barone, G. A. Petersson, H. Nakatsuji, X. Li, M. Caricato, A., J. B. V. Marenich, B. G. Janesko, R. Gomperts, B. Mennucci, H. P. Hratchian, J. V., A. F. I. Ortiz, J. L. Sonnenberg, Williams, F. Ding, F. Lipparini, F. Egidi, J. Goings,, A. P. B. Peng, T. Henderson, D. Ranasinghe, V. G. Zakrzewski, J. Gao, N. Rega, G., W. L. Zheng, M. Hada, M. Ehara, K. Toyota, R. Fukuda, J. Hasegawa, M. Ishida, T., Y. H. Nakajima, O. Kitao, H. Nakai, T. Vreven, K. Throssell, J. A. Montgomery Jr., J., F. O. E. Peralta, M. J. Bearpark, J. J. Heyd, E. N. Brothers, K. N. Kudin, V. N., T. A. K. Staroverov, R. Kobayashi, J. Normand, K. Raghavachari, A. P. Rendell, J. C., S. S. I. Burant, J. Tomasi, M. Cossi, J. M. Millam, M. Klene, C. Adamo, R. Cammi, J., R. L. M. W. Ochterski, K. Morokuma, O. Farkas, J. B. Foresman and D. J. Fox, Gaussian, Inc., Wallingford, CT, **2016**.
[5] C. Adamo, V. Barone, *J. Chem. Phys.* **1999**, *110*, 6158-6170.
[6] G. A. Petersson, M. A. Al-Laham, *J. Chem. Phys.* **1991**, *94*, 6081-6090.
[7] S. Grimme, J. Antony, S. Ehrlich, H. Krieg, *J. Chem. Phys.* **2010**, *132*.
[8] M. H.-G. So Hirata, *Chem. Phys. Lett.* **1999**, *314*, 291-299.
[9] X. Gao, S. Bai, D. Fazzi, T. Niehaus, M. Barbatti, W. Thiel, *J Chem Theory Comput* **2017**, *13*, 515-524.
[10] R. Dennington, T. A. Keith, J. M. Millam, Semichem Inc., Shawnee Mission, KS, **2016**.
[11] A. D. William Humphrey, and Klaus Schulten, *J. Mol. Graph. Model.* **1996**, *14*, 33.
[12] U. o. K. a. F. K. G. (1989–2007), T. G. s. 2007), TURBOMOLE GmbH, **2020**.
[13] S. G. Balasubramani, G. P. Chen, S. Coriani, M. Diedenhofen, M. S. Frank, Y. J. Franzke, F. Furche, R. Grotjahn, M. E. Harding, C. Hättig, A. Hellweg, B. Helmich-Paris, C. Holzer, U. Huniar, M. Kaupp, A. Marefat Khah, S. Karbalaei Khani, T. Müller, F. Mack, B. D. Nguyen, S. M. Parker, E. Perlt, D. Rappoport, K. Reiter, S. Roy, M. Rückert, G. Schmitz, M. Sierka, E. Tapavicza, D. P. Tew, C. van Wüllen, V. K. Voora, F. Weigend, A. Wodyński, J. M. Yu, *J. Chem. Phys.* **2020**, *152*.
[14] C. Hättig, F. Weigend, *J. Chem. Phys.* **2000**, *113*, 5154-5161.
[15] A. Hellweg, S. A. Grun, C. Hattig, *Phys Chem Chem Phys* **2008**, *10*, 4119-4127.
[16] O. S. Lee, M. C. Gather, E. Zysman-Colman, *Digit. Discov.* **2024**, *3*, 1695-1713.
[17] O. S. L. a. E. Zysman-Colman, Chemicus Limited, St Andrews, Scotland **2025**.
[18] N. M. O'boyle, A. L. Tenderholt, K. M. Langner, *J. Comput. Chem.* **2008**, *29*, 839-845.
[19] J. E. Stone, University of Missouri – Rolla **1998**.
[20] J. D. Hunter, *CiSE* **2007**, *8*, 90-95.
[21] M. B. Noel M O'Boyle, Craig A James, Chris Morley, Tim Vandermeersch & Geoffrey R Hutchison, *J. Cheminform.* **2011**, *3*, 33.
[22] N. M. O'Boyle, G. R. Hutchison, *Chem. Cent. J.* **2008**, *2*.
[23] C. S. B. a. B. D. M. Colette McDonagh, *Chem. Rev.* **2008**, *108*, 400-422.
[24] N. Velusamy, A. Binoy, K. N. Bobba, D. Nedungadi, N. Mishra, S. Bhuniya, *Chem Commun (Camb)* **2017**, *53*, 8802-8805.
[25] W. E. G. Neil G. Connelly, *Chem. Rev.* **1996**, *96*, 877-910.
[26] C. M. Cardona, W. Li, A. E. Kaifer, D. Stockdale, G. C. Bazan, *Adv. Mater.* **2011**, *23*, 2367-2371.
[27] G. Gonzalez, M. Arévalo, A. Chiappone, E. Martínez Campos, C. F. Pirri, I. Roppolo, P. Bosch, *Macromol. mater. eng.* **2023**, *308*.
[28] S. Miralles-Comins, M. Zanatta, S. G. Embid, M. Alleva, A. Chiappone, I. Roppolo, S. G. Mitchell, V. Sans, *Device* **2024**, *2*.
[29] M. Armandi, B. Tosetto, I. Roppolo, A. Chiappone, *Adv. Manuf.* **2025**, *2*.
[30] A. Chiado, G. Palmara, A. Chiappone, C. Tanzanu, C. F. Pirri, I. Roppolo, F. Frascella, *Lab Chip* **2020**, *20*, 665-674.

[31] S. Villata, M. Canta, D. Baruffaldi, A. Pavan, A. Chiappone, C. F. Pirri, F. Frascella, I. Roppolo, *Biomater Sci* **2023**, *11*, 2950-2959.
[32] G. Gonzalez, A. Chiappone, K. Dietliker, C. F. Pirri, I. Roppolo, *Adv. Mater. Technol.* **2020**, *5*.
[33] A. Goswami, A. M. Umarji, G. Madras, *Polym. Adv. Technol.* **2012**, *23*, 1604-1611.
